# Modeling the Interaction between Quinolinate and the Receptor for Advanced Glycation End Products (RAGE): Relevance for Early Neuropathological Processes

**DOI:** 10.1371/journal.pone.0120221

**Published:** 2015-03-10

**Authors:** Iris N. Serratos, Pilar Castellanos, Nina Pastor, César Millán-Pacheco, Daniel Rembao, Ruy Pérez-Montfort, Nallely Cabrera, Francisco Reyes-Espinosa, Paulina Díaz-Garrido, Ambar López-Macay, Karina Martínez-Flores, Alberto López-Reyes, Aurora Sánchez-García, Elvis Cuevas, Abel Santamaria

**Affiliations:** 1 Departamento de Química, Universidad Autónoma Metropolitana-Iztapalapa, México D.F., México; 2 Laboratorio de Aminoácidos Excitadores, Instituto Nacional de Neurología y Neurocirugía, *Manuel Velasco Suárez*, SSA, México D.F., México; 3 Departamento de Ingeniería Eléctrica, Universidad Autónoma Metropolitana-Iztapalapa, México D.F., México; 4 Facultad de Ciencias, Universidad Autónoma del Estado de Morelos, Cuernavaca, Morelos, México; 5 Instituto de Ciencias Físicas, Universidad Nacional Autónoma de México, Cuernavaca, Morelos, México; 6 Neuropatología, Instituto Nacional de Neurología y Neurocirugía, *Manuel Velasco Suárez*, México D.F., México; 7 Departamento de Bioquímica y Biología Estructural, Instituto de Fisiología Celular, Universidad Nacional Autónoma de México, México D.F., México; 8 Laboratorio de Líquido Sinovial, Instituto Nacional de Rehabilitación, SSA, México D.F., México; 9 Neurochemistry Laboratory, Division of Neurotoxicology, National Center for Toxicological Research/FDA, Jefferson, Arkansas, United States of America; University of Miami, UNITED STATES

## Abstract

The receptor for advanced glycation end products (RAGE) is a pattern-recognition receptor involved in neurodegenerative and inflammatory disorders. RAGE induces cellular signaling upon binding to a variety of ligands. Evidence suggests that RAGE up-regulation is involved in quinolinate (QUIN)-induced toxicity. We investigated the QUIN-induced toxic events associated with early noxious responses, which might be linked to signaling cascades leading to cell death. The extent of early cellular damage caused by this receptor in the rat striatum was characterized by image processing methods. To document the direct interaction between QUIN and RAGE, we determined the binding constant (K_b_) of RAGE (VC1 domain) with QUIN through a fluorescence assay. We modeled possible binding sites of QUIN to the VC1 domain for both rat and human RAGE. QUIN was found to bind at multiple sites to the VC1 dimer, each leading to particular mechanistic scenarios for the signaling evoked by QUIN binding, some of which directly alter RAGE oligomerization. This work contributes to the understanding of the phenomenon of RAGE-QUIN recognition, leading to the modulation of RAGE function.

## Introduction

Neurodegenerative disorders represent one of the most important causes of disability in the world. As a growing pathological event, the incidence of neurological disorders is expected to increase in the near future. Neurodegeneration is an incapacitating multifactorial process affecting one or several neuronal nuclei in the brain, and is characterized by massive loss of neuronal cells [1]. Among the factors involved in neurodegeneration are excitotoxicity, oxidative stress, inflammatory events, mitochondrial dysfunction and energy depletion, protein misfolding and aggregation, damaged cell signaling, apoptosis and necrosis [2–4]. In some cases, such as Huntington’s disease (HD), heritable mutations are responsible for dysfunctional proteins that can trigger deadly cascades, ultimately leading to selective neuronal cell death.

The kynurenine pathway (KP) for tryptophan degradation is one of the most important routes for the production of metabolic precursors [5–7]. This KP is responsible for the degradation of around 90% of the tryptophan involved in the synthesis of NAD^+^. However, metabolic alterations in this route can result in the accumulation of the neurotoxic metabolite quinolinate (QUIN or 2,3-pyridinedicarboxylate) [8]. QUIN is a well-known N-methyl-D-aspartate receptor (NMDAr) agonist that produces excitotoxic events in the brain [9,10]. The persistent activation of glutamatergic NMDAr and the concomitant excitotoxic event induced by QUIN have been linked with a cascade of toxic processes that ultimately kill neuronal cells. These processes include oxidative stress, inflammation, neurochemical deficits, and energy depletion, among others [8]. Indeed, due to evidence showing metabolic alterations in KP and enhanced levels of QUIN in the Central Nervous System (CNS), QUIN has been postulated as a good candidate to explain neurodegenerative events in different neurological, inflammatory and infectious disorders, such as HD, hepatic encephalopathy, AIDS-dementia complex, and Alzheimer’s disease [8]. QUIN also represents an important tool at the experimental level to mimic the neurochemical, cellular, morphological, biochemical and behavioral features observed in HD when injected in the striatum of rats [9,10]. Considering the endogenous nature of this metabolite and its many potential implications in neurological disorders, the characterization of the toxic mechanisms underlying QUIN toxicity constitutes a relevant issue for a better understanding of human pathologies. In particular, early toxic events that will be responsible for late toxicity are of major relevance to understand neurodegenerative processes. One of these mechanisms could be related with the stimulation of deadly cascades toward the direct activation of different membrane receptors, independently of an action on NMDAr. Our group has recently described preliminary evidence of the involvement of the receptor for advanced glycation end products (RAGE) in the toxic pattern exerted by QUIN in the rat striatum [11]. We were able to demonstrate that RAGE expression was increased by QUIN, comprising the trigger of a pro-inflammatory pathway; however, whether QUIN might also interact directly with RAGE to enhance toxicity is a question deserving further investigation.

RAGE is a transmembrane protein with different ligands that have been associated with various diseases (inflammatory disorders, diabetes, cancer, and neurodegenerative diseases, among others) [12–18]. RAGE is known to induce cellular signaling events upon binding to ligands such as advanced glycation end products (AGEs) [19,20], amyloid-fibrils [21,22], amphoterin or high mobility group box-1 (HMGB1) [23–25], and members of the S100 protein family [26–28]. In previous studies RAGE has been characterized as a protein weighing 45 kDa, consisting of 404 amino acid residues, and composed of a variable-type (V) and two constant-type domains (C1 and C2), a transmembrane domain, and a cytosolic tail [22,29]. The latter is essential for the activation of nuclear transcription factor kappa B (NF-ĸB) [27], a factor frequently involved in inflammatory processes and deadly events in the brain. In recent years, growing interest has been given to characterize this receptor as a therapeutic target, since RAGE is involved in different pathologies; therefore, the signaling associated with the stimulation of this protein is of major relevance for biomedical research.

The extracellular domain of RAGE, also known as sRAGE (or soluble RAGE) is a sensor that competes for ligands with the membrane-bound form of RAGE, thus blocking the RAGE signaling cascade. In fact, sRAGE has been used in rat models of various central and peripheral diseases as a therapeutic strategy to reduce tissue damage associated with inflammation [30,31]. sRAGE exhibits a molecular mass of 35 kDa, as well as a sequence of 234 amino acid residues, and consists of three well characterized domains: V, C1 and C2 [25,28]. In addition, complementary biochemical studies have suggested a role of self-association (dimerization) for RAGE function [27,32].

Various human RAGE structures have been determined by X-ray crystallography as well as NMR spectroscopy, and deposited in the Protein Data Bank [33] (Table 1). These structures show large hydrophobic and positively charged regions on the V domain surface, possibly having direct implications for ligand binding to RAGE. In turn, RAGE VC1 and VC1C2 crystallized as dimers [32], and most ligands bind at the opposite face of the V domain used for dimerization [34]. The large positive charge in the dimer can be neutralized by nucleic acids [35] or by glycosaminoglycans [34]. All these structures point to the V domain as the main ligand-interacting region of sRAGE, but it remains possible that the C1 domain can also participate in AGE binding.

**Table 1:** RAGE crystallographic structures deposited in Protein Data Bank (PDB).

ID PDB	Domain	Citation	Method
2E5E	V domain	Journal to be published	NMR
2ENS	C2 domain	Journal to be published	NMR
3CJJ	VC1 domains	Koch, M. *et al* 2010 [27]	X-RAY DIFFRACTION (1.85 Å)
303U	VC1 domains with maltose	Park, H. *et al* 2010 [29]	X-RAY DIFFRACTION (1.50 Å)
2L7U	V domain with CEL*	Xue, J *et al* 2011 [36]	NMR
2LMB	C-terminal (ctRAGE)	Rai, V *et al* 2012 [37]	NMR
2LE9	C2 domain with S100A13	Journal to be published	NMR
4LP4	VC1 domains	Yatime, L *et al* 2013 [32]	X-RAY DIFFRACTION (2.40 Å)
4LP5	VC1 domains	Yatime, L *et al* 2013 [32]	X-RAY DIFFRACTION (3.80 Å)
4OI7	VC1 domains with DNA	Sirois, C.M. *et al* 2013 [35]	X-RAY DIFFRACTION (3.10 Å)
4OI8	VC1 domains with DNA	Sirois, C.M. *et al* 2013 [35]	X-RAY DIFFRACTION (3.10 Å)
4IM8	Mouse RAGE with heparin	Xu, D. *et al* 2013 [34]	X-RAY DIFFRACTION (3.50 Å)
4OF5	VC1 domains	Yatime, L *et al* 2014 [38]	X-RAY DIFFRACTION (2.80 Å)
4OFV	VC1 domains	Yatime, L *et al* 2014 [38]	X-RAY DIFFRACTION (3.10 Å)
2MOV	V domain with methylglyoxal	Xue, J. *et al*, 2014 [39]	NMR

*N(Ɛ)-carboxy-ethyl-lysine (CEL)

**Fig 1:**
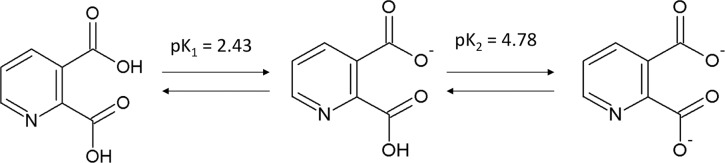
Molecular structure of quinolinic acid (QUIN). QUIN and its conversion to a dianionic stucture (pK1 = 2.43 and pK2 = 4.78) [40] at pH 7.4. Each structure was built up with the program ACD/ChemSketch Freeware (http://www.acdlabs.com/resources/freeware/chemsketch/).

In this study, our interest was focused on the characterization of some toxic events associated with RAGE-induced early noxious responses, which may be linked to signaling cascades, further leading to cell death induced by the endogenous neurotoxic metabolite QUIN, and its involvement in the up-regulation of and/or interaction with RAGE. QUIN has two pKa values (pKa_1_ = 2.43 and pKa_2_ = 4.78),[40] so it will be fully ionized at pH 7.4 (Fig. 1), which is important for the molecular recognition of receptors with positive charge such as RAGE. In fact, a recent study indicated that QUIN induces up-regulation of RAGE, leading to the activation of the NF-ĸB pathway, altered gene expression, nitrosative stress, metabolic alterations and premature cell damage [11]. These results suggested that the up-regulation of RAGE may play a role in the early stages of QUIN toxicity, which is mostly attributed to a direct action of QUIN on NMDAr and a further indirect activation of RAGE. However, the possibility that a negative molecule such as QUIN could engage in direct chemical interactions together with RAGE might offer interesting alternative explanations for this model. The evidence collected from previous studies suggests that RAGE is a pathogenic factor potentially involved in neurodegenerative diseases; however, we are far from having characterized the precise role of RAGE in the pathogenic pattern evoked by QUIN, especially when considering the potential chemical interactions that both QUIN and RAGE can display in the mammalian brain.

Therefore, with the aim to search for alternative mechanisms of QUIN toxicity through a direct interaction with RAGE, in this work we performed immunoblotting, immunohistochemical and immunofluorescent assays for the identification of the cellular damage caused by the interactions of this receptor and QUIN in the rat striatum. To document the direct interaction of RAGE with QUIN, we explored the molecular recognition between RAGE domains and QUIN, determining the binding constant (K_b_) of RAGE (VC1 domain) with QUIN by fluorometric titration. We also simulated the interaction between RAGE and QUIN by flexible docking for both human and rat VC1 dimer structures. Given that QUIN is a small ligand that does not oligomerize, it cannot signal by engaging in multivalent contacts with multiple RAGE molecules, as proposed for AGE-modified proteins [36,39], HMGB1 [23–25] and S100B [26–28]. Hence, we analyzed the docking poses with the intention of proposing mechanistic models for signal transduction. Three main scenarios emerged from this analysis: QUIN may compete with other RAGE ligands, it may help to stabilize the RAGE dimer, or it may alter the dynamics of the neck region between the V and C1 domains. This study is complementary to previous reports employing the toxic model induced by QUIN in rats by unilateral striatal lesions.

## Materials and Methods

### Reagents

QUIN was obtained from Sigma-Aldrich (St-Louis, MO, USA). Primary antibodies against RAGE (Ab3611 for immunofluorescent and immunoblot assays) and secondary antibodies (anti-mouse) were purchased from Abcam (Cambridge, MA, USA). NeuN (monoclonal mouse antibody, MAB377 Clone A60, 1:200) was from EMD Millipore Co. (Billerica, MA, USA). Another RAGE antibody (Ab7764, Abcam) was specifically employed only for immunohistochemical purposes. All other reagents were obtained from known commercial sources, and described below.

### Animals

A total of 70 male adult Wistar rats weighing 300 g were used throughout the study. Rats were housed with a standard diet *ad libitum*, and circadian rhythms were synchronized with a 12-h:12-h light-dark cycle in the animal facility environment, under constant conditions of temperature (25±3°C) and humidity. All experimental procedures with animals were performed according to the ‘‘Guidelines for the Use of Animals in Neuroscience Research” from the Society of Neuroscience, and approved by the Institutional Animal Care and Use Committee of the Instituto Nacional de Neurología y Neurocirugía (IACUC/INNN, Code (607)65/09), in compliance with the ARRIVE guidelines.

### Animal treatments and striatal lesions with QUIN

Animals (6 per group) were randomly assigned to four experimental groups: Groups I (Sham) and II (QUIN) received one i.p. injection of vehicle (PBS), whereas Groups III (SAC) and IV (SAC+QUIN) received SAC (300 mg/kg, i.p.). Groups I and III also received a single 2-μL intrastriatal infusion (in the caudate-putamen) of PBS (phosphate-buffered saline as vehicle), while Groups II and IV received a similar volume of 240 nM QUIN dissolved in PBS (pH 7.4). Thirty min after the SAC administration, animals were anesthetized with sodium pentobarbital (45 mg/kg, i.p.), placed on a stereotaxic frame (Stoelting Co., Wood Dale IL, USA) with the incisor bar fixed at 3.0 mm below the interaural line, and unilaterally injected with QUIN into the right striatum using the following coordinates: +1.0 mm anterior to bregma, +3.2 mm lateral to bregma, and -4.2 mm ventral to the dura, according to the brain atlas of Paxinos and Watson [41]. Rats were injected into the striatum using a 10-μl Hamilton microsyringe. The needle was left in place for an additional 5 min and then slowly withdrawn in 1 min. Animals were sacrificed after 30, 60 and 120 min post-lesion for Western blot, immunofluorescence and immunohistochemical analyses.

### Cell lysis and Western blot

Total proteins were isolated from brain tissue with M-PER-supplemented extraction reagent (Pierce Chemical, Rockford, Illinois). Western blot procedures were conducted as reported previously [42]. Inmunodetection was performed using a primary anti-RAGE antibody at 1:1,000 (according to datasheet specifications). The antibody employed to detect RAGE is sensitive to the intracellular RAGE domain (recognizing the amino acids sequence 362–380 in rat, the fragment of RAGE directly bound to the transmembrane domain), therefore allowing the recognition of full-length (FL) RAGE. The secondary anti-rabbit IgG antibody (Abcam, 1:10,000 dilution) was conjugated with horseradish peroxidase. Blots were revealed using Immobilon Western Chemiluminescent HRP Substrate (Millipore Corporation, USA). The blots were scanned with an Image Station 4000 mm Pro Kodak from Carestream (Rochester, NY). Results are expressed relative to β-actin protein, and relative quantification was performed using the Carestream Molecular Imaging Software. Protein content was determined using a bicinchoninic acid (BCA) kit (Pierce Chemical, USA), according to the manufacturer’s instructions.

### Histological and histochemical assays

Rat brains were obtained at different times (30, 60 and 120 min) after the intrastriatal lesion. The perfused brains were fixed in paraformaldehyde (4%) for 48 h. Thereafter, tissue samples were washed in distilled water, dehydrated with gradual alcohols (70, 80, 96 and 100%) for 60 min, and exposed to xylol for another 60 min. The whole process was carried out in an automatic tissue processer. Samples were then embedded in paraffin. Coronal sections (5 μm thick) were serially obtained every 5 μm, using a Leica microtome. Tissue slides were deparaffined in xylol and rehydrated in gradual alcohols in descendent order (100, 96 and 80%), to finally reach distilled water. Each step lasted 2 min. Histological processing considered the Hematoxylin—eosin (H&E) protocol, whereas the histochemical assay comprised peroxidase-based immunoreactivity against the RAGE and NeuN proteins.

For H&E, slides were exposed for 2 min to Gill’s hematoxilin, washed with distilled water, processed with ammonium water to turn blue and washed once again in distilled water. Thereafter, slides were contrasted in an eosin solution for 1 min, dehydrated with ethanol (96 and 100%), and clarified with xylene. Each procedure was repeated twice and for 2 min. Slides were finally mounted in synthetic resin for further observation under microscopy.

For histochemical purposes, endogenous peroxidase activity was blocked with a hydrogen peroxide solution (3%). The slides were then washed in PBS (pH 7.4), and antigenic recovery was made with a citrate solution (0.1 M, pH 6.0) for 4 min. The sections were washed once again in PBS and exposed to an IgGs-free bovine serum albumin solution (1%) diluted in PBS, to be further added with 100 μl of the primary anti-RAGE antibody (1:20 in PBS) in left overnight in a wet chamber (4°C). After a new wash with PBS, the second antibody was added for 30 min in the wet chamber at room temperature, and washed again with PBS. Thereafter, the chromogenous DAB (0.05% in PBS) was added to induce a brown staining that revealed the reactivity of the antibody with the tissue. Slides were washed in distilled water, dehydrated in gradual alcohols (96 and 100%), clarified with xylene and mounted with synthetic resin for microscopic observation.

For the case of NeuN immunoreactivity, antigenic recovery was done for 30 min. The primary anti-NeuN antibody (1:200) was added to the slides and incubation was done overnight at 4°C. The second antibody (biotinylated) was added for 1 h and a further incubation was made with streptavidin peroxidase, to further wash with PBS. Staining was contrasted against hematoxylin.

### Immunofluorescence

Immunofluorescence stains were performed on slides from brain sections of paraffin blocks obtained from the histopathological study and treated with the polyclonal rabbit primary antibody to identify the RAGE protein and its possible co-localization with NeuN. Previously, the samples were deparaffinized and slides were fixed with 4% paraformaldehyde (PFA) for 15 min, to further undergo 3 washes of 10 min each, with PBS (pH 7.2). Slides were then incubated with 0.6 M glycine for 20 min to remove autofluorescence of aldehydes, then washed with 0.5% PBA (PBS albumin) three times. Following blocking with 1% PBA for 20 minutes to remove nonspecific reactions of the antibodies, they were washed three times with 0.5% PBA. Stains were made diluting 1:100 polyclonal rabbit primary antibody for RAGE in 1% PBA and/or 1:200 monoclonal primary antibody for NeuN, both for 1 h at 4°C, then washed three times with PBA. The anti-rabbit secondary antibody (Alexa-488) was diluted 1:400 and incubated for 45 min. Subsequently, slides were washed with 1% PBA. Nuclei were stained blue with DAPI (Vectashield CA, USA). Images were obtained using a fluorescence microscope (Floid Cell Imaging Station, Life Technologies, USA).

### Cell counting

Cell counting was carried out using the public-use program "ImageJ" (from the National Institutes of Health, USA) for the immunohistochemistry and immunofluorescence studies. Previously, image segmentation was performed by establishing a threshold for each color component of the RGB (red, green and blue) image. Lighting homogenization was carried out by the 'Bottom hat' method [43]. The segmentation method and the lighting homogenization were implemented in Matlab software (version 7.10.0) [44]. The segmentation method was also used for the quantification of the RAGE and RAGE plus NeuN expression by the immunofluorescence assay.

### Molecular biology

The gene for the receptor for advanced glycation endproducts (RAGE) was sent to Gene Script Co. to synthesize the VC1 region, corresponding to amino acids 23 to 243. This gene was subcloned in the NdeI-BamHI sites to the 3rd modified pET plasmid with a six His tag and an enterokinase recognition site.

### Protein expression and purification

The RAGE VC1 fragment was overexpressed in *E*. *coli* strain Shuffle T7 (New England Biolabs). The bacteria were grown at 30°C to an OD 600 nm (~0.7), induced with 1 mM IPTG and allowed to express overnight. The cells were lysed at 4°C in 50 mM NaH_2_PO_4_, 1.5 M NaCl, and 10 mM imidazole at pH 8.0, followed by sonication. The clarified lysate was purified in HisTrap HP column (GE Healthcare), equilibrated with the corresponding lysis buffer and eluted with a linear gradient containing 50 mM NaH_2_PO_4_, 1.5 M NaCl, and 1 M imidazole, pH 8.0 in a Superdex 200 10/300 Molecular Filtration Column. After histidine—purifying the histidine-tagged protein, the eluted fraction was dialyzed against 20 mM glycine and 133 mM NaCl at pH 9.0, to improve the stability of VC1. The protein concentration was obtained by absorption measurements (at 280 nm) using an extinction coefficient of 33710 M^-1^*cm^-1^ [45]. The estimation of QUIN concentration was carried out according to a previous report [46]. The ultraviolet absorption spectra of the protein were corrected for light scattering [47] and centrifuged at 10,000 r.p.m. (9300 x *g*) for 20 min before protein quantification.

### Fluorometric titration

Fluorescence spectra were obtained with an ISS K2 spectrofluorometer (Urbana, USA); the analyses were carried out in 20 mM Tris-base, 133 mM NaCl, pH 7.4 and 20 mM glycine, 133 mM NaCl, pH 9.0. Both analyses were performed in buffers with ionic strength of 0.15 M and at 25°C. Emission spectra were measured with an excitation and emission bandwidths of 16 nm and 8 nm, respectively. The fluorescence emission intensity was recorded at 320 nm during 3 min, using an excitation wavelength of 280 nm. Cells with 1 cm path lengths were used and samples were continuously stirred during measurements. A 0.1 μM VC1 solution (2 mL) was titrated with successive volumes of 0.02 μM and 0.2 μM QUIN. We adjusted the titration data set according to previous authors [48,49], assuming that the fluorescence intensities of the emitting species (free VC1 and VC1–QUIN complex) are additive, according to Equation 1,

$$Y=\left(\frac{a}{2E_{t}}\right)\left\lceil \left(E_{t}+x+K_{d}\right)-\sqrt{{\left(E_{t}+x+K_{d}\right)}^{2}-4xE_{t}}\right\rceil$$ Eq (1)

where *x* is the total concentration of inhibitor in the cell, *Et* represents the total concentration of VC1, *K*_*d*_ is the dissociation equilibrium constant (*K*_*d*_ = 1/ *K*_*b*_) and *a* denotes the asymptotic value to which *Y* tends at high *x* values. *Y = 1-F/F*_*t*_, were *F* is the overall fluorescence intensity after each addition of QUIN and *F*_*t*_ is the fluorescence of free VC1 at the corresponding concentration, respectively. We obtained *K*_*d*_ and *a* as fitting parameters from a nonlinear least-squares regression, using the program Origin (MicroCal Inc. Northamppton, MA, USA).

### VC1C2 Rat RAGE model

The VC1C2 model of *Rattus norvegicus* (GENBANK ID: ADX07274.1, starting at residue 22) was built using the I-TASSER server [50,51]. The model used was the one with the highest C-score (1.03) and TM-score (0.85+-0.08). The model was immersed in a large box of TIP3 water and 0.15 M KCl, and run for 1 ns at 300 K in NAMD [52] with the CHARMM36 [53] potential. The default parameters for non-bonded interactions (PME) and periodic boundary conditions were used. The last snapshot was used for building the dimer model.

### Homodimer models

The VC1C2 RAGE homodimers for both human and rat were built using the structure ID: 4LP5 [32] from PDB. The full VC1C2 homodimer was constructed superposing chain A of 4LP5 with chain B, using the MatchMaker module implemented in Chimera UCSF [54]. These models were briefly energy-minimized with 100 steps of the steepest-descent method in CHARMM38b2 [55], with the CHARMM36 [53] potential, to relieve remaining steric clashes.

### Docking assays

The human (4LP5) and rat homodimers, prepared as described above, were employed for docking with QUIN. We used flexible docking for the ligand only, restricting the docking area to the VC1 domains, for comparison with the fluorescence experiments. Docking was performed using the program AutoDock Vina [56], requiring 20 poses with an exhaustiveness of 50. Binding energies ranged from-6.2 kcal/mol to -4.7 kcal/mol in human and from -5.0 kcal/mol to -4.6 kcal/mol in rat.

## Results

### The striatal infusion of QUIN to rats produced early morphological alterations near to the lesion site

**Fig 2:**
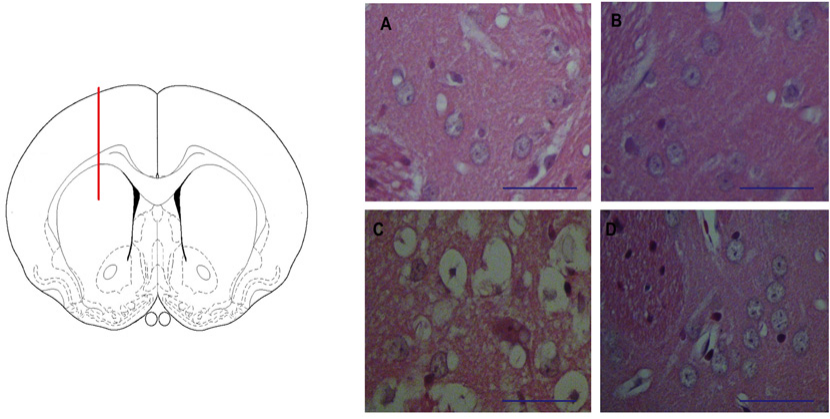
The striatal lesion induced by QUIN in rats. In the left panel, a schematic representation of the lesion site (dorsal striatum) in a drawing of a coronal section of the rat brain is depicted. Red line represents the needle trajectory. In the right panel, **A-D** micrographs (40X) show striatal sections stained with Haemotoxylin & Eosin (Bar size 100 μm), where **A** corresponds to Sham (mechanically lesioned right striatum); **B** is the contralateral (unlesioned) striatum in the same animal; **C** shows the right striatum lesioned by QUIN (240 nmol/μl); and **D** depicts the contralateral unlesioned striatum from the same QUIN-infused rat. Sham and unlesioned striata (**A**, **B** and **D**) show neuronal cells without structural alterations, whereas the QUIN-lesioned striatum (**C**) exhibits morphological alterations nearby the lesion site that were characterized by diffuse vacuolization, pyknosis, edema and neuropil degeneration.

With the aim to test if the striatal infusion of QUIN to rats is readily responsible for major morphological alterations at times as short as 120 min post-lesion, the striatal tissues of QUIN-lesioned rats were collected and processed for histological assessments. In Fig. 2, a scheme showing the lesion site in the rat brain is depicted (left panel), accompanied by four micrographs corresponding to striatal coronal sections of Sham and QUIN-lesioned rats (A-D). For comparative purposes, we included sections of the lesioned side (A and C) vs. the unlesioned side (B and D). The general appearance of the striata in A, B and D shows preserved cell bodies, with well-conserved neuropil and fibers. In contrast, the striatal tissue surrounding the QUIN-induced lesion (C) depicts vacuolization, damaged cell bodies characterized by pyknosis and edema, and degenerated neuropil. It is noteworthy that this morphological lesion resembles the tissue alterations that have been commonly described for the striata of QUIN-lesioned rats at longer post-lesion times (days) [57]. The explanation for this early scenario would be the area from which the image has been obtained, that corresponds to a section nearby the lesion (injection) site. Given that all sections (either from Sham or QUIN-treated rats) were systematically obtained at the same distances from the needle trajectory (50 μm), the lesion shown in C cannot correspond to a mere mechanical process, despite the fact that at 120 min after the lesion, damage was not widely extended along the striatum (image not shown). Therefore, the early degenerative events induced by QUIN and comprising the morphological alterations can be produced at times as short as 120 min, but they are remitted to small areas surrounding the lesion.

### The intrastriatal infusion of QUIN to rats did not reduce the number of neuronal cells at short times post-lesion

**Fig 3:**
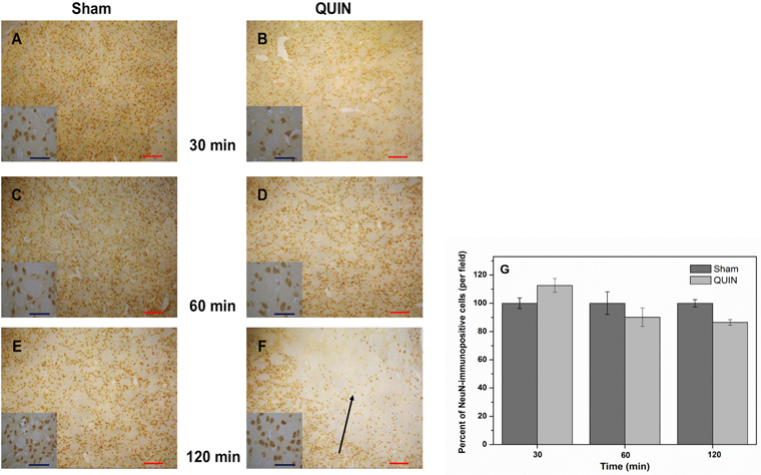
Histochemical alterations produced by QUIN in rats. Peroxidase-based immunohistochemical staining of neuronal cells (NeuN) in striatal coronal sections (10X) of Sham (**A**, **C** and **E**)- and QUIN (**B**, **D** and **F**)- treated animals at different post-lesion times (Bar size 100 μm). Details of cell morphology for each treatment are shown in small squares (40X). The segmentation method was employed for cell counting, and expressed as immunopositive cells. In **A**, **C** and **E**, normal appearance of the striata with normal cell densities are shown. In **B**, **D** and **F**, the striatal appearance at 30, 60 and 120 min post-lesion is presented. Also in **F**, a considerable loss of neuronal density (indicated by arrow) can be appreciated close to the lesion site. In **G**, the numbers of immunopositive cells (mean percent ± SD), determined by the segmentation method, are graphically represented.

Fig. 3 depicts the density of neuronal cells in the striata of Sham and QUIN-treated rats at three different times after the lesion. Neuronal cells were marked selectively with the NeuN antibody. No observable differences were found between Sham and QUIN-lesioned rats at 30 (A and B, respectively) or 60 min (C and D, respectively). Although at 120 min post-lesion (E and F), a moderate but non-significant loss of striatal neurons was observed surrounding the lesion site (13% below the control), in the best scenario, this decrease can be interpreted as a slight tendency to start the degenerative process to be evidenced at longer times. Details of cell morphology can be appreciated in the small squares in the figure. As expected, at these short times of QUIN exposure, surviving neurons keep a similar cytoarchitecture in Sham and QUIN-lesioned rats. This trend was confirmed when neuronal cells were quantified employing a segmentation method for image processing (G), demonstrating that only at 120 min post-lesion, QUIN was capable of inducing a moderate loss in the number of striatal cells.

### QUIN induced enhanced RAGE immunoreactivity in striatal tissue after a short time of exposure

**Fig 4:**
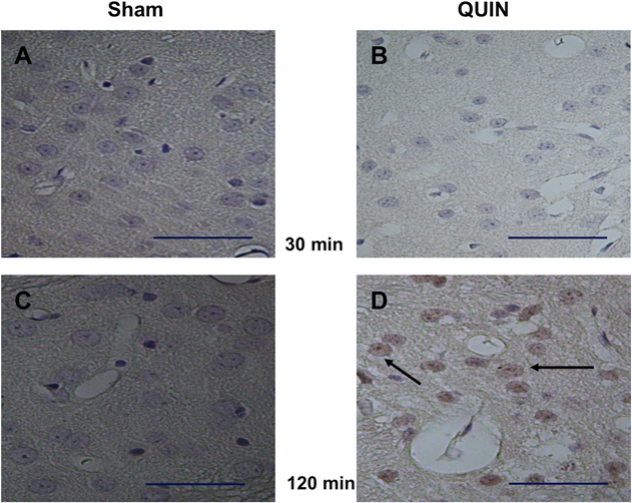
Histochemical labeling of RAGE-positive cells. Peroxidase-based immunohistochemical staining of RAGE-positive cells in striatal coronal sections (40X) of Sham (**A** and **C**)- and QUIN (**B** and **D**)- treated animals at 30 and 120 min post-lesion, respectively (Bar size 100 μm). In **D**, a prominent reactivity of cells to RAGE (indicated by arrows) is observed.

The slight but significant early changes in morphology induced by QUIN, together with the moderate loss of neuronal cells produced by this toxin at 120 min after the lesion, matched with an early increased cellular immunoreactivity to the RAGE protein (Fig. 4). This effect was observed at 120 (C vs. D), but not at 30 min after the lesion (A vs. B) in wide coronal sections. In contrast to Sham (C), immunopositive cells in QUIN-lesioned striata (D) exhibited a clear brown cytoplasmic peroxidase-based mark, evidencing reactivity inside the cell body. RAGE expression in QUIN-lesioned striata at 120 min suggests an early role of this protein for the triggering of toxic cascades that will further lead to the late degenerative pattern elicited by the neurotoxin in the brain.

### Striatal RAGE localization was increased by QUIN, as determined by immunofluorescence

**Fig 5:**
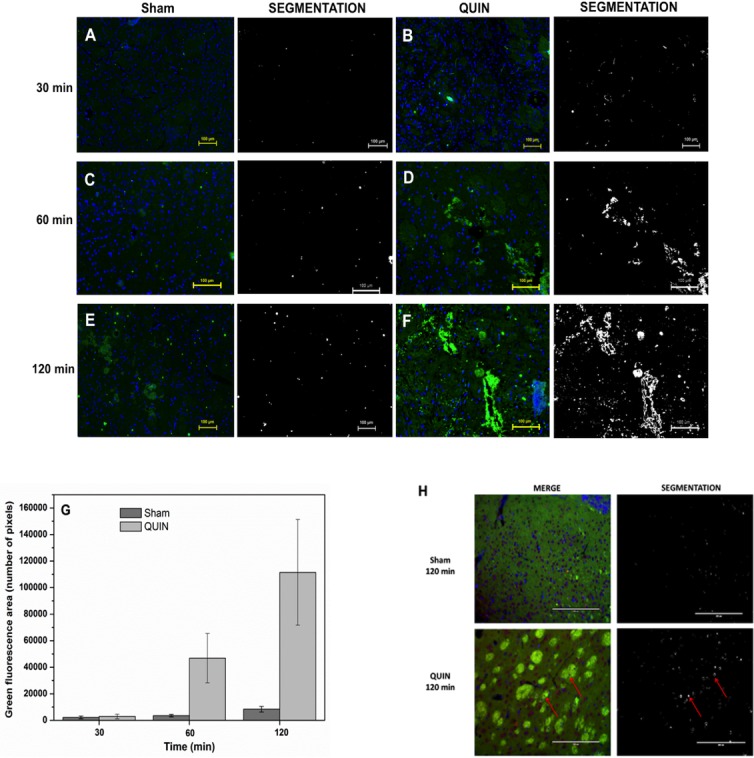
Fluorescent labeling of RAGE. Immunofluorescent localization of intracellular RAGE in the striatum of Sham (**A**, **C** and **E**)- and QUIN (**B**, **D** and **F**)-treated rats at different (30, 60 and 120 min) post-lesion times. RAGE is marked in green and cell nuclei (DAPI staining) in blue. Prominent immunofluorescence against RAGE was detected in **D** and **F**. In **G**, the density of immunopositive cells (mean green area ± SD) is graphically represented. In H, merge images showing the co-staining of nuclei (DAPI in blue and violet), neuronal cells (NeuN in red and violet) and RAGE (in green) in Sham and QUIN-lesioned striata at 120 min after the lesion. Arrows indicate triple co-localization. For all images, additional columns showing the marking process for quantification of the fluorescent labeling by the segmentation method are shown sidewise the treatment columns.

In order to confirm our findings described in Fig. 4, immunofluorescence assessments were performed to estimate the degree of RAGE expression in the striatum of rats receiving a single infusion of QUIN, at different post-lesion times (Fig. 5). The intensity of the green fluorescent mark corresponding to RAGE revealed that the amount of this protein is significantly increased at 120 min after the lesion, when compared with Sham animals (F vs. E, respectively). This observation was confirmed through the segmentation method images (contrasted panels also in Fig. 5) and matches with the findings described in Fig. 4, thus confirming an early role of RAGE in the toxic model of QUIN.

Moderate immunoreactivity for RAGE was observed in the Sham and QUIN sections at 30 (A and B, respectively) and 60 min post-lesion (C and D, respectively). The enhanced immunoreactivity against RAGE induced by QUIN was confirmed quantitatively through the green fluorescence area counting (number of pixels in G), where it was evident that, at 120 min post-lesion, an intense reactivity was induced by the toxin (1228% above the control).

In addition, co-staining of RAGE and NeuN (merge) are shown in panel H for both Sham and QUIN-treated animals at 120 min after the intrastriatal lesion. The striata of Sham rats (upper squares) exhibited a considerable number of blue (DAPI) and violet (NeuN+DAPI) structures, suggesting the presence of both non-neuronal and neuronal cells, respectively. Limited green fluorescence was observed, as expected. The segmentation method confirmed a very low co-stain for the three colors in Sham, supporting the concept that RAGE expression is limited in neuronal cells upon normal conditions (right upper square). In the lower squares, a QUIN-lesioned striatum (representative) shows a slightly lower number of neuronal cells when compared with Sham, and an intense RAGE expression mostly located in complexes of fibers, as well as surrounding some nuclei. In addition, several violet nuclei (neuronal cells) are close to green stains, suggesting an enhanced reactivity of neurons to RAGE (arrows indicate triple co-stain). This observation was confirmed by the segmentation method (right lower square), where arrows indicate triple co-localization.

### QUIN-induced early RAGE expression is dependent on oxidative stress

**Fig 6:**
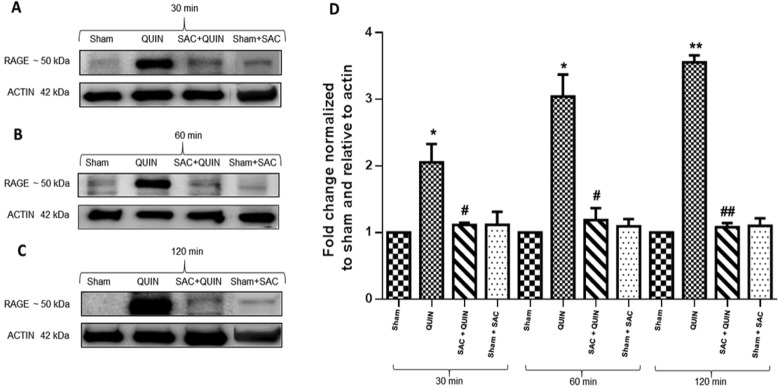
Immunoblot detection of RAGE. QUIN-induced intracellular RAGE expression was suppressed by the antioxidant S-allylcysteine (SAC). In **A**, **B** and **C**, RAGE expression at 30, 60 and 120 min post-lesion in whole striatal extracts (35 μg protein/lane), respectively, is shown. Bands correspond to full-length (FL-RAGE) form of the protein (found around 50 kDa). Results are expressed as fold change compared with Sham. Each image represents three independent experiments. In **D**, the plot shows relative RAGE expression normalized to actin. Significant differences against Sham (*P<0.05 and **P<0.01) or QUIN (^#^P<0.05 and ^##^P<0.01) were considered. One-way ANOVA followed by Tukey’s test for multiple comparisons was used.

Fig. 6 depicts the effects of QUIN and S-allylcysteine (SAC) on the protein levels of the full-length RAGE form (FL-RAGE) in rat striatal tissue at different times after the lesion. The exposure of rats to QUIN induced an increase in RAGE expression at all post-lesion times tested (2-fold (p<0.05), 3-fold (p<0.05) and 3.5-fold (p<0.01) vs. Sham group at 30, 60 and 120 min, respectively; A-C). A compiled graphic expression of these results is presented in D. This effect is clearly due to an oxidative stress-component, as evidenced by the reduction of RAGE expression induced by SAC in QUIN-treated animals (A-C), bringing the densitometric values of RAGE back down to baseline values (D). SAC *per se* did not modify the levels of RAGE in Sham-treated animals.

### Fluorometric measurements of *K*_*b*_

**Fig 7:**
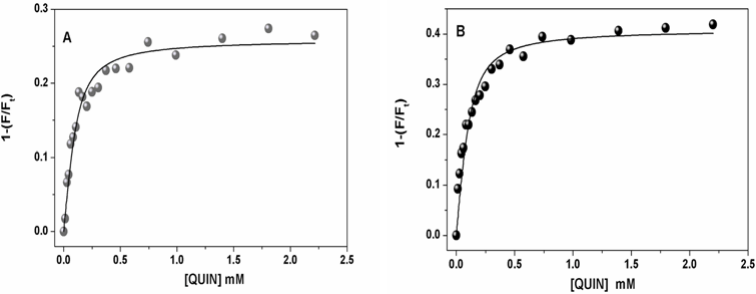
Titrations of VC1 with QUIN, monitored by fluorescence detection. The initial protein concentration was 0.1 μM. Excitation was 280 nm and fluorescence emission was measured at 320 nm. Circles represent the fraction of total VC1 fluorescence (F_t_) that is quenched by adding QUIN. Both titrations were done at 25°C in 20 mM Tris-base plus 133 mM NaCl at pH 7.4 (**A**) and in 20 mM glicine plus 133 mM NaCl at pH 9.0 (**B**). Data points were fitted to Equation (1) using nonlinear regression (solid line).

In order to validate the direct interaction between RAGE and QUIN suggested by the previous experiments, the binding constant, *K*_*b*_, of QUIN to VC1 from human sRAGE, was determined by fluorometric titrations. Representative binding isotherms at pH 7.4 and 9.0 are shown in Fig. 7, where the line represents the fitting of Equation 1 to the data, from which the dissociation constant, *K*_*d*_, was obtained. From these values, *K*_*b*_ was calculated from the equation *K*_*b*_ = 1/*K*_*d*_. The results obtained for both pH values were *K*_*b*_ = 2.3x10^7^ ± 0.03x10^7^ M^-1^ at pH 7.4, and *K*_*b*_ = 2.2 x10^7^ ± 0.1x10^7^ M^-1^ at pH 9.0. *K*_*b*_ did not change in the two pH conditions we tested. The affinity value obtained for QUIN-VC1 is considered as moderate, according to standards established by Velázquez-Campoy *et al* [58].

### Computational docking: Interaction between RAGE VC1 domains and QUIN

**Fig 8:**
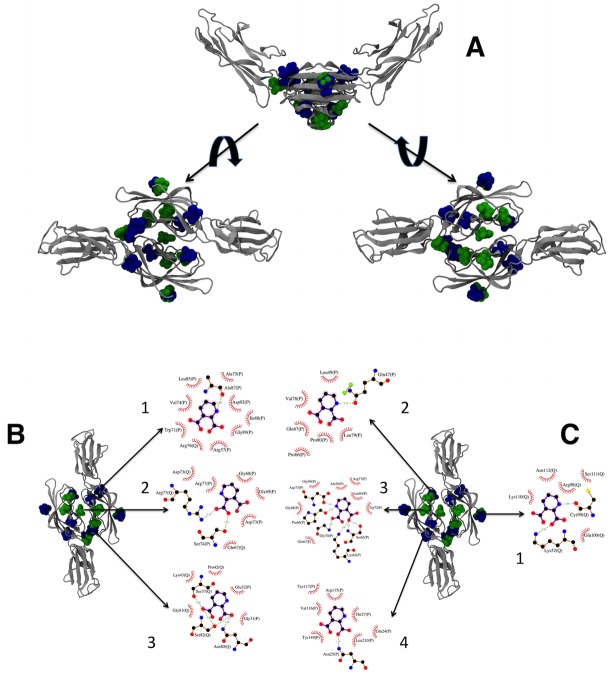
Docking for VC1-QUIN. In **A**, the structures of the 20 highest scoring conformers of QUIN obtained by docking on all surfaces of VC1 human (green) and rat (blue) RAGE domains. The topmost figure shows the V-shaped dimer in silver ribbons, with the QUIN molecules in spheres. The left structure shows the dimer from the top, and the right structure, from the bottom. In **B**, the three binding sites that involve residues from both monomers are shown (labels 1, 2, and 3), together with the details of the interacting residues for the best pose of each site using LIGPLOT+ [74] with the default parameters. In **C**, the four binding sites that only involve residues from one monomer are shown (labels 1 through 4), together with details of the interacting residues for the best pose of each site using LIGPLOT+ [74] with the default parameters. The 3D structures were prepared with VMD 1.9.1 [75]. In panels **B** and **C**, residue names and numbers are followed by the chain identifier (P or Q). Hydrogen bonds are indicated by green dashed lines with the distance between heavy atoms, and van der Waals contacts are indicated by red arcs with short lines. Residue numbers correspond to the full chain numbering from rat or human.

Docking of QUIN was performed on VC1 dimers from both human (derived from PDB-ID 4LP5 [32]) and rat RAGE (homology model obtained from I-TASSER [50,51]). QUIN was bound at five locations involving only one chain of the dimer, and at three locations that bridge both chains of the dimer, as shown in Fig. 8. We pooled the results from both docking assays, and grouped the poses according to the binding regions. These are shown in Fig. 8A. For detailed analysis of the interactions at each site, we chose the pose with the best binding energy and/or the most extensive protein contacts. These are shown in Fig. 8B and 8C.

## Discussion

In this work we explored a novel concept suggesting that QUIN-induced toxicity comprises the early triggering of RAGE toxic cascades and might also involve a direct chemical interaction with the RAGE protein. Besides its obvious interaction with the NMDAr [59] and its affinity for quinolinic acid phosphoribosyltransferase (QPRT) [60,61], its degradation enzyme, no other chemical interactions of QUIN with other proteins have been explored so far. The concept that QUIN and other redox metabolites from the KP might exert their actions through their binding and functional modulation of other proteins represents a novel alternative, and does not exclude other mechanisms to explain the many toxic features exerted by an aberrant metabolism of the KP in neurodegenerative disorders. Therefore, this study represents a first approach to explore this concept, providing experimental and computational evidence that the molecular complex conformed by QUIN-RAGE readily occurs. To our knowledge, crystallographic studies related with QUIN comprise mostly the molecular structure of the QUIN-QPRT complex [60]. However, the possibility that QUIN may interact with other proteins remains open, and would provide interesting explanations for the many toxic features already described for this metabolite [62] that cannot be merely explained considering its interaction with NMDAr.

RAGE is a pattern recognition receptor sensing endogenous stress signals. In turn, the activation of NF-ĸB is likely to be involved in chronic disorders, including diabetes, vascular complications, Alzheimer’s disease and cancer [32,63]. Short (transient) and long-lasting (sustained) activation of NF-ĸB due to RAGE binding of ligands such as AGEs, members of the S100 family, or amyloid-β peptide fibrils, are responsible for triggering inflammatory responses that are difficult to handle by cells. Thus, different RAGE ligands can activate the transmembrane form of the receptor, triggering the NF-ĸB pathway toward the degradation of repressor proteins, initiating toxic events, which lead further to chronic disorders [63] In this context, the process of RAGE oligomerization is key for the formation of signaling complexes. It has been recently proposed that multimodal RAGE oligomerization constitutes a mechanism by which ligand-specific complexes are formed, activating specific signaling functions [32]. In these events, transmembrane RAGE could be responsible for the activation of deadly cascades in neurodegenerative processes.

### QUIN infusion produced morphological alterations in the rat striatum remitted to the lesion site

In light of the relevance of RAGE for QUIN-induced neurotoxicity and degeneration, this study started with a morphological approach to those alterations produced by QUIN as a result of its infusion to the striatum. Initially, histopathological changes induced by QUIN were not expected to be extensive at 120 min after the lesion because this time is assumed to be too short to induce partial cell loss. Some articles in the literature have explored morphological alterations in the striatum of QUIN-infused rats some days following the lesion [57,64], but here, surprisingly, we were able to find moderate changes even at a short time. These lesions were characterized by vacuolization, edema, pyknosis, and changes in nuclear size, when compared against Sham. It was also evident, when comparing these images with those of unlesioned striata from the same animals, that the lesions were readily produced after the action of the toxin, and not only by mechanical means (see Fig. 2). The explanation for this early effect of QUIN can be summarized in two major points: 1) the early and significant expression of toxic markers (inflammatory molecules, reactive species, oxidative damage and RAGE expression, accompanied by down-regulation of neuroprotective genes) that prompted a more intense histological lesion that has already been demonstrated for a QUIN-induced striatal lesion under the same experimental conditions [11]; and 2) the limited zone of the lesion produced by QUIN, that was just circumscribed to a nearby area surrounding the injection site. In this regard, it is true that a mechanical lesion can induce degenerative and inflammatory processes itself, but when comparing QUIN-lesioned *versus* Sham striata, the very nature of the lesion induced by QUIN exhibited different morphological components that were not present in the latter, hence suggesting that specific toxic mechanisms involved in neurodegeneration are already in progress since the very beginning of the toxicant infusion, and will spread in time to affect the whole region. From this point on, it seems plausible to consider that QUIN toxicity starts exerting early morphological changes from the very beginning of its infusion, implying that these criteria should be contemplated for further studies using the same experimental conditions.

### QUIN did not decrease the striatal neuronal density at short times post-lesion

Congruent with the early morphological alterations induced by QUIN at 120 min post-lesion (described above), the toxicant caused only a slight decrease in the neuronal density at this time, although the decrease resulted non-significant and revealed what we can interpret as an early and moderate tendency to initiate the neurodegenerative process. Therefore, this finding suggests a degenerative role for this neurotoxin in progress since the very beginning of its infusion to the cerebral tissue (see Fig. 3), although the fact that the reduction in neuronal density produced by QUIN at 120 min was limited to 13% is revealing that the lesion is limited, but once again, readily in progress. Previous times of exposure to QUIN did not show significant changes of this endpoint. In addition, this loss of neuronal density observed at 120 min post-lesion was localized only nearby the injection/infusion site. Noteworthy, at the finest level, surviving neuronal cells from the QUIN-lesioned striata and surrounding the lesion site exhibited moderate volume loss and intracytoplasmic vacuolization, which suggests that toxic mechanisms elicited by QUIN are modifying the cellular architecture and prompting cells to die. Previous studies using NeuN immunolabeling in QUIN-lesioned brains support the ability of this toxin to induce damage specifically on neuronal cells [65,66].

### The QUIN-induced early striatal alterations matches with RAGE expression

A previous study demonstrated that an enhanced RAGE expression is readily occurring in the toxic model induced by QUIN at 120 min post-lesion [11]. In the present work, we confirmed this finding using immunohistochemical approaches (see Fig. 4), thus supporting the concept that RAGE is up-regulated in response to the toxic environment imposed by QUIN, which in turn may include excitotoxic, inflammatory and pro-oxidant conditions. Striatal cells were extensively immunolabeled for intracellular RAGE, but the nature of this response is still difficult to establish simply by an immunostaining assay. This RAGE response might involve its anchoring and further oligomerization to trigger a more prominent inflammatory response, as judged by the results of Cuevas *et al*. [11], in which RAGE expression elicited under the same experimental conditions employed here, was accompanied by NF-ĸB up-regulation, NO formation and some toxic markers.

Supporting evidence for the stimulatory role of QUIN on RAGE expression was achieved with immunofluorescence and Western blot assessments (see Figs. 5 and 6, respectively). In this work, the band assigned to full-length RAGE (including the intracellular domain) in the Western blot was identified at ~50 kDa, and this band corresponds to the RAGE protein form that has been identified for human and mouse using the same antibody appearing in a previous report at around 55 kDA [67]. RAGE is therefore a protein migrating at different molecular weights, depending on its forms, and an explanation for its differential migration pattern in Western blot analysis among different studies can related with several factors, including its heterogeneous glycosylation [68], the biological preparations that were analyzed, types of gels and general conditions for running, etc.

QUIN stimulated a significantly positive fluorescent signal for RAGE, again at 120 min, demonstrating that the toxic insult is sufficiently intense to elicit an up-regulation of this receptor. Our findings on immunofluorescence also revealed that RAGE expression is taking place in neuronal cells and striatal fibers. In addition, we were able to demonstrate not only that RAGE expression is stimulated by QUIN, but also that this early effect is dependent on pro-oxidant conditions. The fact that S-allylcysteine (SAC), a well-described antioxidant [69], is capable of reducing RAGE content in QUIN-treated animals, clearly suggests that reducing the presence of ROS in the toxic environment constitutes a useful strategy to limit RAGE up-regulation. This evidence supports a causative role of oxidative stress in the toxic pattern exerted by QUIN, and prompts the design of antioxidant-based therapies against neurodegenerative events involving oxidative/excitotoxic/inflammatory components. Other groups have raised the relevance on the redox environment on the RAGE regulation, supporting the concept that this receptor functionally responds to oxidative conditions to favor different scenarios [70]. Up to this point, we have demonstrated, by different methodological means, that QUIN is able to induce RAGE up-regulation, and this effect can account for QUIN toxicity, leading to triggering inflammatory and degenerative pathways, as established in a previous report with the same model [11]. QUIN-induced RAGE up-regulation could be elicited in turn by several components of QUIN toxicity [10], including the intra- and extracellular action of ROS/reactive nitrogen species (RNS) to further increase AGEs and other RAGE ligands, as well as other major toxic events such as excitotoxicity, inflammation itself and mitochondrial dysfunction. How these events could be acting separately or in a concerted manner to induce RAGE up-regulation in this model remains to be elucidated in further studies. For further consideration, given that cell death processes started within 120 min, it is possible that QUIN is activating alternative toxic mechanisms, including a direct interaction with RAGE, hence promoting signaling cascades that do not involve transcription. Therefore, it becomes essential to document the direct RAGE-QUIN interaction. In the meantime, our results support the concept that RAGE-dependent toxic activation is being initiated simultaneously to a QUIN-dependent degenerative process already in progress.

### QUIN showed affinity for the VC1 RAGE domain: *in vitro* and computational studies

The fluorometric studies indicated that affinity of QUIN for the RAGE domains is moderate (on the order of 100 nM), comparable to that of AGEs with RAGE [36]. This method was used for the VC1 domain as it contains various tryptophan and tyrosine residues that can serve as binding monitors over the surface of the protein. In this regard, another important issue is RAGE oligomerization. Koch *et al*. [27] reported that VC1 oligomerization is favored in *in vitro* assays carried out at pH 7.6. Based on the results of our experiments, this tendency was confirmed at pH 7.4 by protein precipitation; however, at pH 9.0 RAGE showed more stability, according to previous studies also conducted at pH 9.0 [39,71,72]. We therefore measured QUIN binding at both pH values, finding that the binding constant for the QUIN-RAGE complex was similar at pH 7.4 and 9.0. This is expected, as QUIN is a diprotic molecule presenting negative charge at the tested pH values.

In our docking experiments of QUIN to the VC1 dimers of human and rat RAGE, we found seven different binding sites that are adjacent to fluorescent residues, and would therefore correspond to the fluorometric assay described above (see Fig. 8A). Four of the binding sites involve only one monomer (Fig. 8C), and among these we found the site where two small AGEs bound to the isolated V domain [36,39] (structure 1 in panel 8C; all the residues involved in QUIN binding correspond to those identified in the interactions with AGE-modified lysines and arginines). Contrary to the multivalent nature of AGE-modified proteins or other large RAGE ligands, which can promote direct oligomerization, signaling through these binding events to a RAGE monomer would require strong allosteric effects on RAGE, which would need to travel to the membrane domain to exert a change in RAGE oligomerization. On a more interesting note, the fact that QUIN can occupy the same site as AGE-modified proteins may suggest either a competition or a potentiation of effects between these ligands and QUIN, adding to the complexity of RAGE signaling in an inflammatory setting. The remaining three binding sites involve both monomers in the interaction with QUIN (Fig. 8B), and would therefore promote dimerization. All of these sites are accessible from the solvent. One of these involves both V-V and V-C1 interactions (structure 3 in panel 8B), and overlaps with the binding site of nucleic acids [35] and heparin [34]. This again hints at the possible interplay between the extracellular matrix and QUIN binding, leading to tissue degeneration. Of further consideration is the suggestion that small ligands, like QUIN, might play a role in the organization of the V and VC1 domains. In addition, the binding of ligands of different sizes or shapes may lead to hinge adjustments and enforce charges in the C2 domain and intracellular domains necessary for ligand-dependent signaling [73]. Nonetheless, the precise role of the QUIN binding to RAGE remains unknown in terms of its physiological and/or physiopathological functions.

## Conclusion

The present study confirmed previous findings of our group on the possible involvement of RAGE in QUIN-induced *in vivo* neurotoxicity. Although RAGE is commonly associated with pro-inflammatory responses that provoke neurodegeneration, the precise mechanisms involved in those pathways triggered by the interaction of the receptor and its ligands remain unsolved. It is clear now that QUIN toxicity recruits RAGE for the induction of early and late striatal damage; however, for the first time we suggest that the interaction of these molecules could be also occurring at the molecular level. Then, the possible physical interaction of QUIN in the dimer could be relevant for the initiation of early signaling cascades associated to triggering NF-ĸB pathway, and would involve crosstalk with other known RAGE ligands. Of course, this interaction will require further *in vivo* demonstration, but in the meantime, it represents an alternative explanation for the toxic pattern exerted by QUIN. Therefore, this work correlates the computational models with *in vitro* experiments, and thus it contributes to the understanding of the possible phenomenon of RAGE-QUIN recognition. The docking models provide a qualitative understanding of plausible molecular mechanism for the signaling of QUIN to proteins like RAGE, thereby allowing the consideration of anti-RAGE therapies for neurodegenerative disorders coursing with the enhanced presence of endogenous neurotoxins.
