# Expression of Concern: Modeling the Interaction between Quinolinate and the Receptor for Advanced Glycation End Products (RAGE): Relevance for Early Neuropathological Processes

**DOI:** 10.1371/journal.pone.0281905

**Published:** 2023-02-14

**Authors:** The PLOS ONE Editors

Following the publication of this article [1], concerns were raised regarding results presented in Figs 3 and 6. Specifically,

- The Fig 3D panel appears similar to the Fig 3E panel, despite being used to represent different experimental conditions.
- In Figs 6A, 6B, and 6C, there appear to be vertical irregularities between the SAC+QUIN (lane 3) and the Sham+SAC (lane 4) results, suggestive of splice lines.

The corresponding author stated that the Fig 3D panel was inadvertently duplicated during figure preparation and incorrectly used to represent the Fig 3E results. The updated Fig 3, available with this notice, presents the correct Fig 3 results, and the underlying data for Fig 3G are provided in S2 File below. As the error occurred during figure preparation, the results presented in Fig 3G were not affected.

The authors confirm that the blots presented in Fig 6 were prepared using spliced blots. They explain that the Sham+SAC panels were run on separate blots at a later time because there were insufficient lanes to include the control on the same blot, and insufficient antibody to develop multiple separate blots at the same time. The authors submitted the original underlying blots used to present the Fig 6 panels, which are provided in S6 File below.

Fig 6 and the underlying data provided by the authors were reviewed by a *PLOS ONE* Editorial Board member, who commented that the Sham+SAC control is an essential control for the Fig 6 experiments, and emphasized that this control needs to be included in the same experiment and on the same blots as the Sham, QUIN, and SAC+QUIN samples to serve as a valid internal control. As reported in the published article, i.e. with the Sham+SAC results run on a separate blot, the results presented in Fig 6 are not adequately supported.

In addition, the board member commented that the reduction of RAGE expression by SAC is not sufficient to support conclusions about the role of oxidative stress in response to QUIN. To support such a statement, it would be important to perform additional assays that demonstrate whether QUIN increased oxidative stress-related parameters. The authors stated that they have previously conducted not only these additional assays, but also experiments using an analogous toxic paradigm demonstrating the antioxidant efficacy of SAC. These results have been published in articles referenced below [76–85]. In light of these new references, the following text is added between sentences 5 and 6 of the third paragraph of the Discussion subsection **The QUIN-induced early striatal alterations matches with RAGE expression**: “In this regard, our group has been previously able to demonstrate both the active role of QUIN in promoting oxidative damage in the CNS [76–83], as well as the protective effects exerted by SAC as an antioxidant compound on several endpoints of the QUIN-induced oxidative stress [84, 85], thus supporting our current conclusions.”

The *PLOS ONE* Editors issue this Expression of Concern to notify readers of the concerns with Fig 6, and to relay the updated Fig 3 and the supporting data provided by the corresponding author.

**Fig 3:**
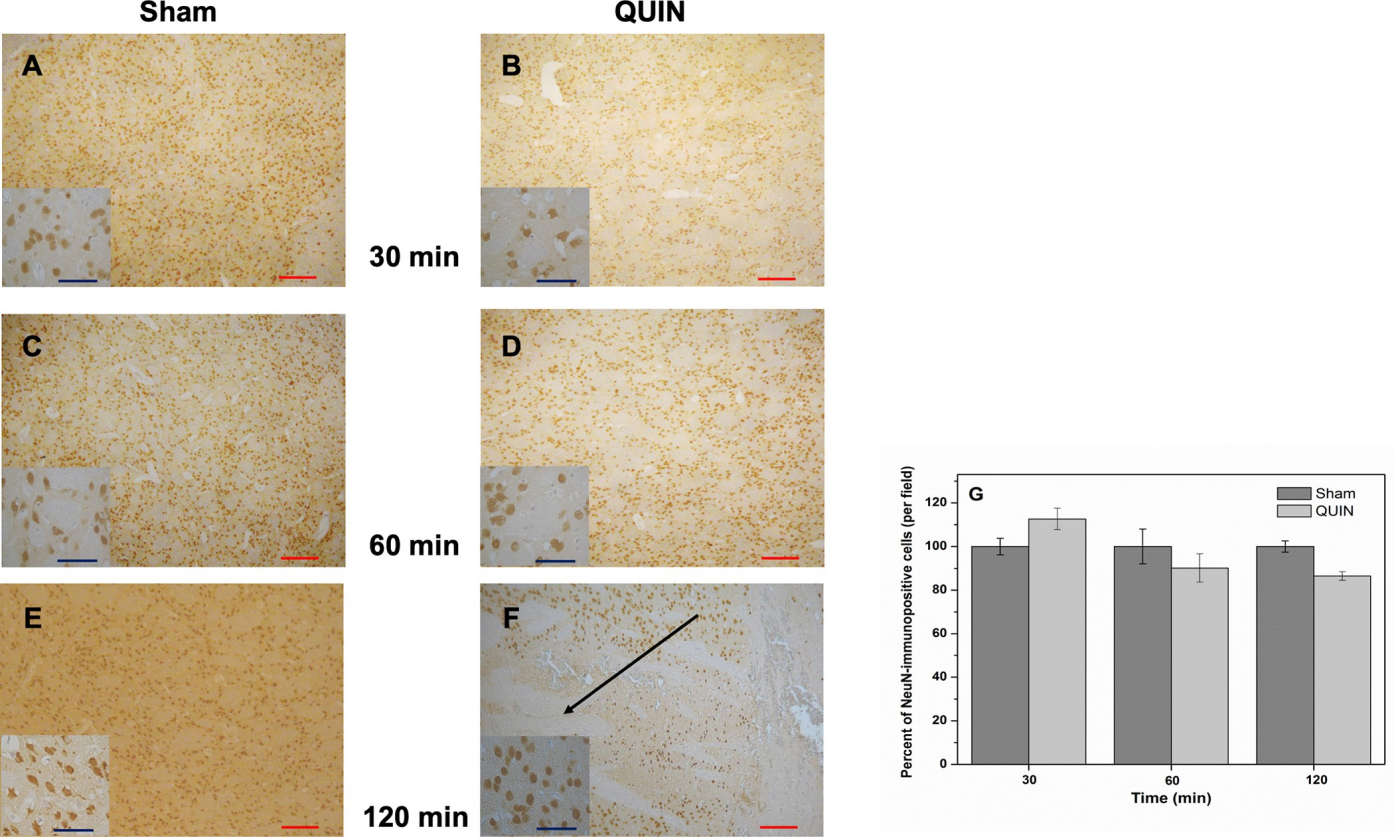
Histochemical alterations produced by QUIN in rats. Peroxidase-based immunohistochemical staining of neuronal cells (NeuN) in striatal coronal sections (10X) of Sham (**A**, **C** and **E**)- and QUIN (**B**, **D** and **F**)- treated animals at different post-lesion times (Bar size 100 μm). Details of cell morphology for each treatment are shown in small squares (40X). The segmentation method was employed for cell counting, and expressed as immunopositive cells. In **A**, **C** and **E**, normal appearance of the striata with normal cell densities are shown. In **B**, **D** and **F**, the striatal appearance at 30, 60 and 120 min post-lesion is presented. Also in **F**, a considerable loss of neuronal density (indicated by arrow) can be appreciated close to the lesion site. In **G**, the numbers of immunopositive cells (mean percent ± SD), determined by the segmentation method, are graphically represented.

## Supporting information

S1 File Original data underlying the Fig 2 results. (DOCX)

S2 File Original data underlying the Fig 3 results. (DOCX)

S3 File Supporting material for Fig 4 Histochemical labelling. (PPT)

S4 File Additional supporting material for Figs 3 and 4. (PPT)

S5 File Original data underlying the Fig 5 results. (DOCX)

S6 File Original data underlying the Fig 6 results. (DOCX)

S7 File Original data underlying the Fig 7 results. (PDF)
