# Supplementary material for: Expression of Concern: Modeling the Interaction between Quinolinate and the Receptor for Advanced Glycation End Products (RAGE): Relevance for Early Neuropathological Processes
Source: PLoS One. 2023 Feb 14;18(2):e0281905. doi: 10.1371/journal.pone.0281905 (PMC9928092; doi:10.1371/journal.pone.0281905)

**S1 File: Original data underlying results in Figure 2**

**
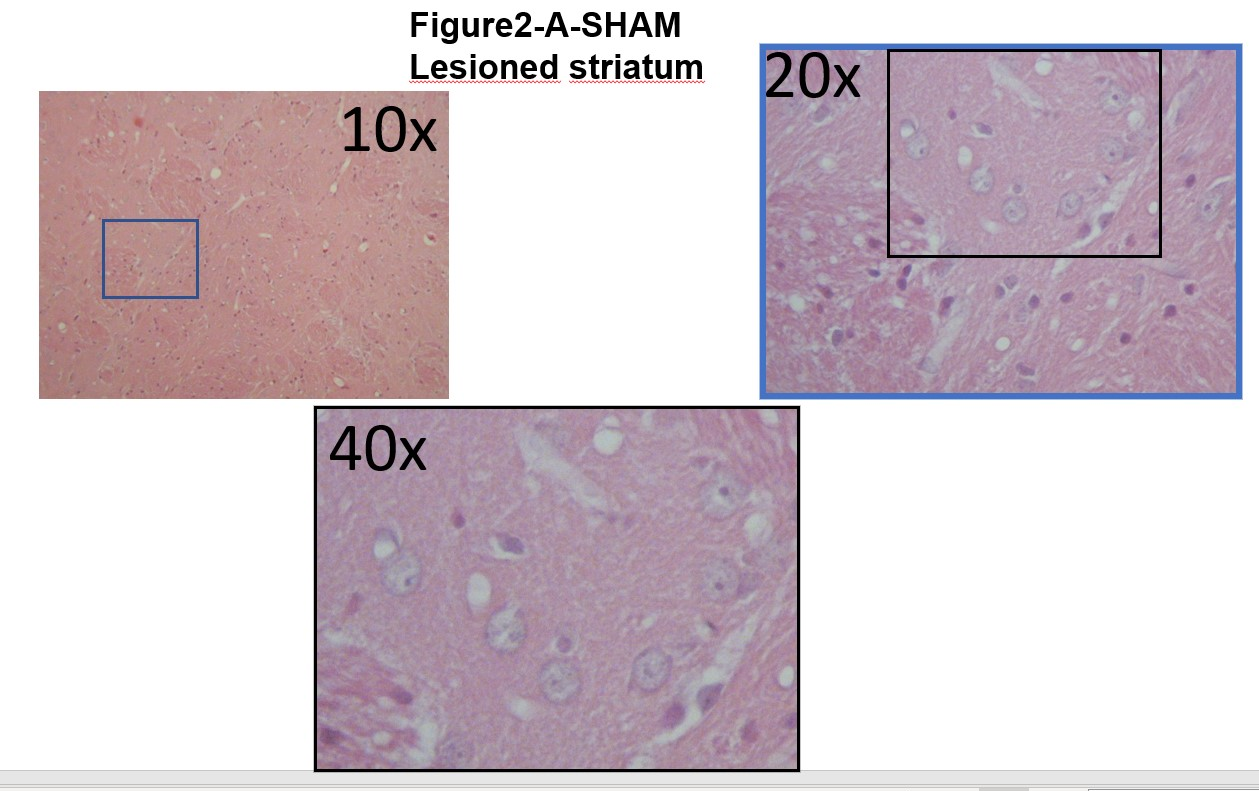
**

**
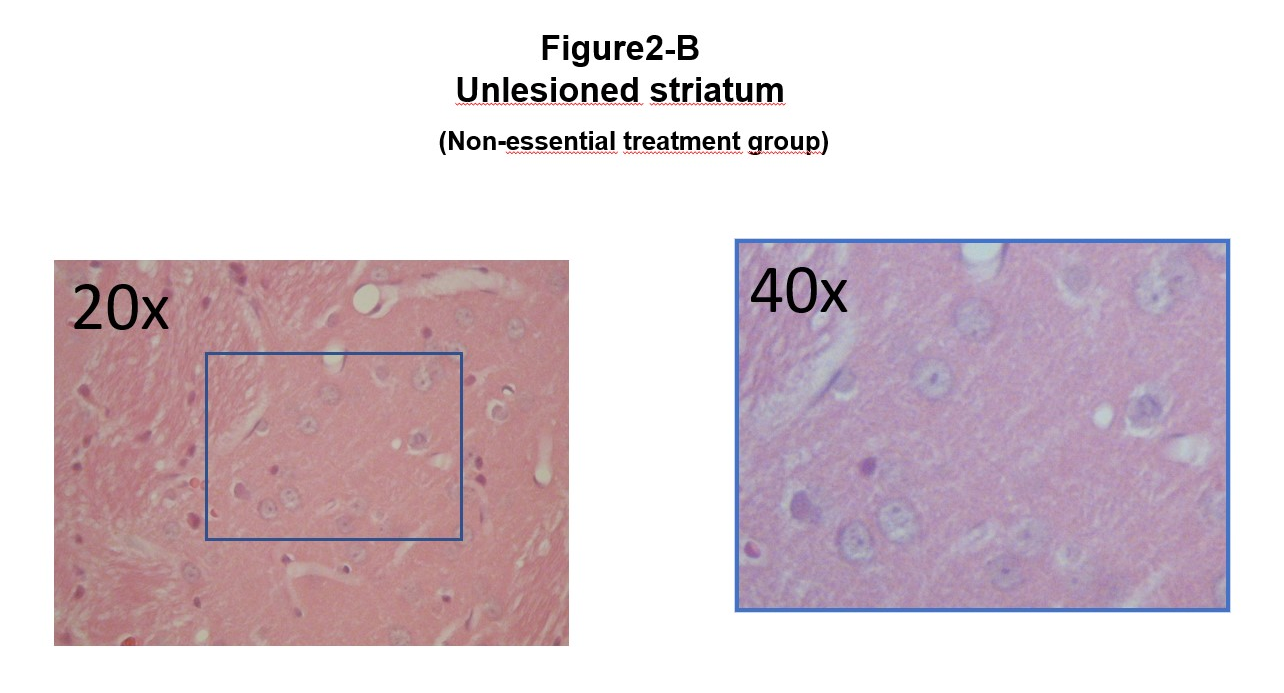
**

**
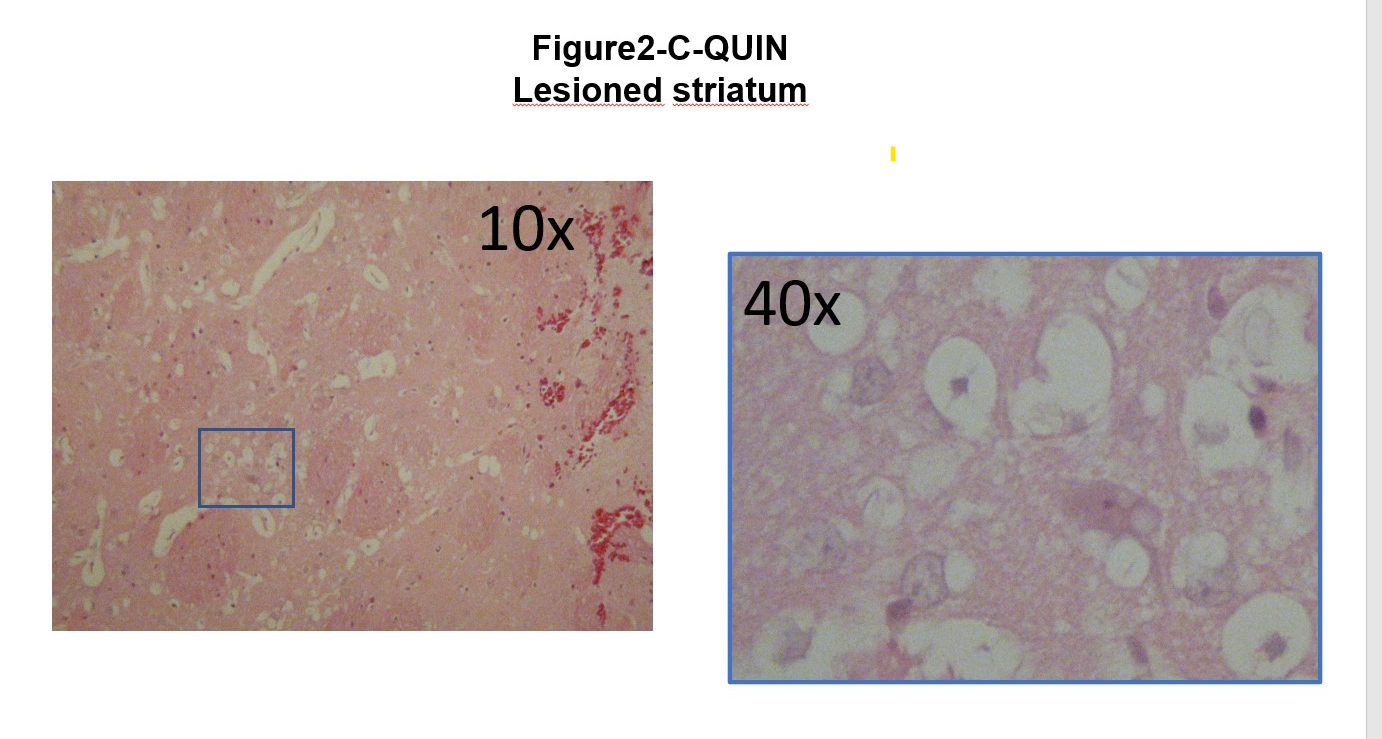
**

**
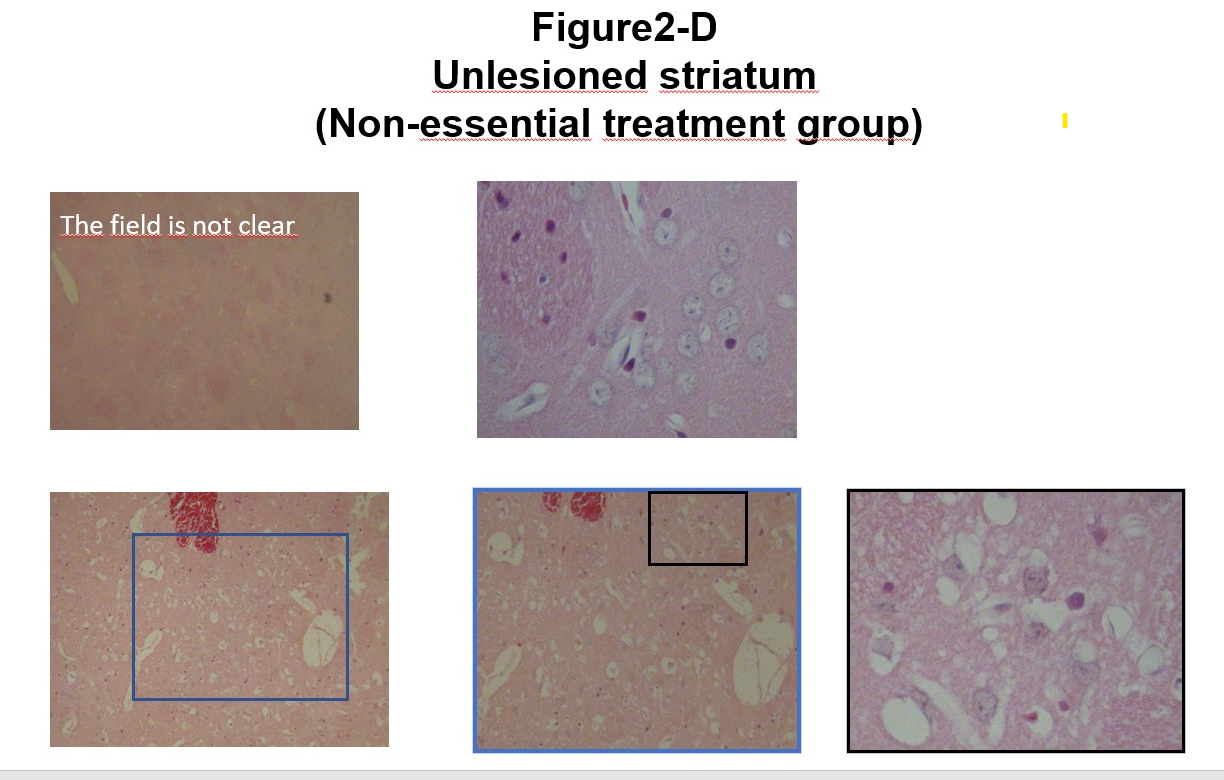
**

**Supporting material (not used for the figure) for Sham and QUIN 120 min post-lesion (for demonstrative purposes)**


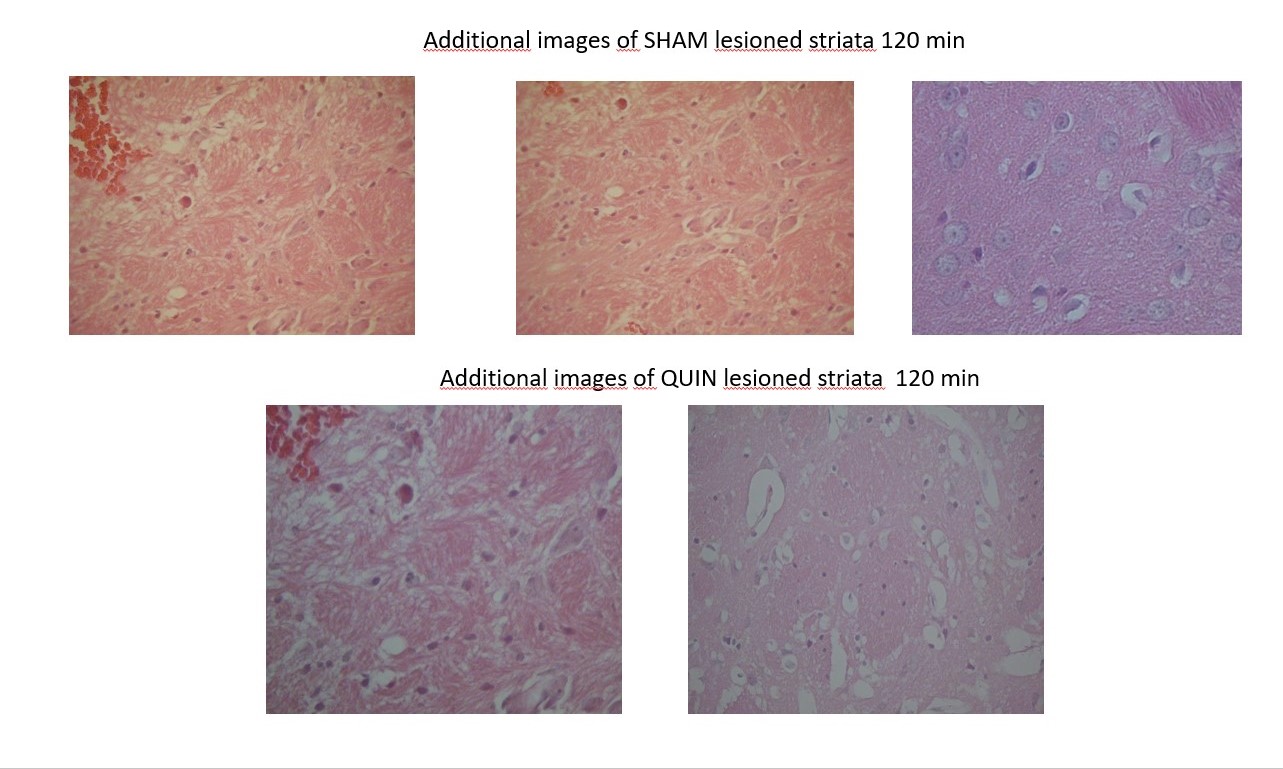

Supplement: S1 File — (DOCX) [file pone.0281905.s001.docx]
