# Supplementary figures and images for: Expression of Concern: Modeling the Interaction between Quinolinate and the Receptor for Advanced Glycation End Products (RAGE): Relevance for Early Neuropathological Processes
Source: PLoS One. 2023 Feb 14;18(2):e0281905. doi: 10.1371/journal.pone.0281905 (PMC9928092; doi:10.1371/journal.pone.0281905)

**S2 File: Original data underlying results of Figure 3**

**
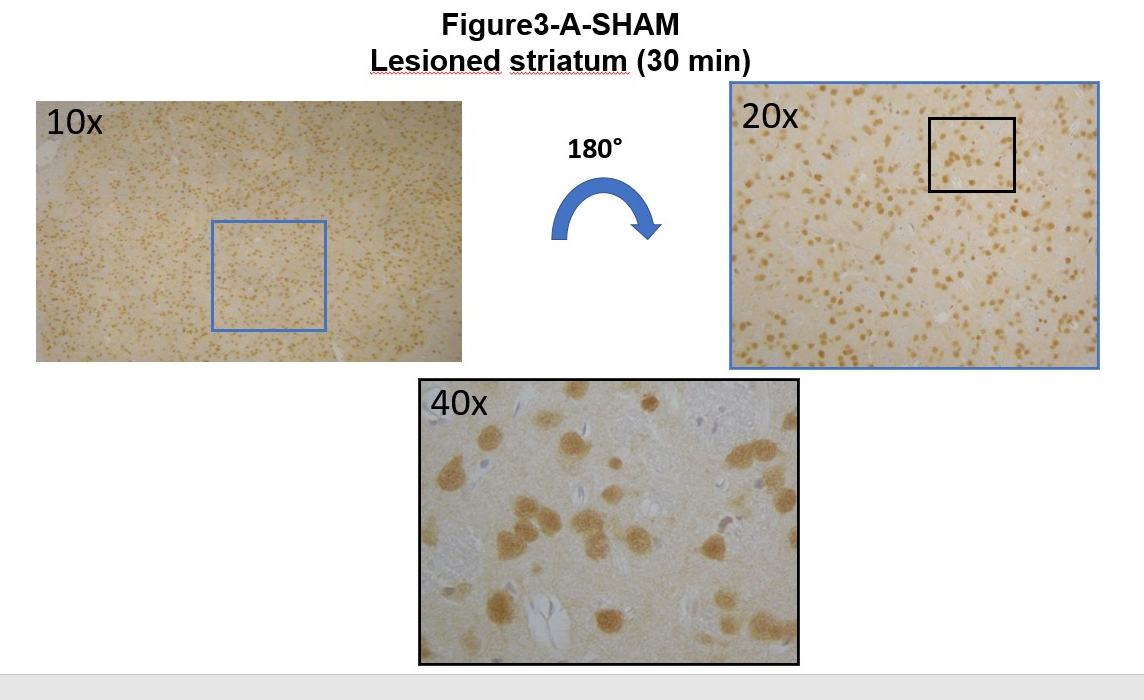
**

**
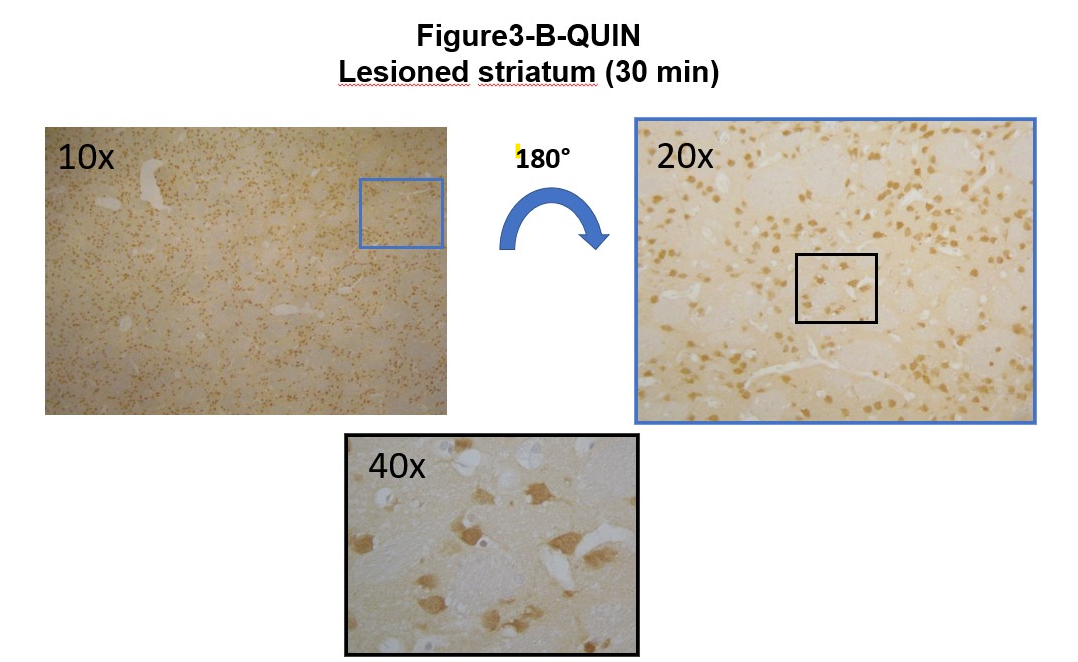
**

**
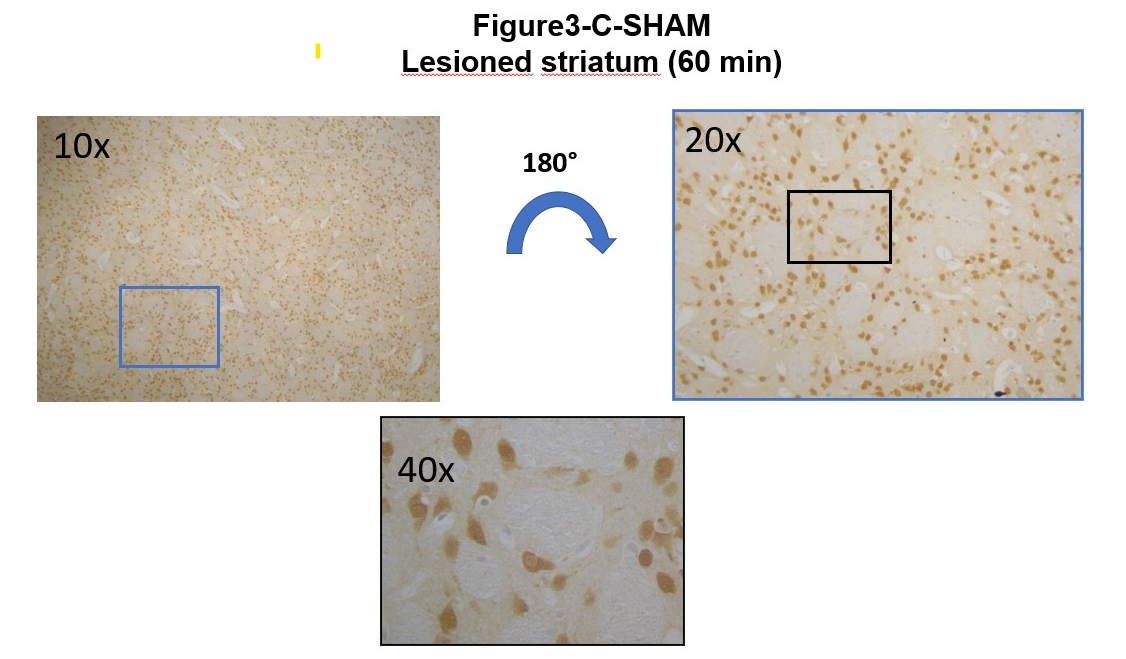
**

**
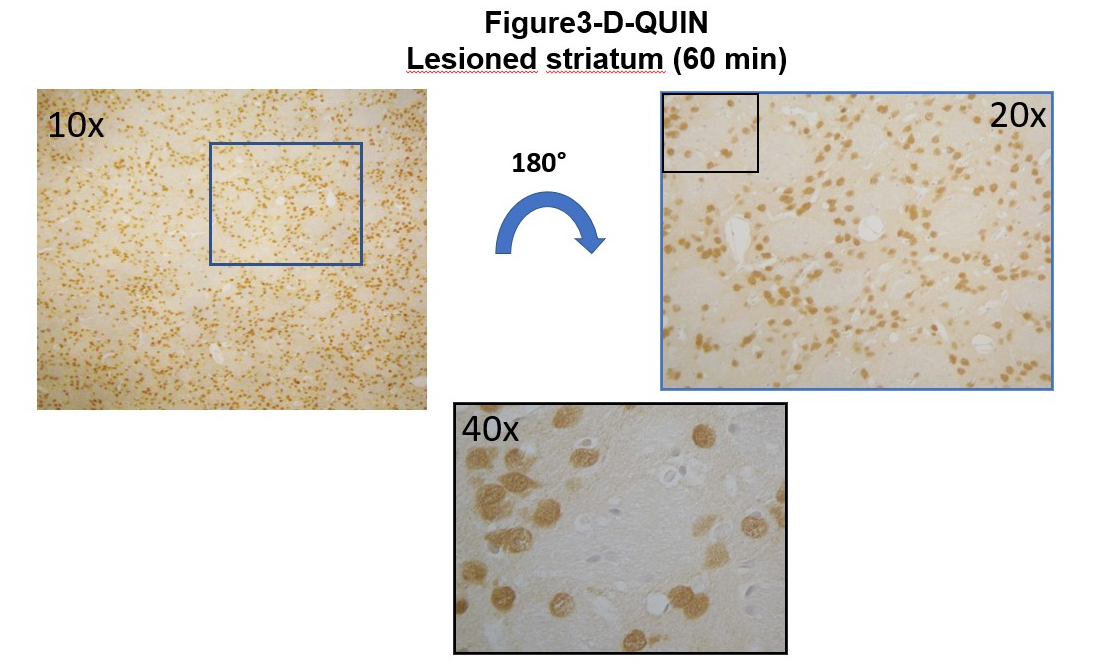
**

**
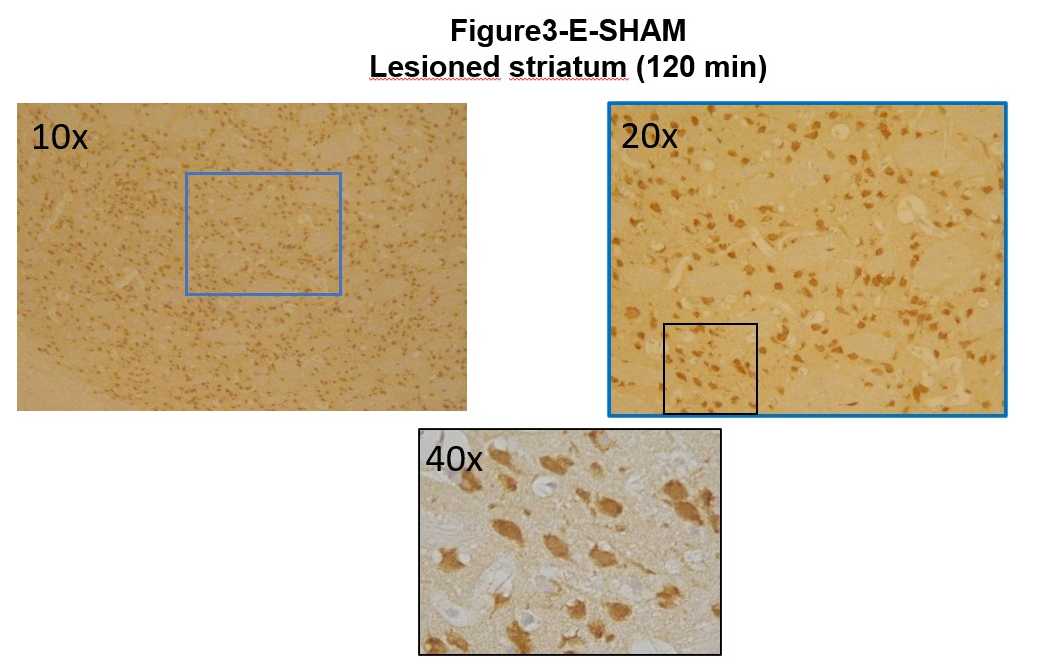
**

**
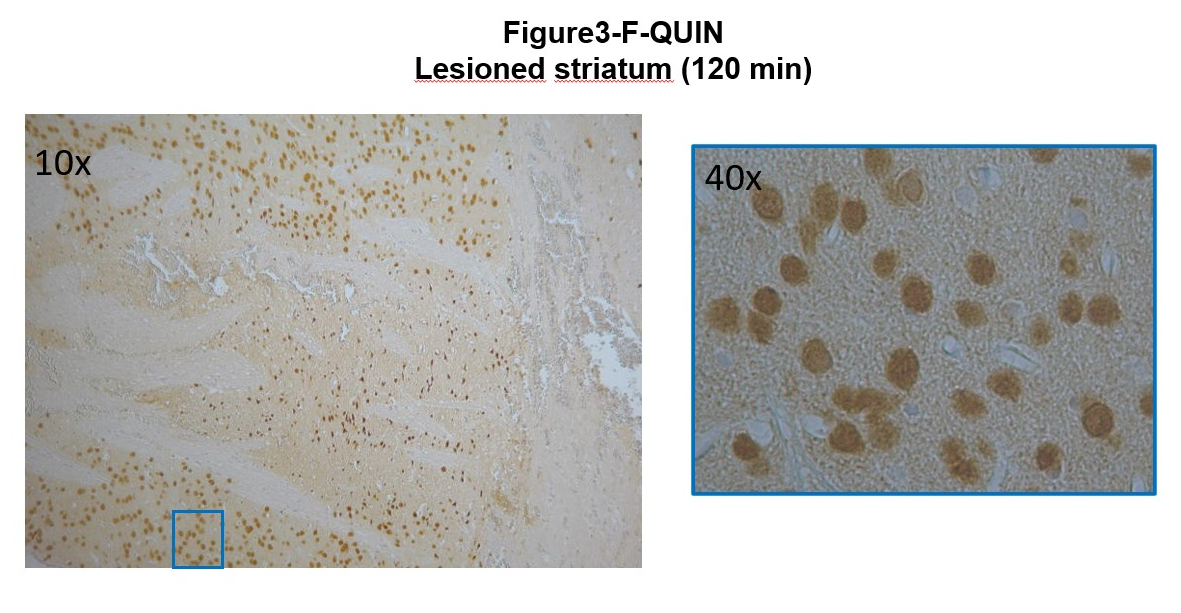
**

**
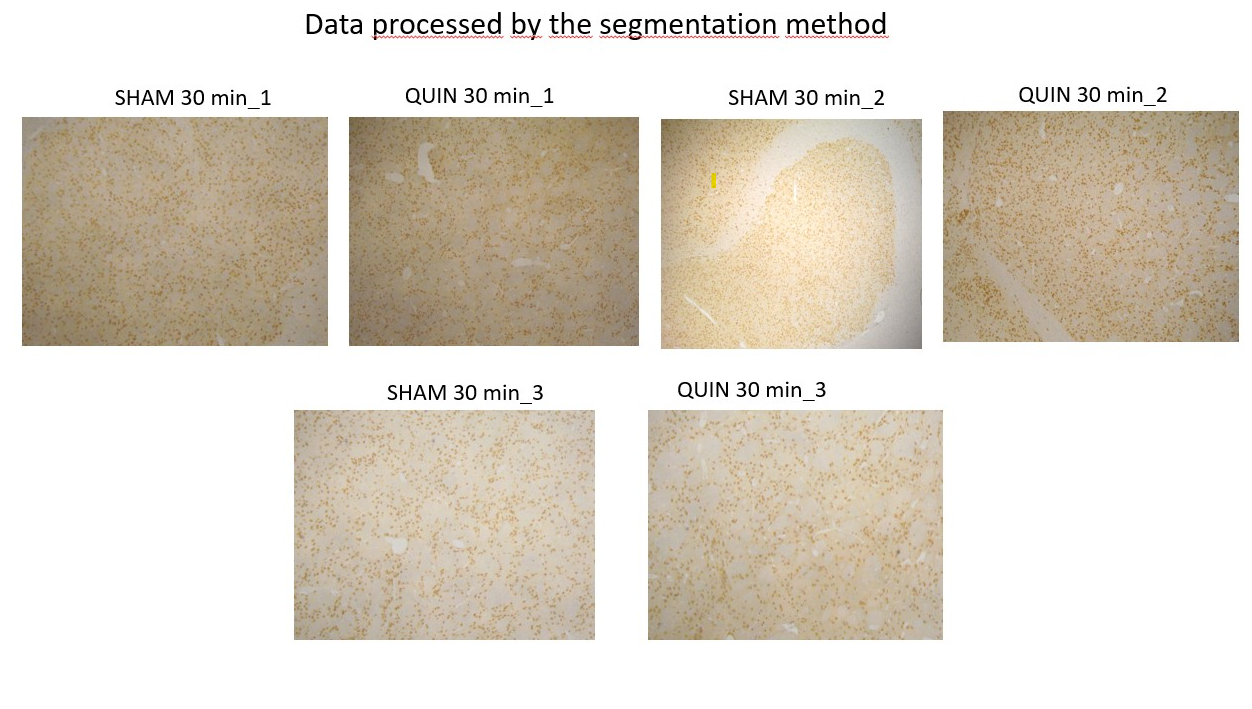
**

**
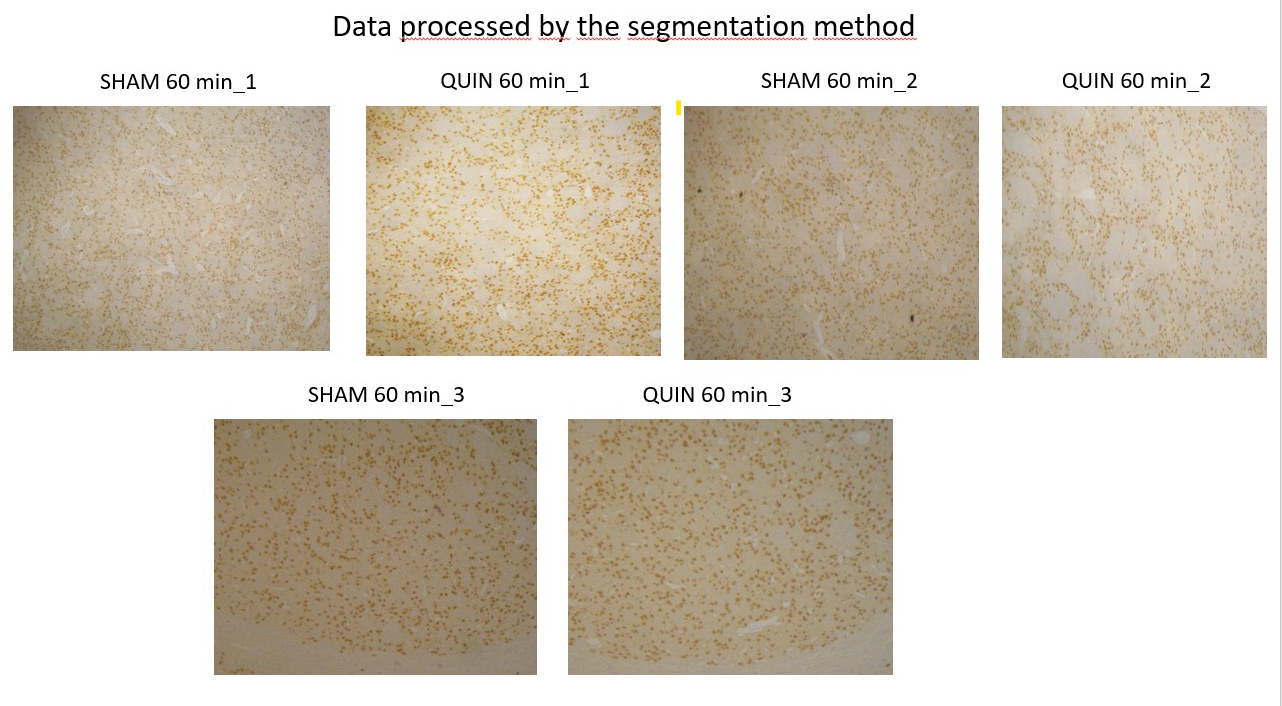
**

**
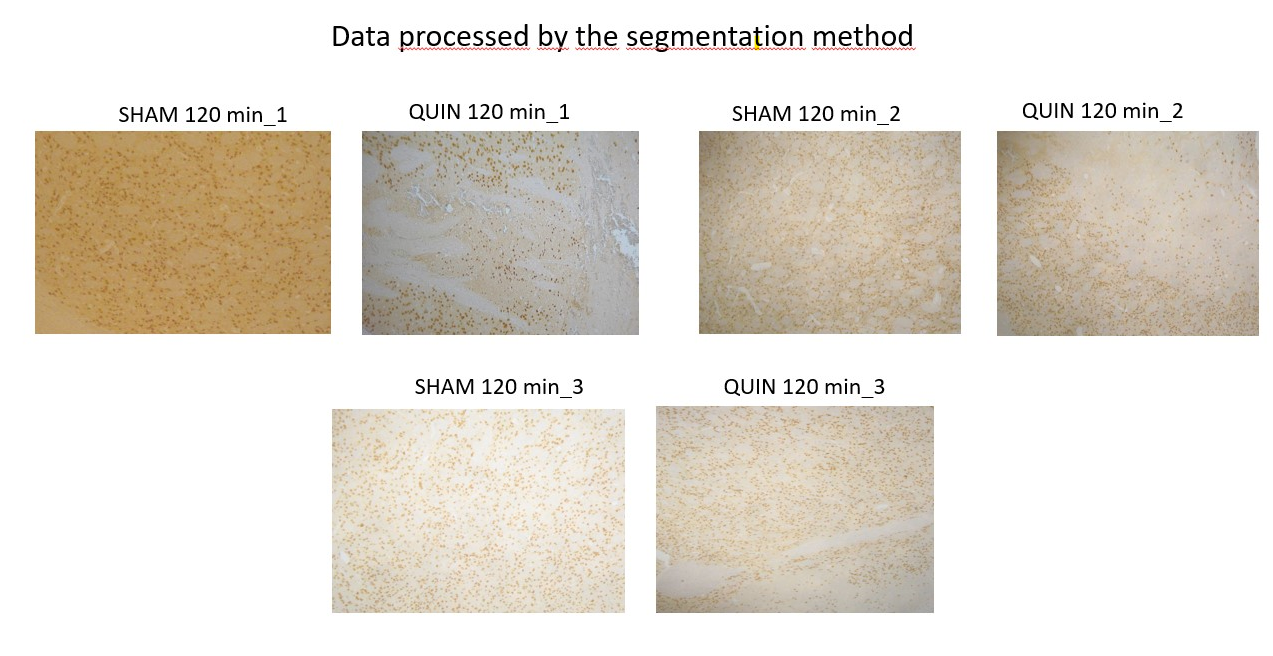
**


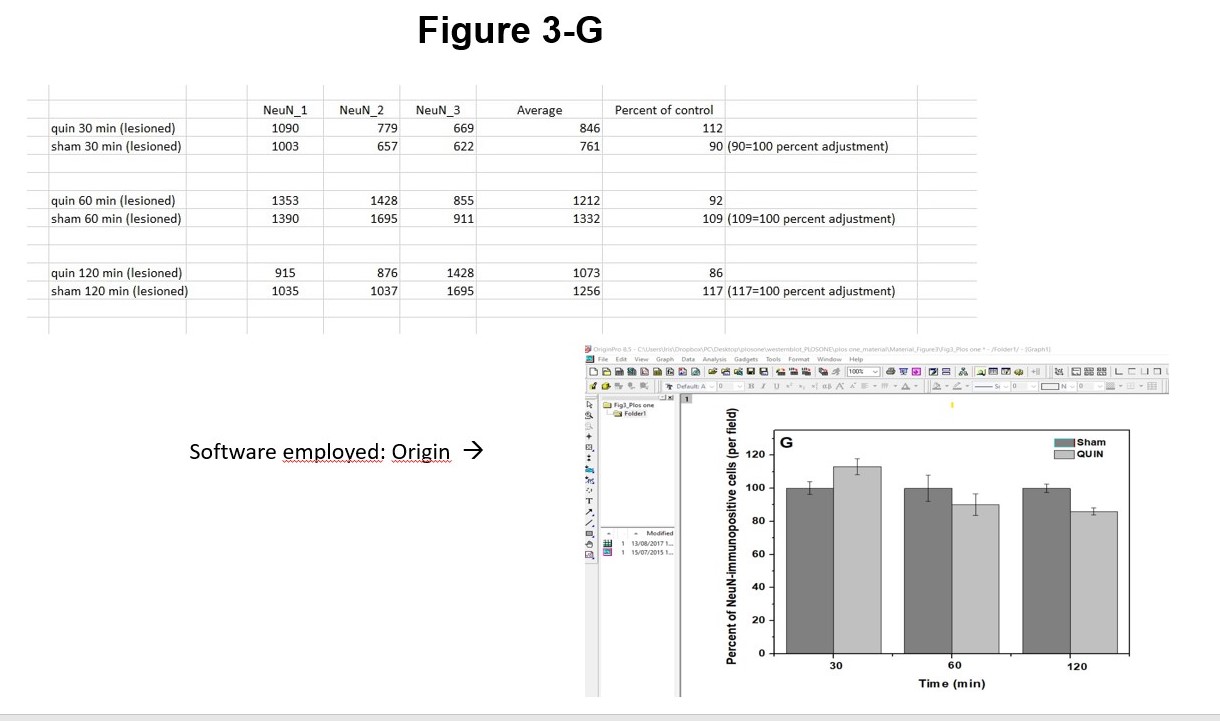

Supplement: S2 File — (DOCX) [file pone.0281905.s002.docx]

**S6 File: Original data underlying results of Figure 6**

**
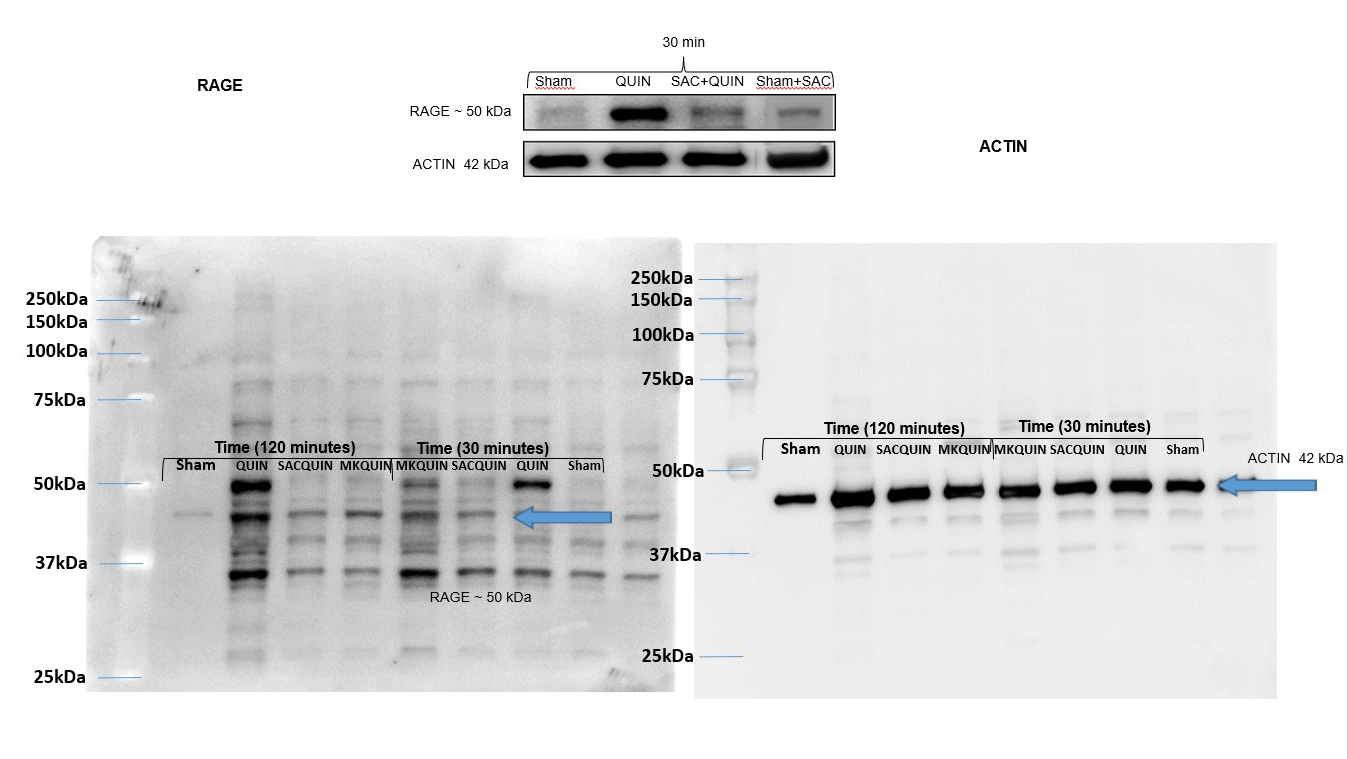
**

**
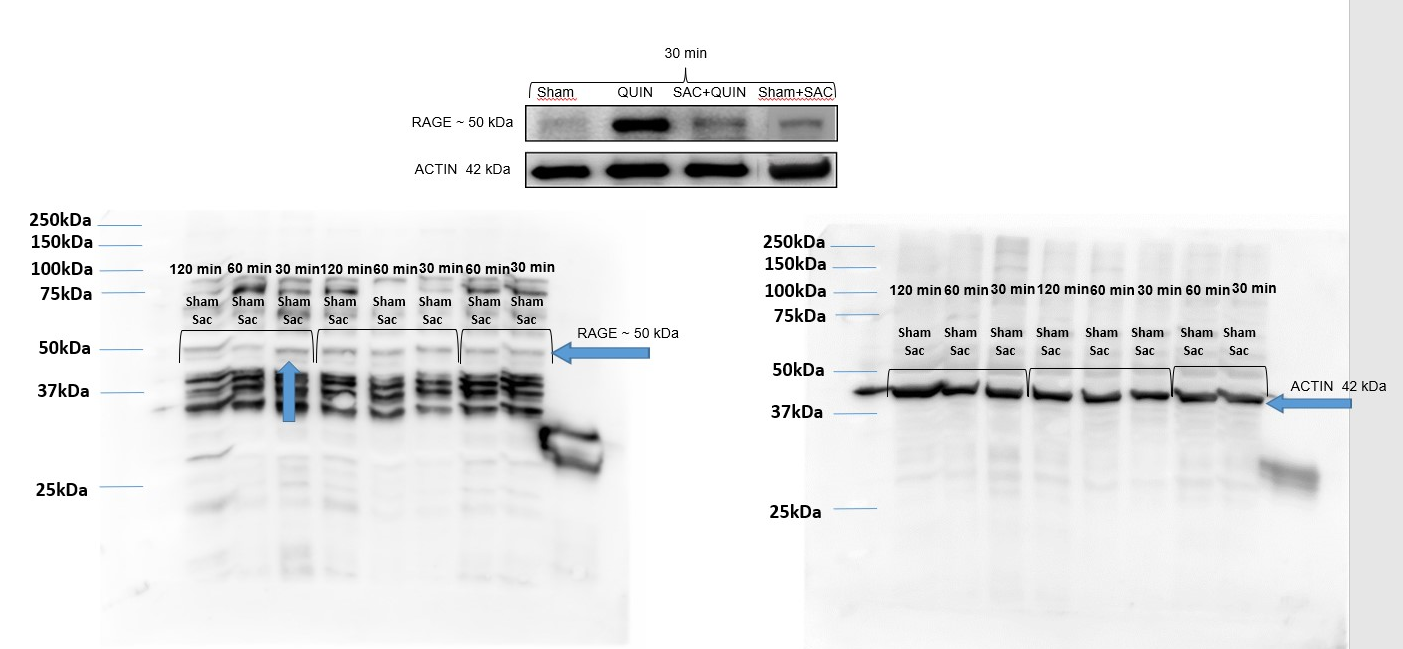
**

**
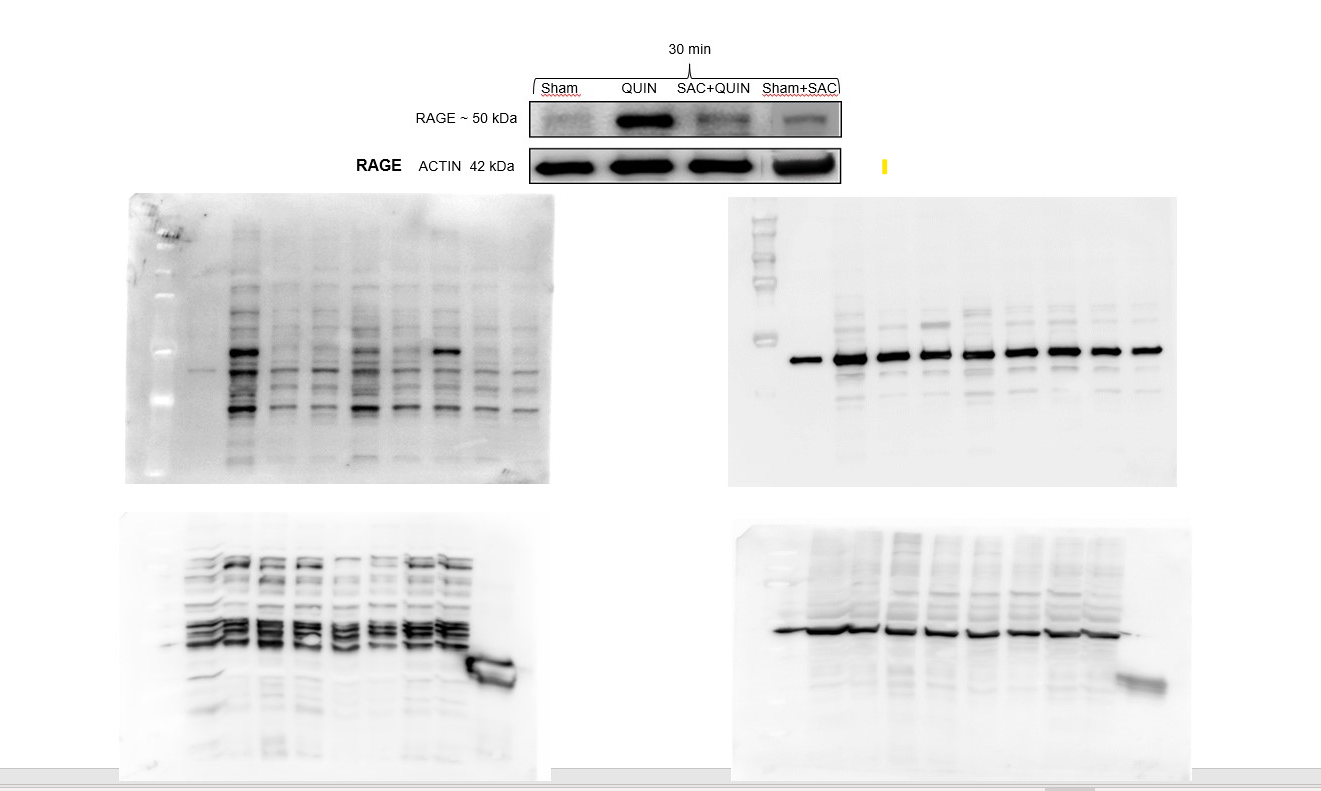
**

**
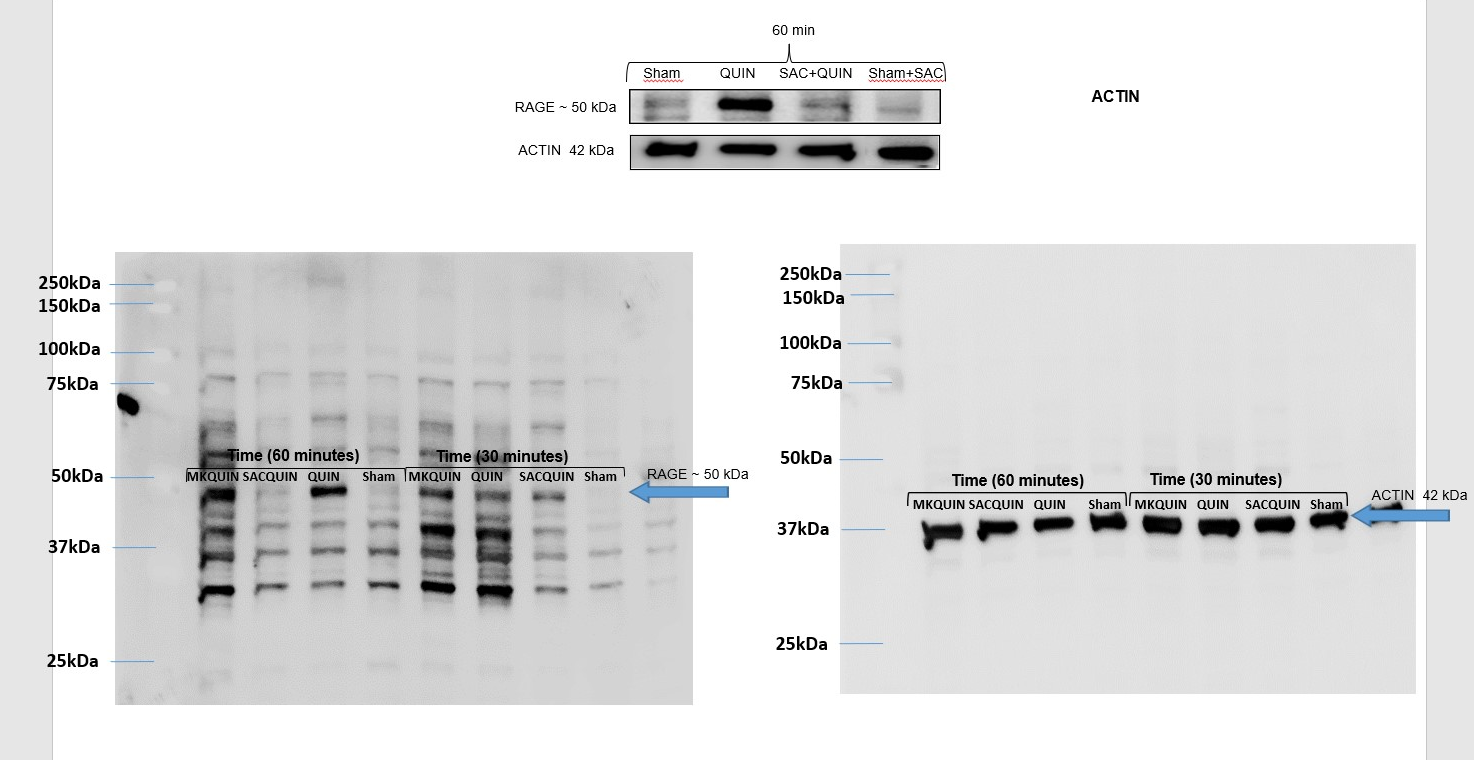
**

**
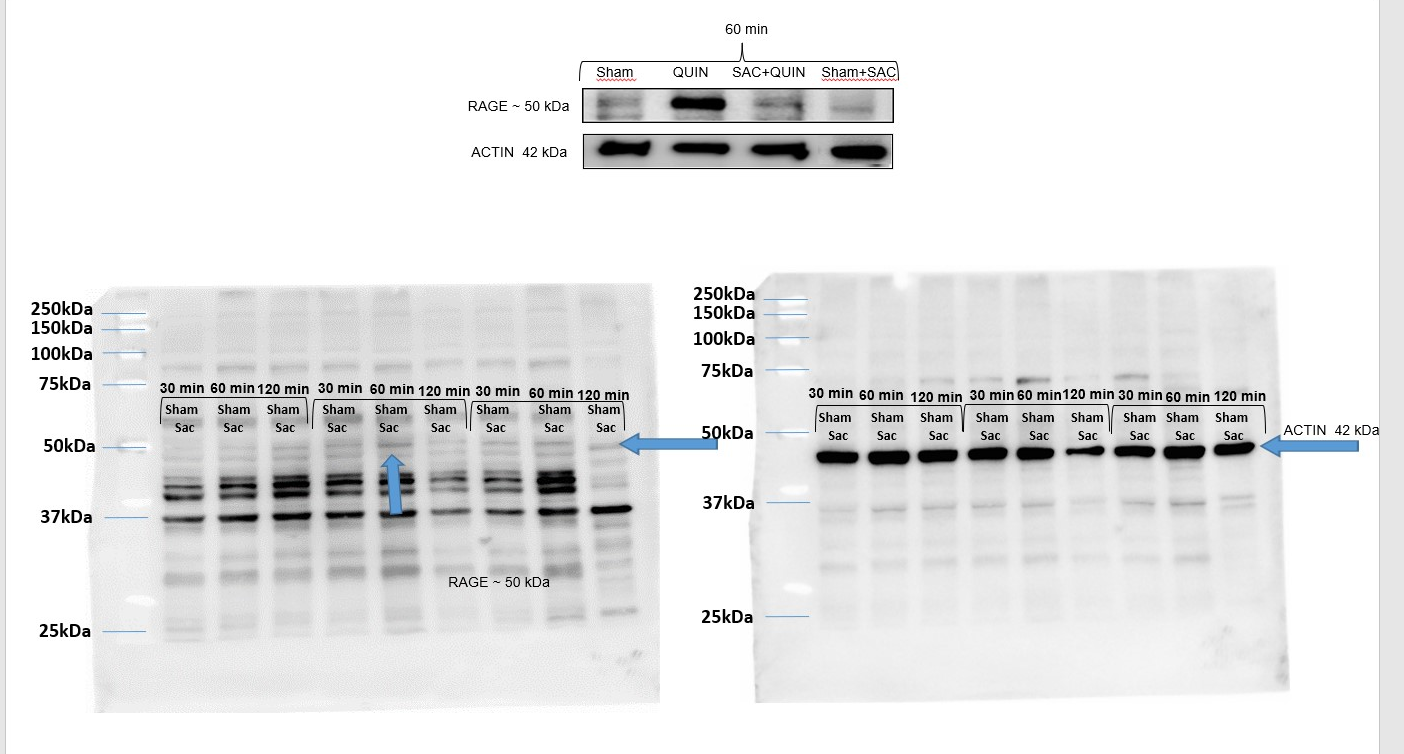
**

**
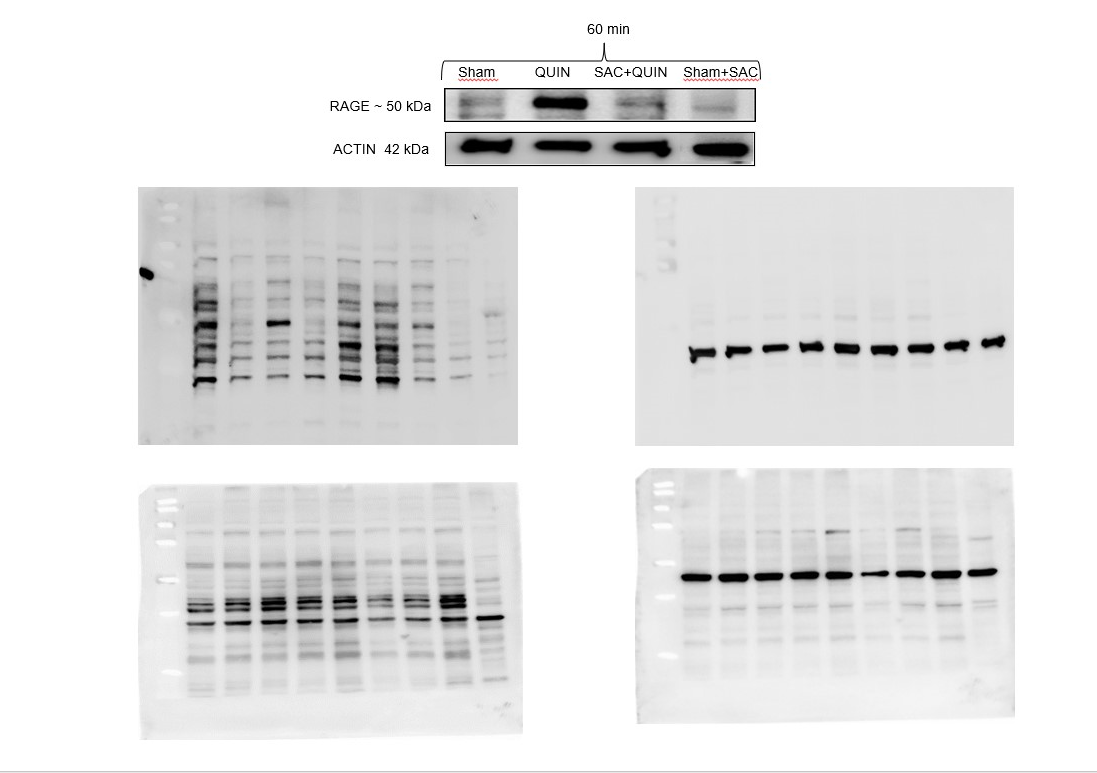
**

**
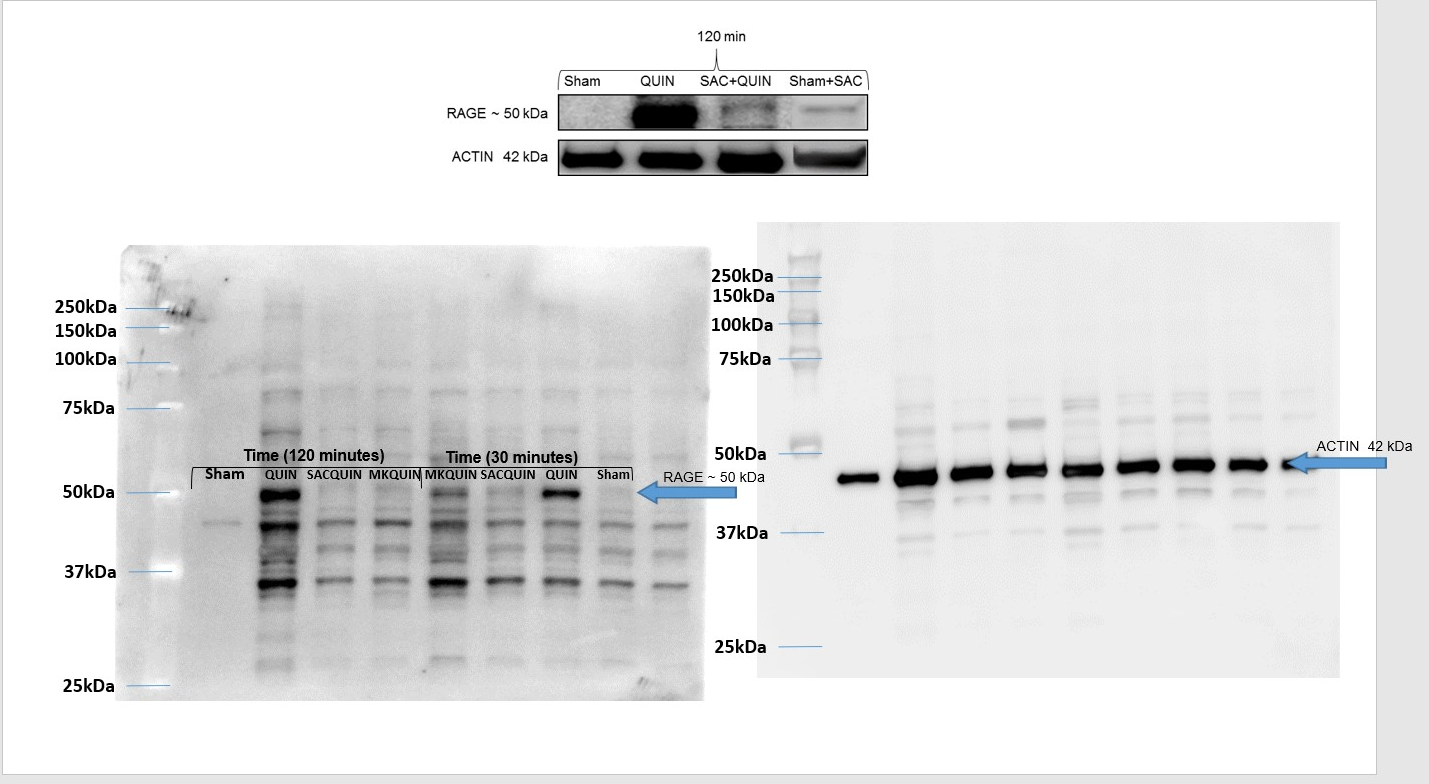
**

**
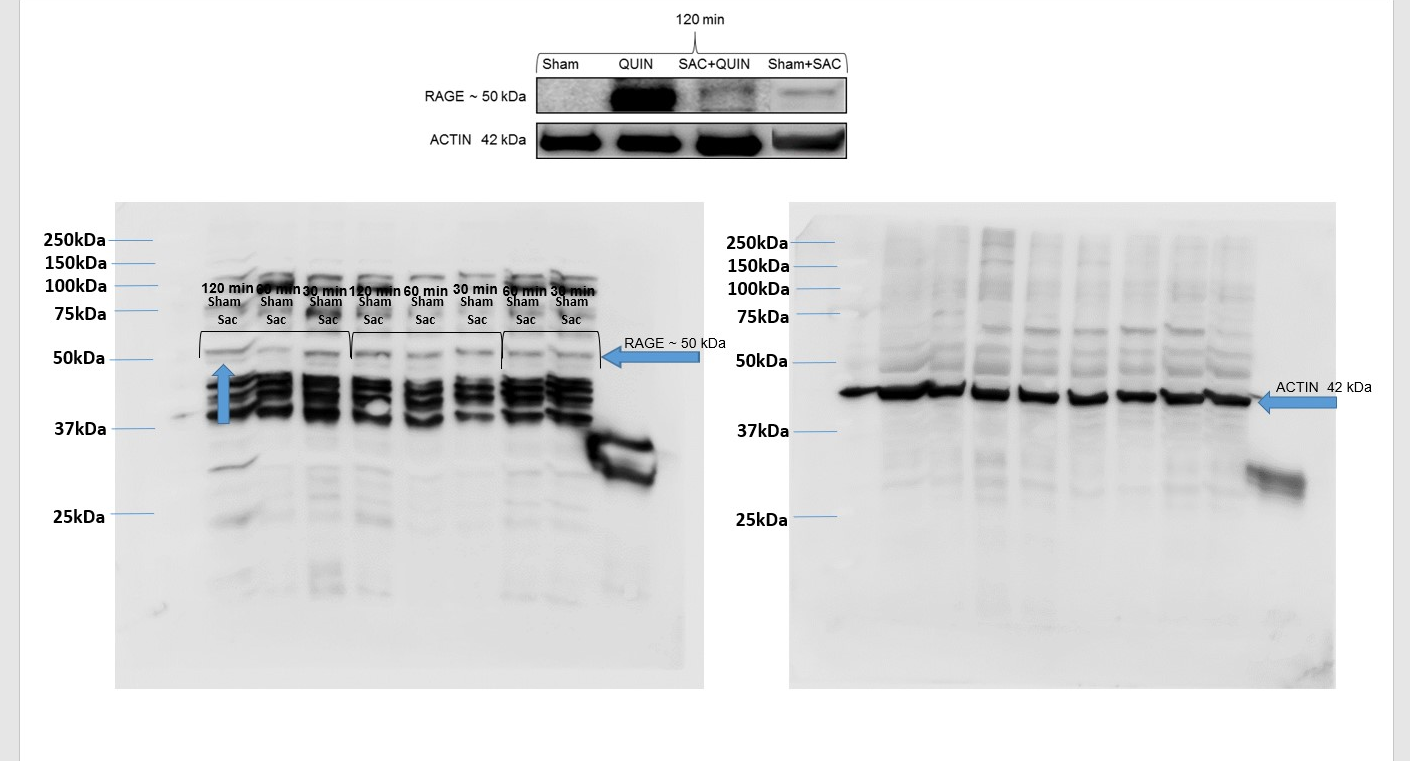
**

**
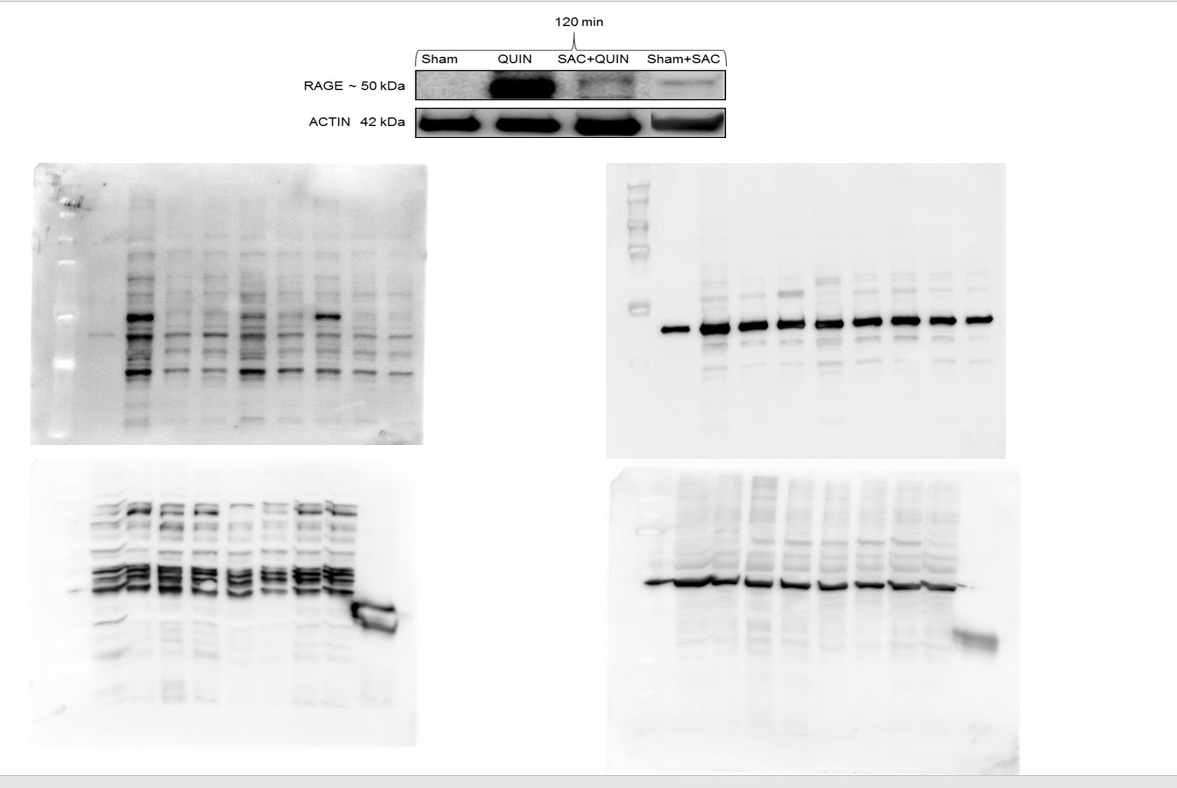
**

Supplement: S6 File — (DOCX) [file pone.0281905.s006.docx]
