# Supplementary material for: Expression of Concern: Modeling the Interaction between Quinolinate and the Receptor for Advanced Glycation End Products (RAGE): Relevance for Early Neuropathological Processes
Source: PLoS One. 2023 Feb 14;18(2):e0281905. doi: 10.1371/journal.pone.0281905 (PMC9928092; doi:10.1371/journal.pone.0281905)
Supplement: S3 File — (PPT) [file pone.0281905.s003.ppt]

## Slide 1
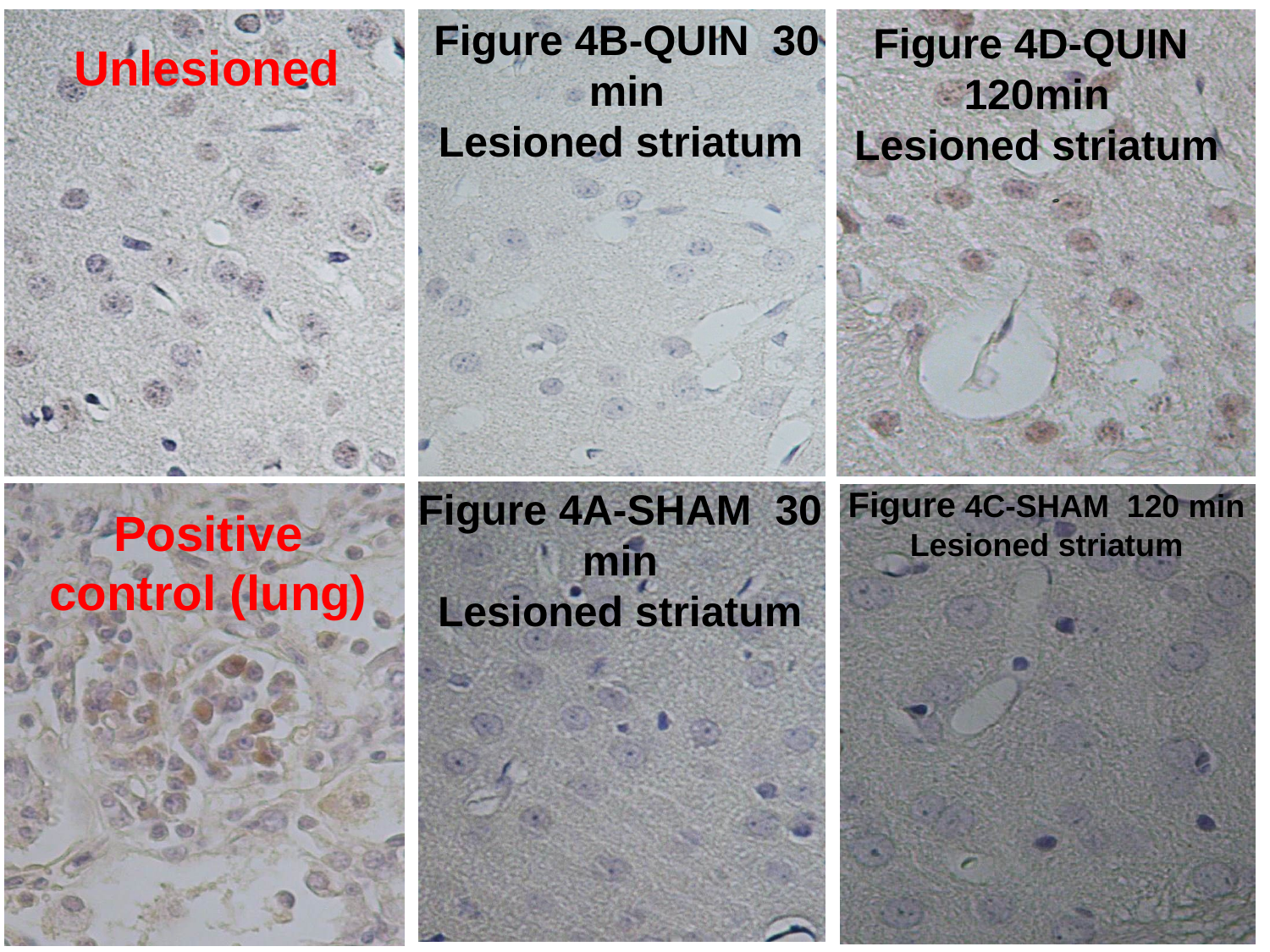

Figure 4B-QUIN 30 min
Lesioned striatum
Figure 4D-QUIN 120min
Lesioned striatum
Unlesioned
Figure 4A-SHAM 30 min
Lesioned striatum
Figure 4C-SHAM 120 min
Lesioned striatum
Positive control (lung)

## Slide 2
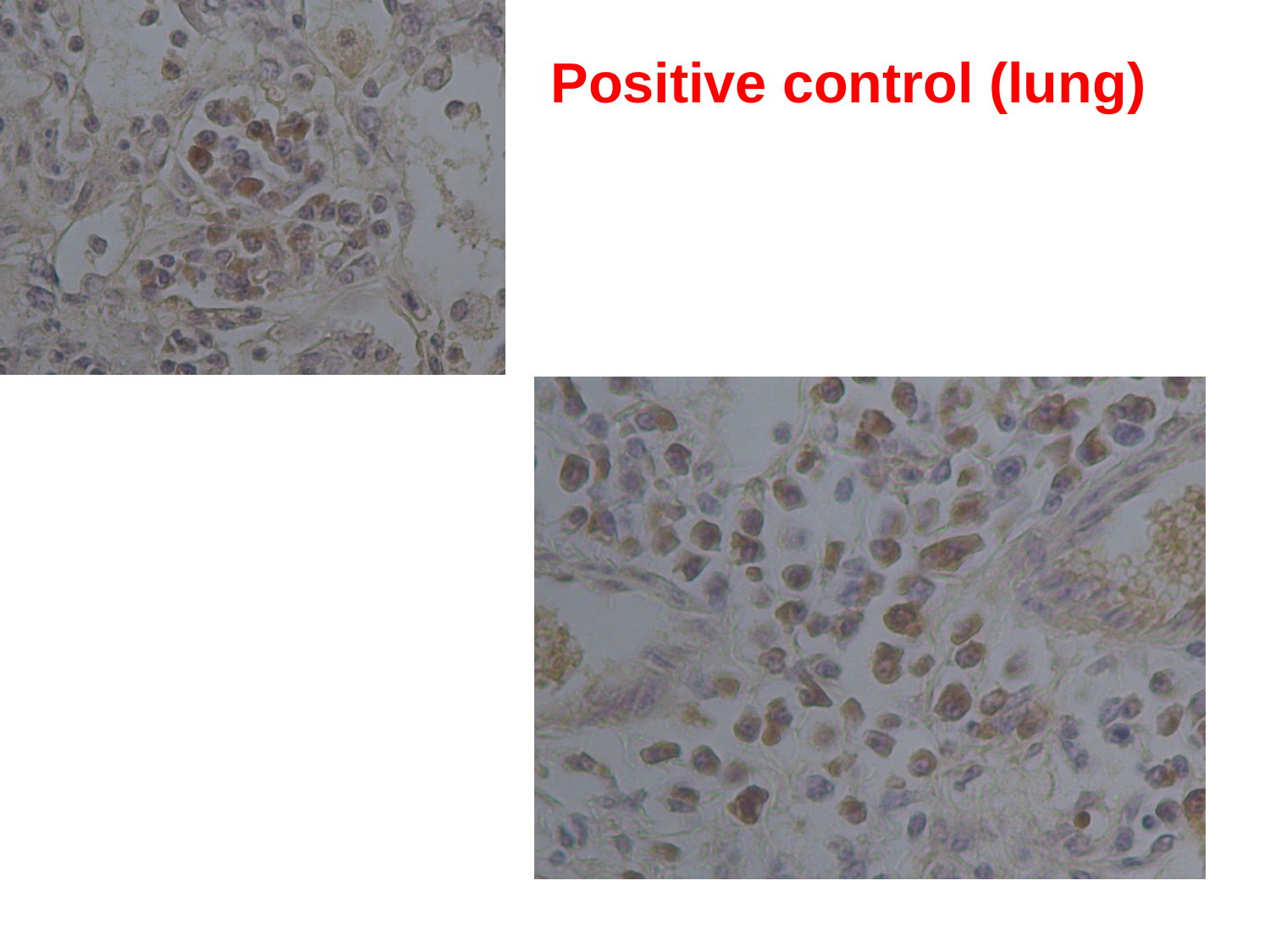

Positive control (lung)

## Slide 3
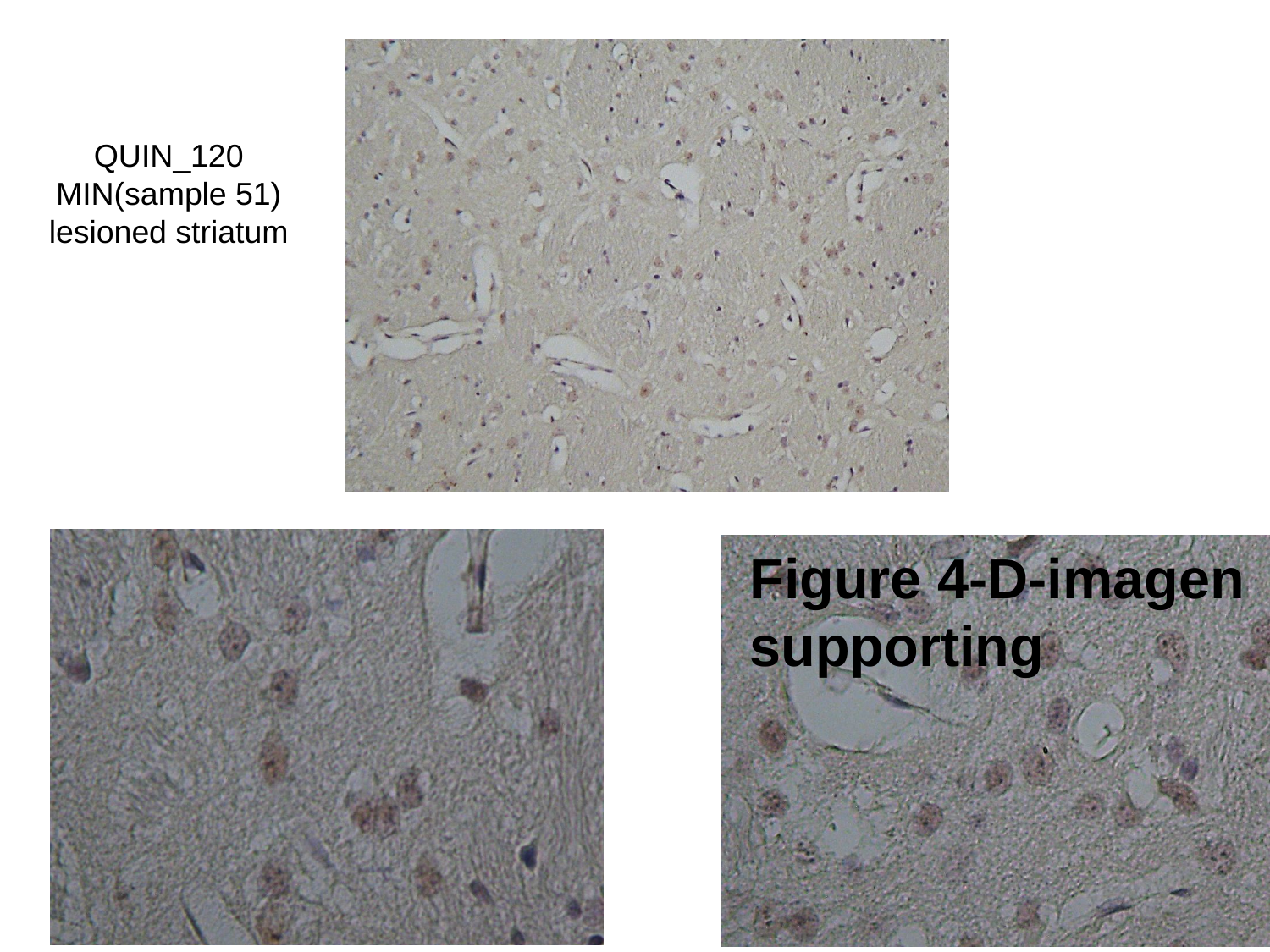

QUIN_120 MIN(sample 51)
lesioned striatum
Figure 4-D-imagen
supporting

## Slide 4
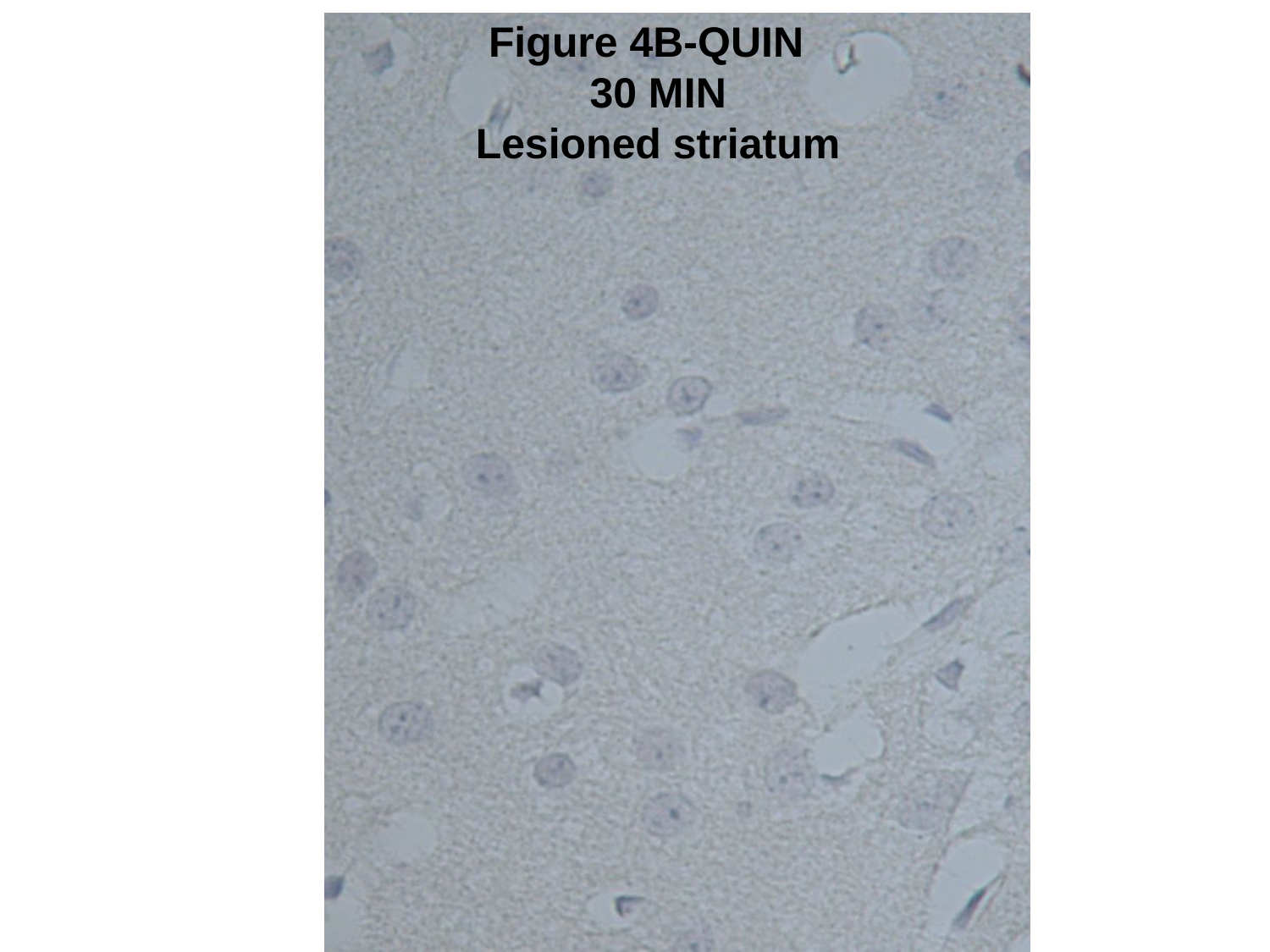

Figure 4B-QUIN
30 MIN
Lesioned striatum

## Slide 5
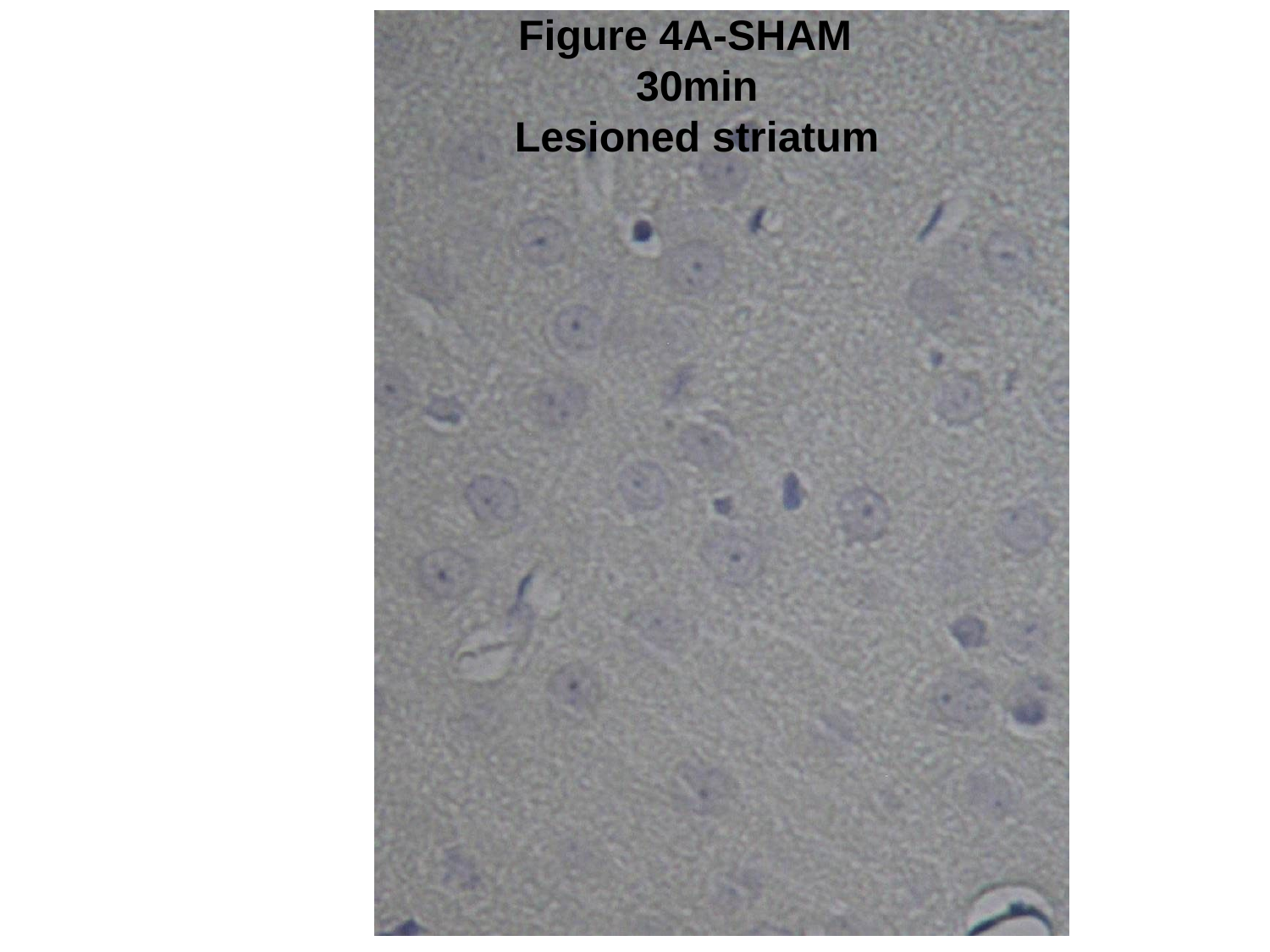

Figure 4A-SHAM
30min
Lesioned striatum

## Slide 6
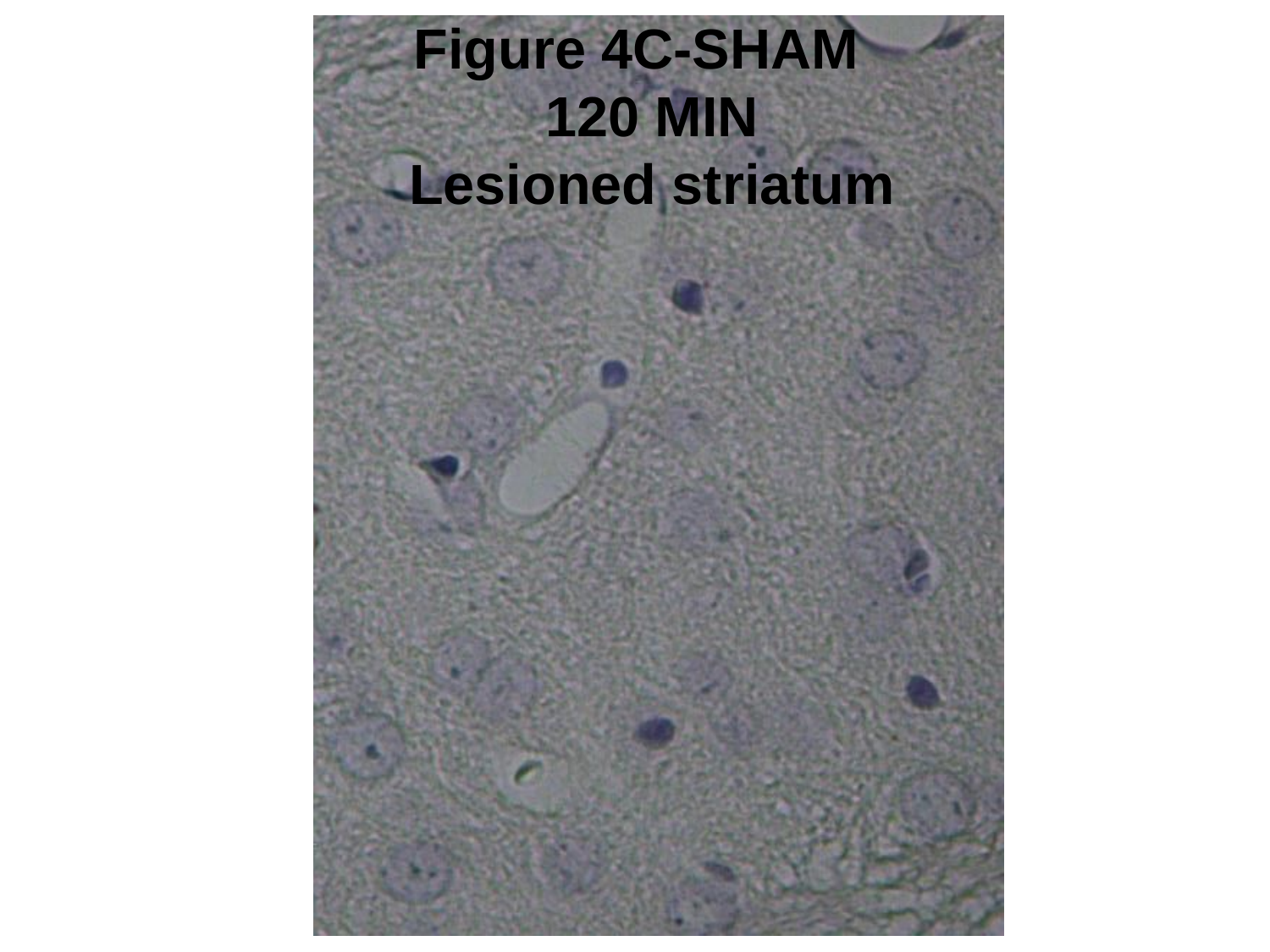

Figure 4C-SHAM
120 MIN
Lesioned striatum

## Slide 7
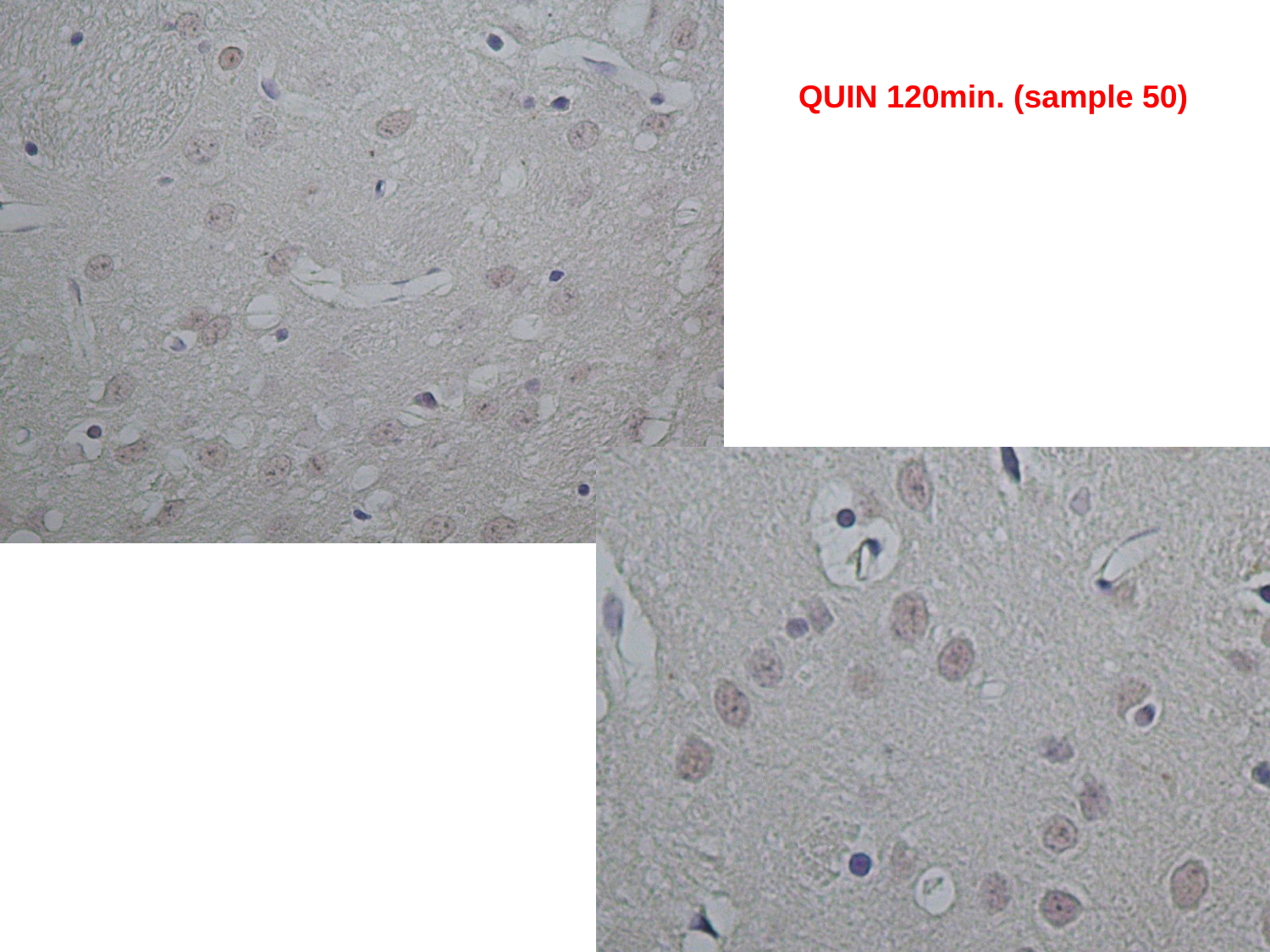

QUIN 120min. (sample 50)

## Slide 8
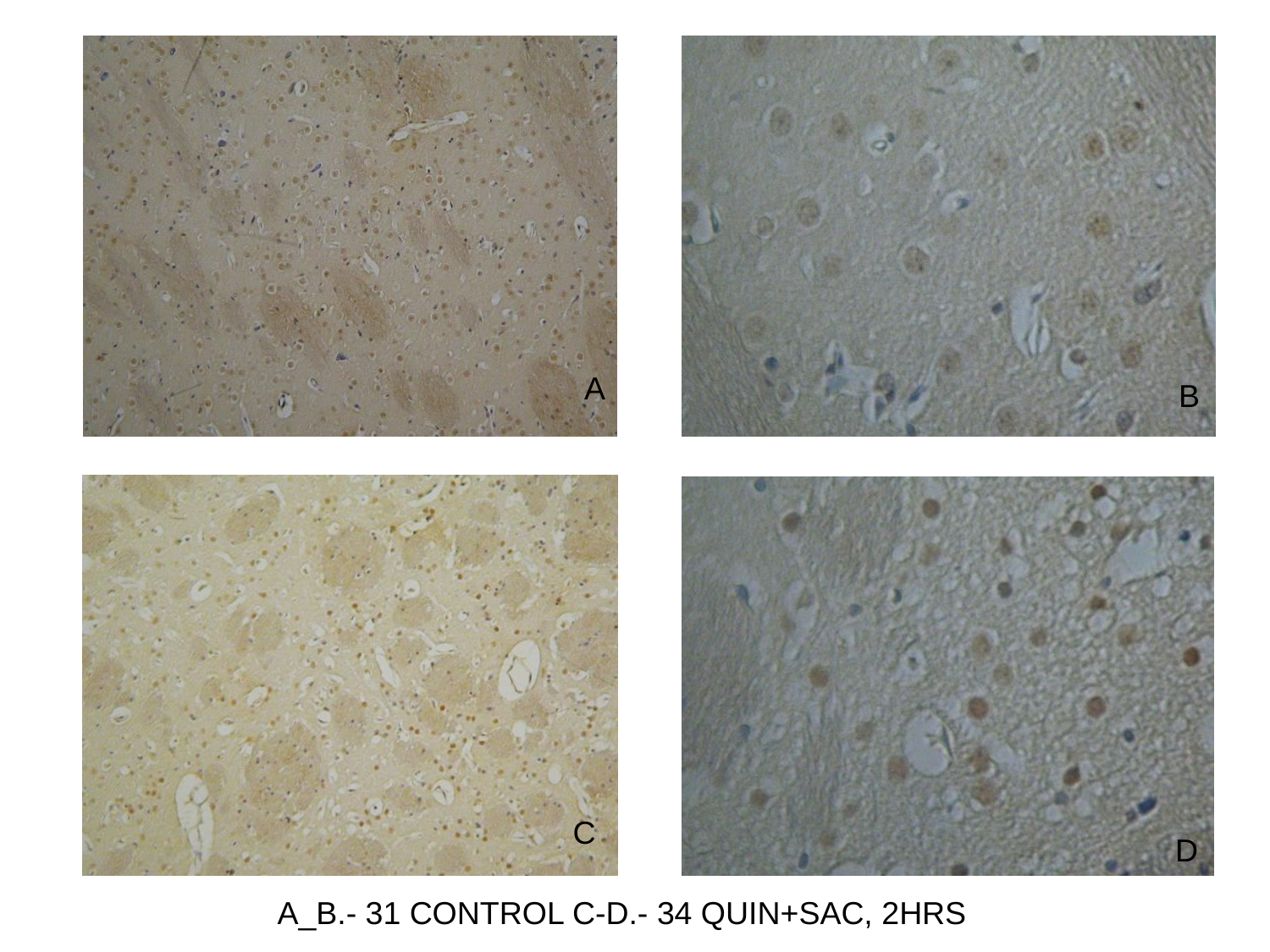

A
B
C
D
A_B.- 31 CONTROL C-D.- 34 QUIN+SAC, 2HRS
