# Supplementary material for: Expression of Concern: Modeling the Interaction between Quinolinate and the Receptor for Advanced Glycation End Products (RAGE): Relevance for Early Neuropathological Processes
Source: PLoS One. 2023 Feb 14;18(2):e0281905. doi: 10.1371/journal.pone.0281905 (PMC9928092; doi:10.1371/journal.pone.0281905)
Supplement: S4 File — (PPT) [file pone.0281905.s004.ppt]

## Slide 1
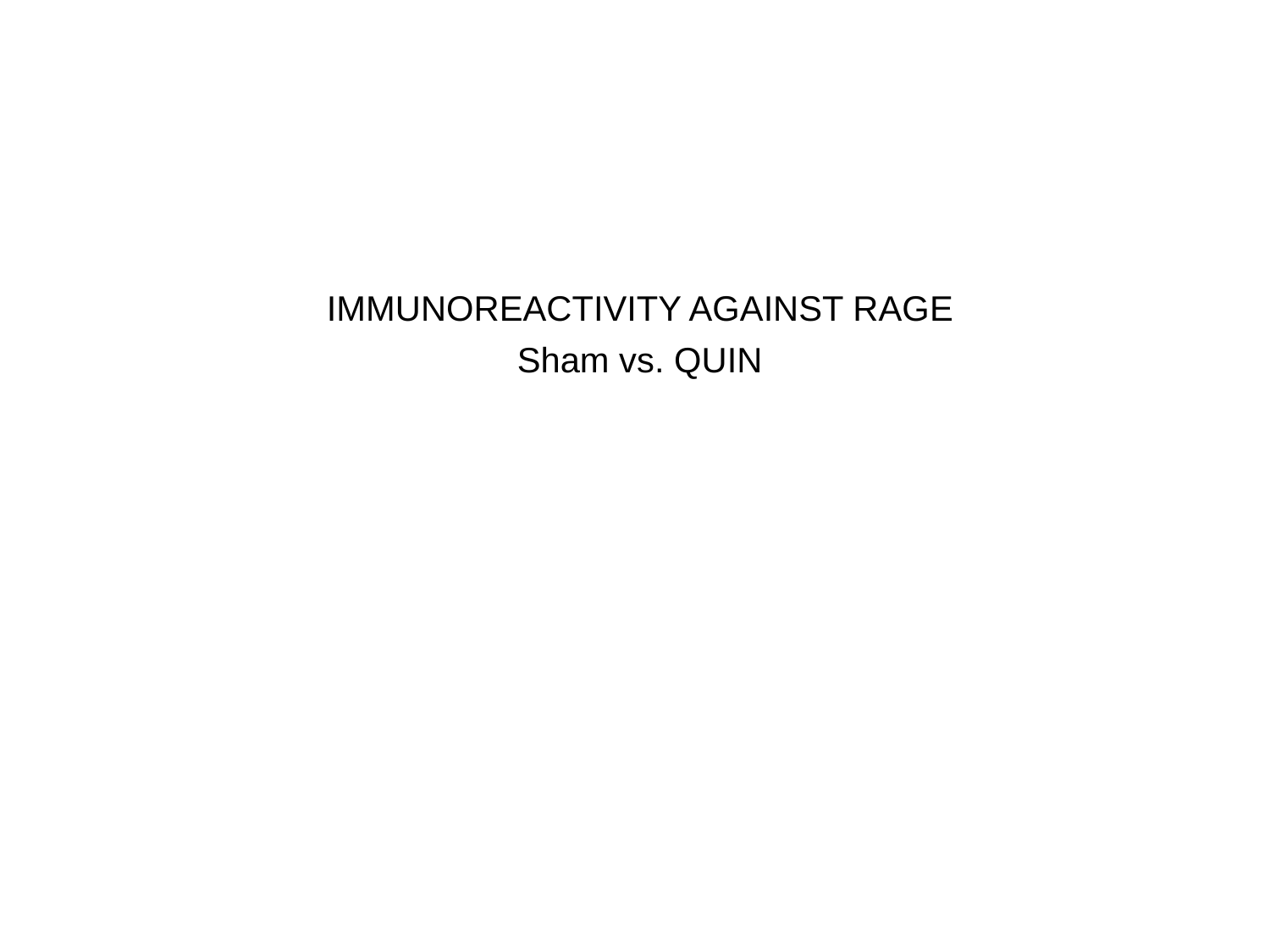

# IMMUNOREACTIVITY AGAINST RAGE
Sham vs. QUIN

## Slide 2
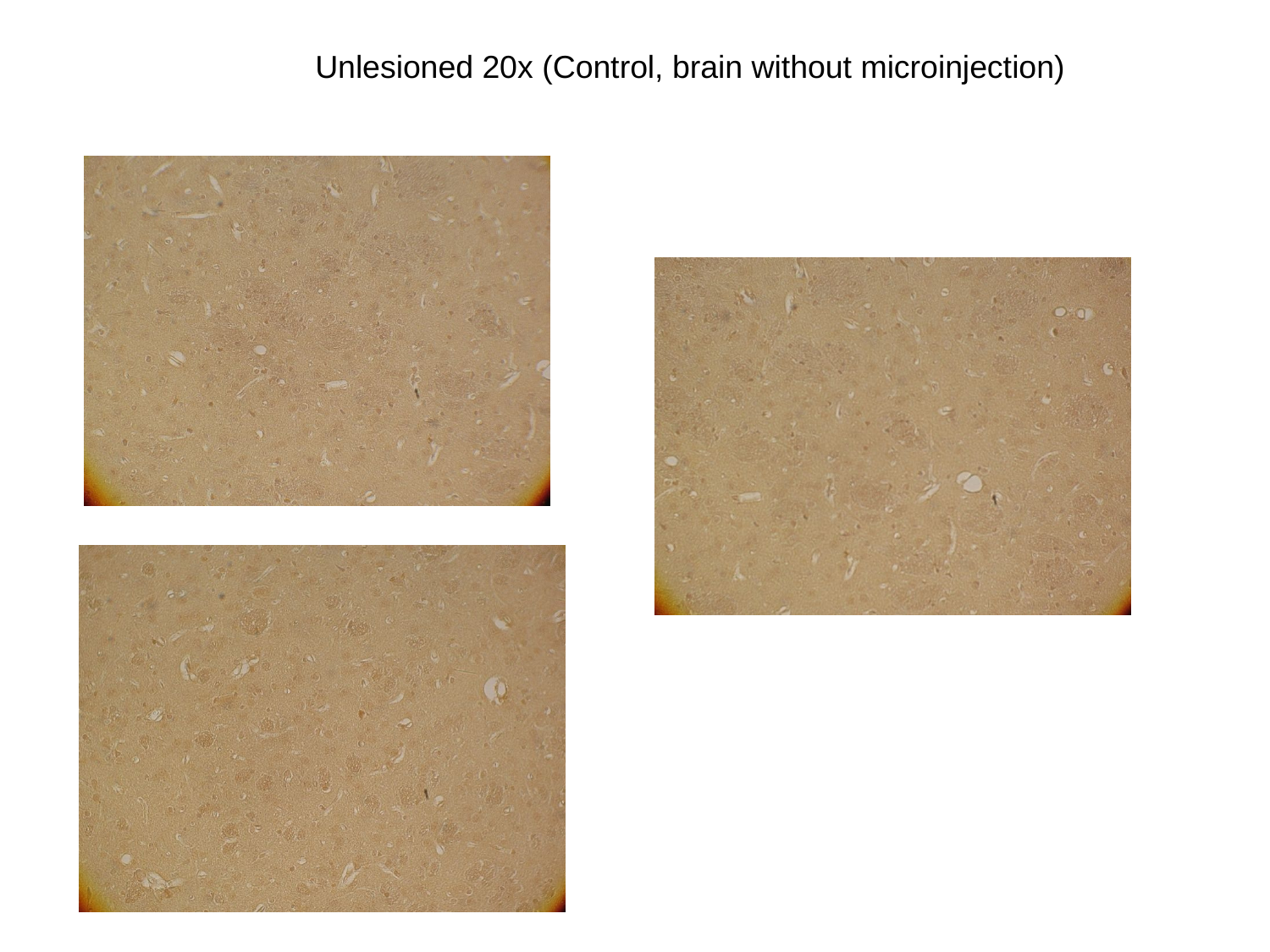

Unlesioned 20x (Control, brain without microinjection)

## Slide 3
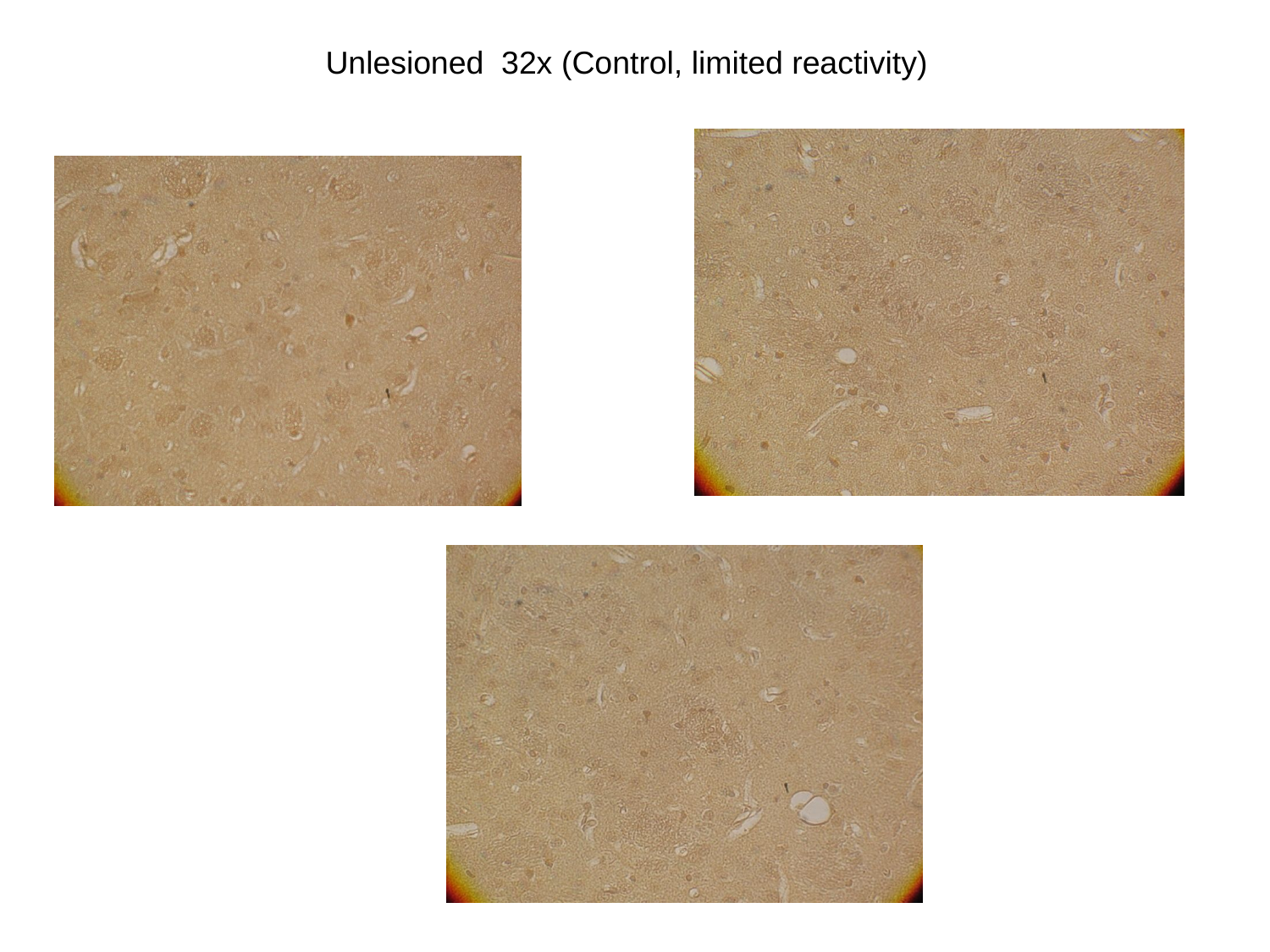

Unlesioned 32x (Control, limited reactivity)

## Slide 4
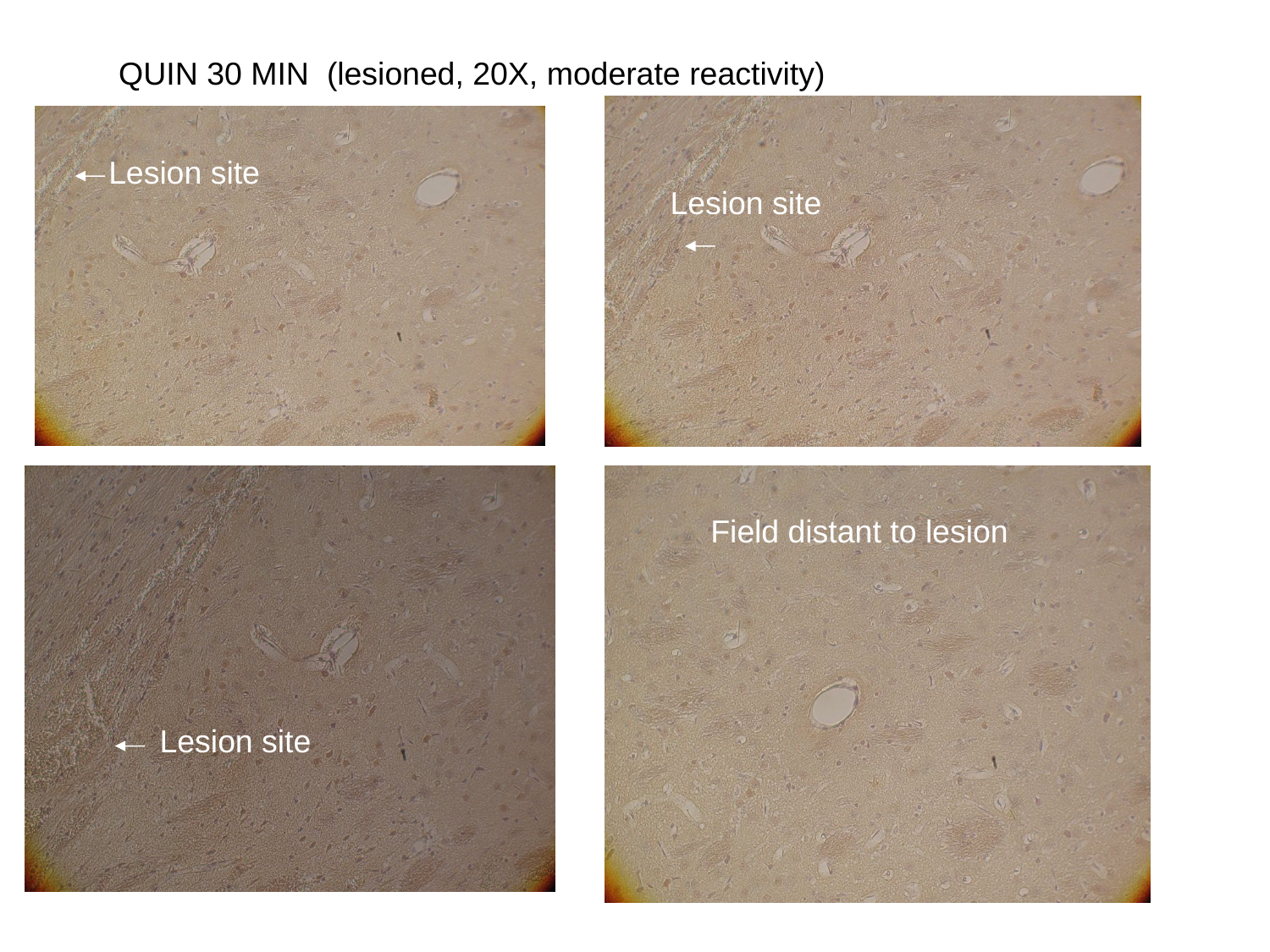

QUIN 30 MIN (lesioned, 20X, moderate reactivity)
Lesion site
Lesion site
Field distant to lesion
Lesion site

## Slide 5
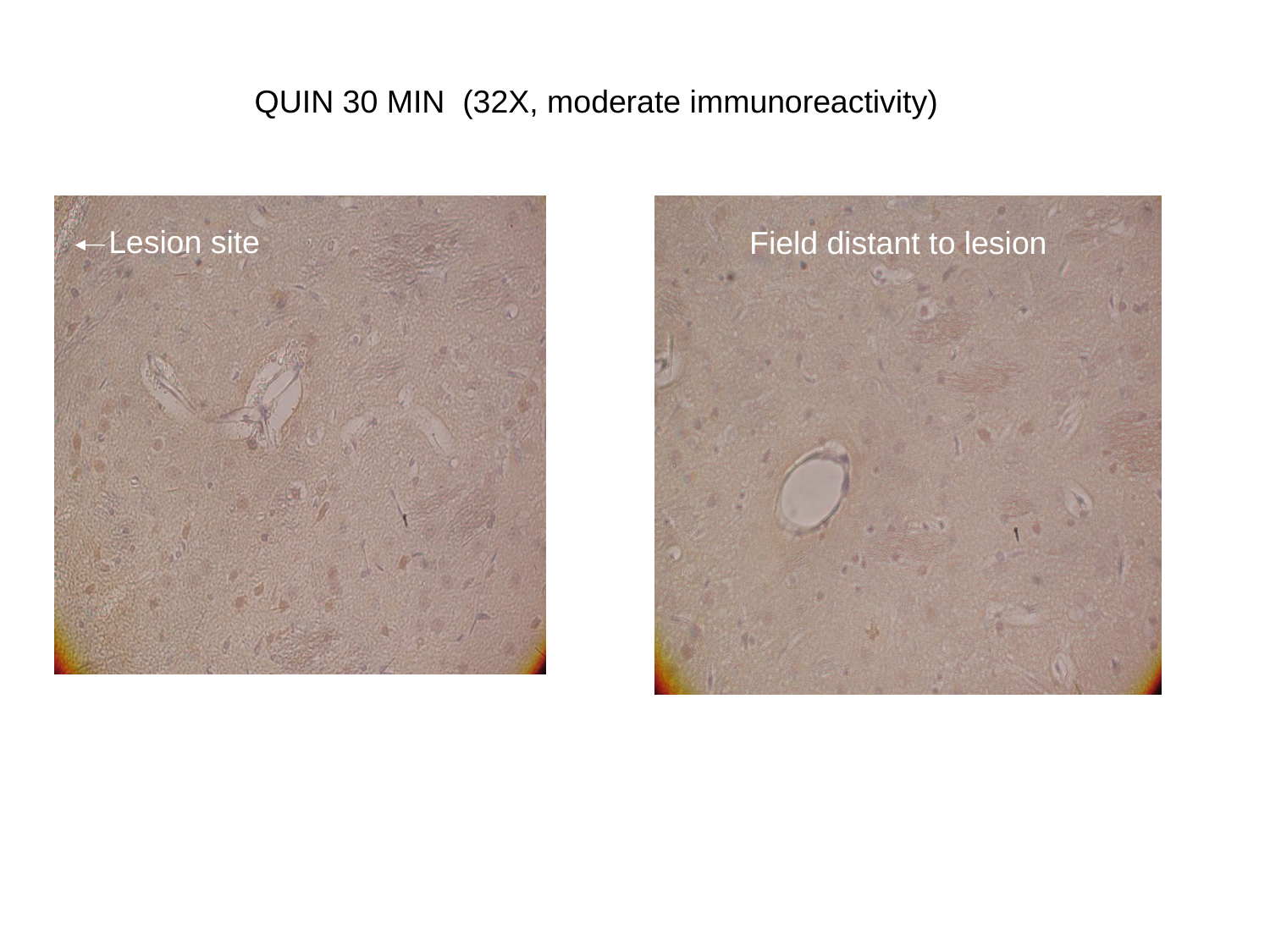

QUIN 30 MIN (32X, moderate immunoreactivity)
Lesion site
Field distant to lesion

## Slide 6
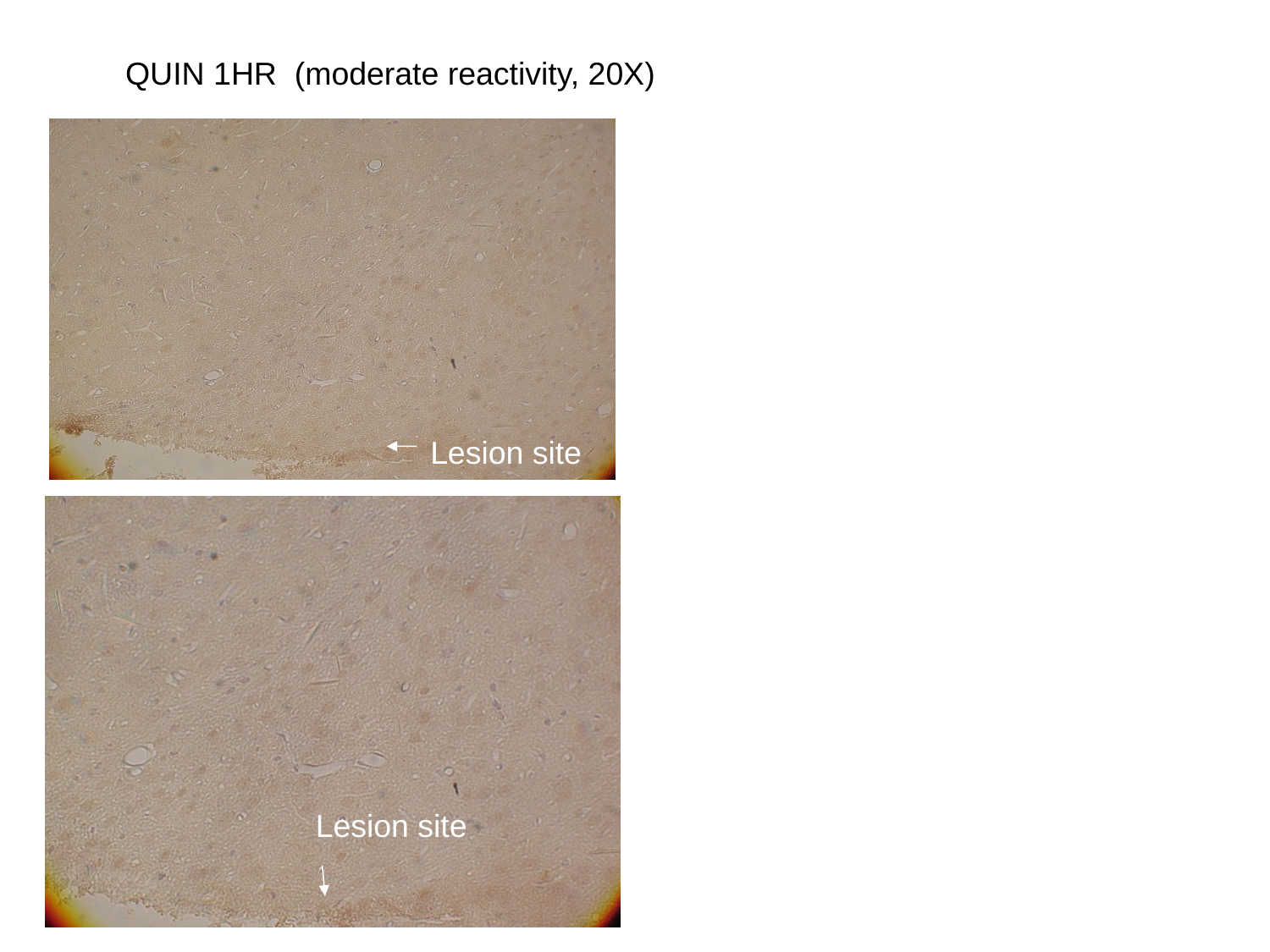

QUIN 1HR (moderate reactivity, 20X)
Lesion site
Lesion site

## Slide 7
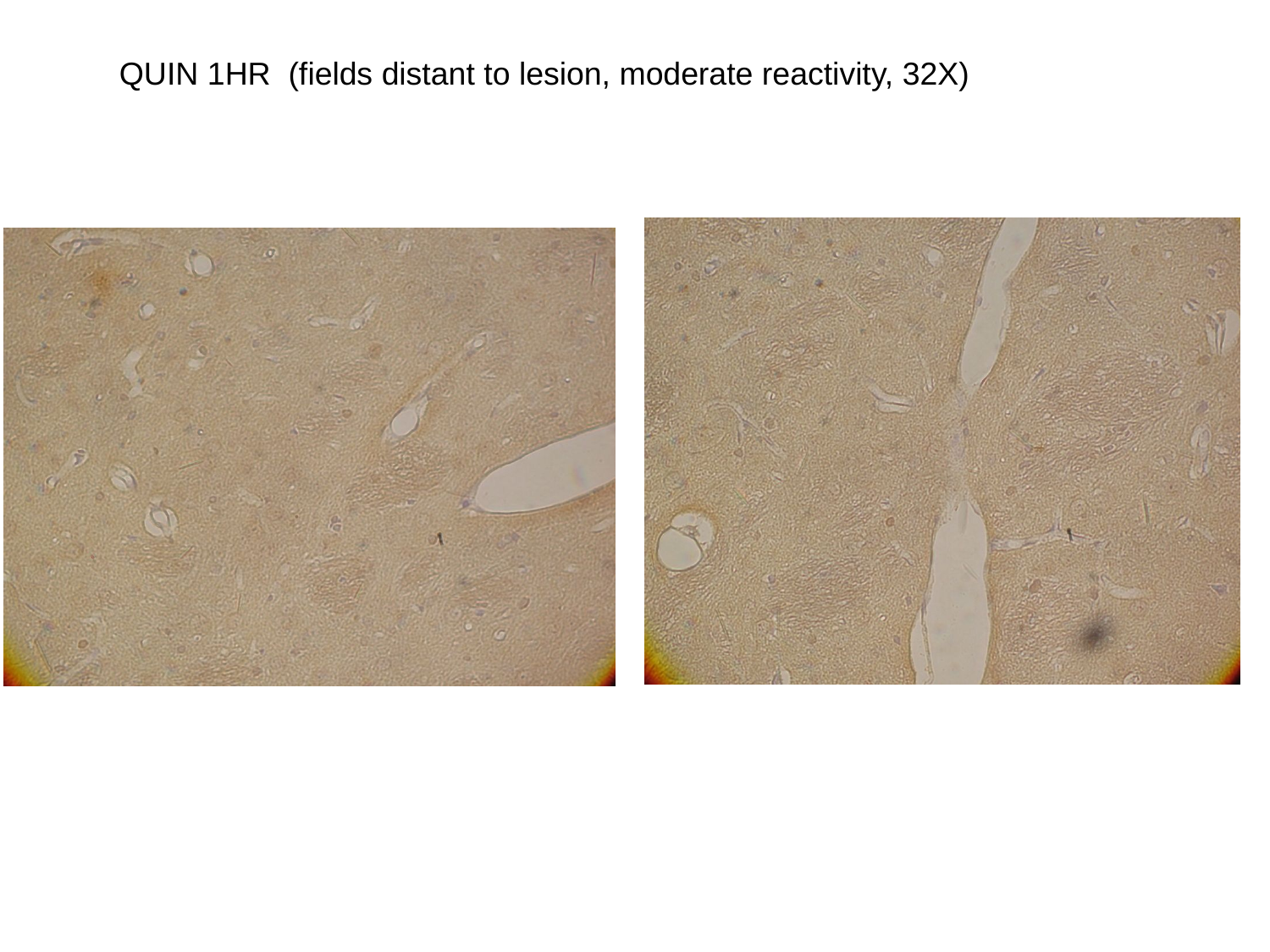

QUIN 1HR (fields distant to lesion, moderate reactivity, 32X)

## Slide 8
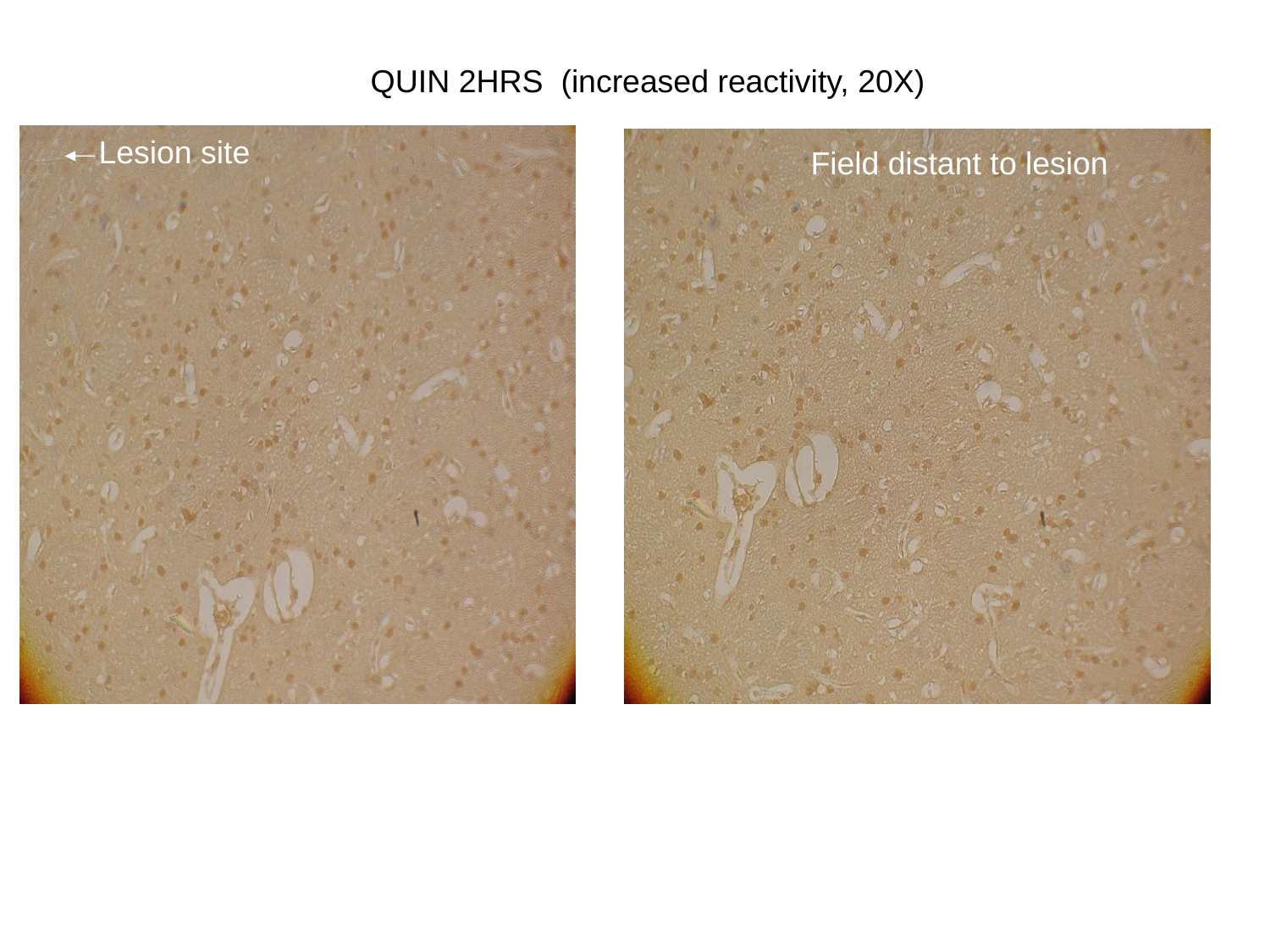

QUIN 2HRS (increased reactivity, 20X)
Lesion site
Field distant to lesion

## Slide 9
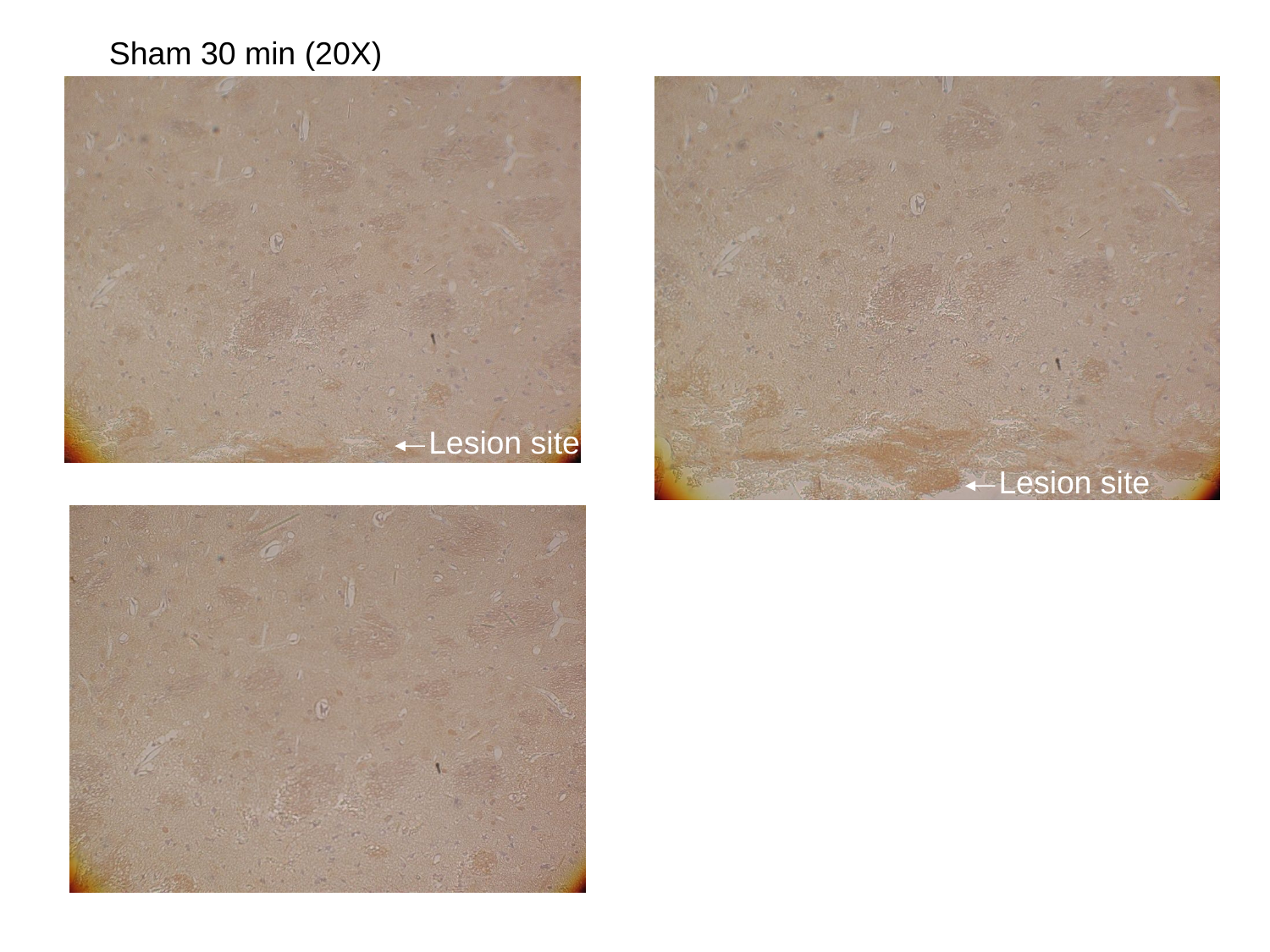

Sham 30 min (20X)
Lesion site
Lesion site

## Slide 10
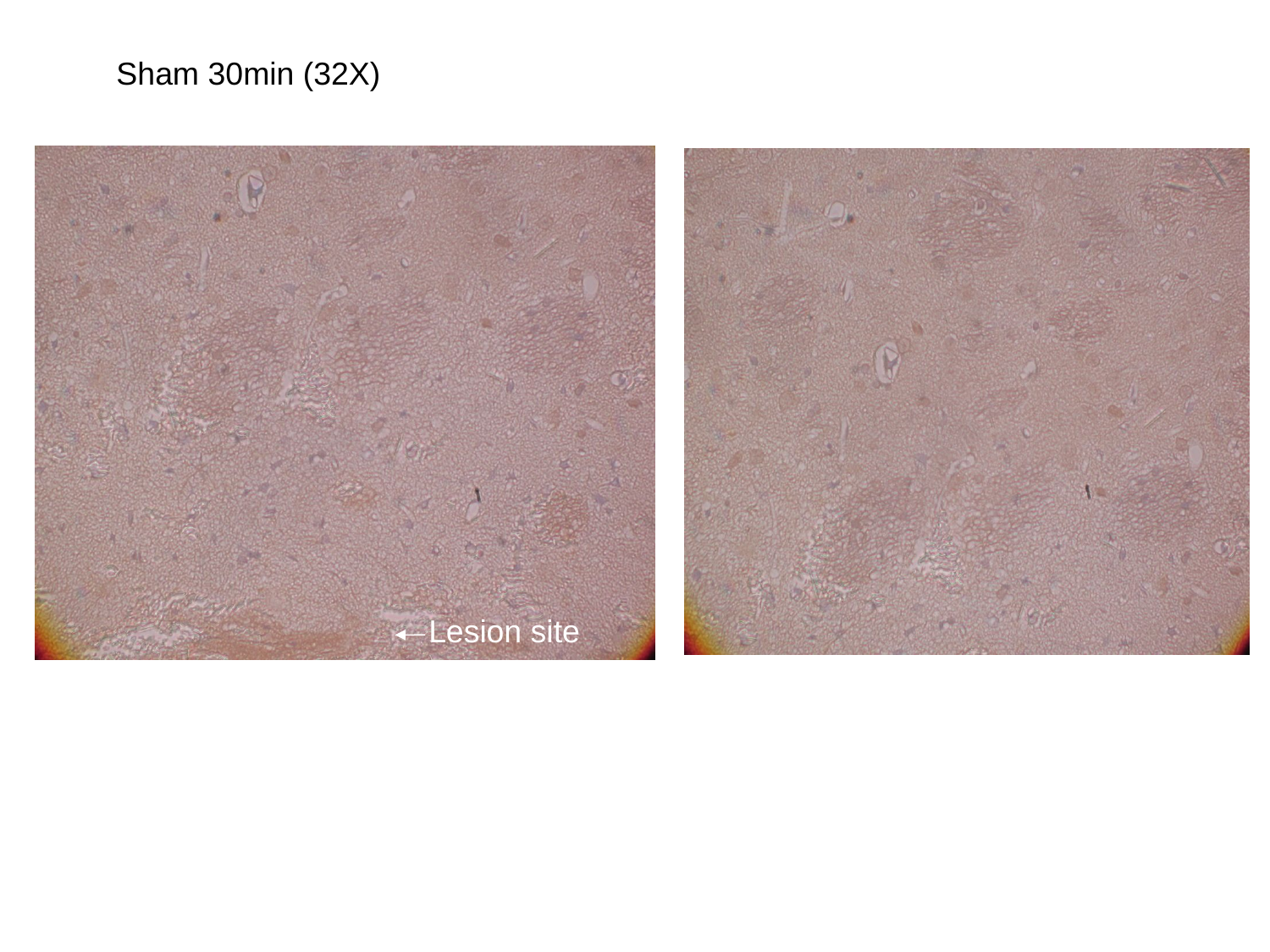

Sham 30min (32X)
Lesion site

## Slide 11
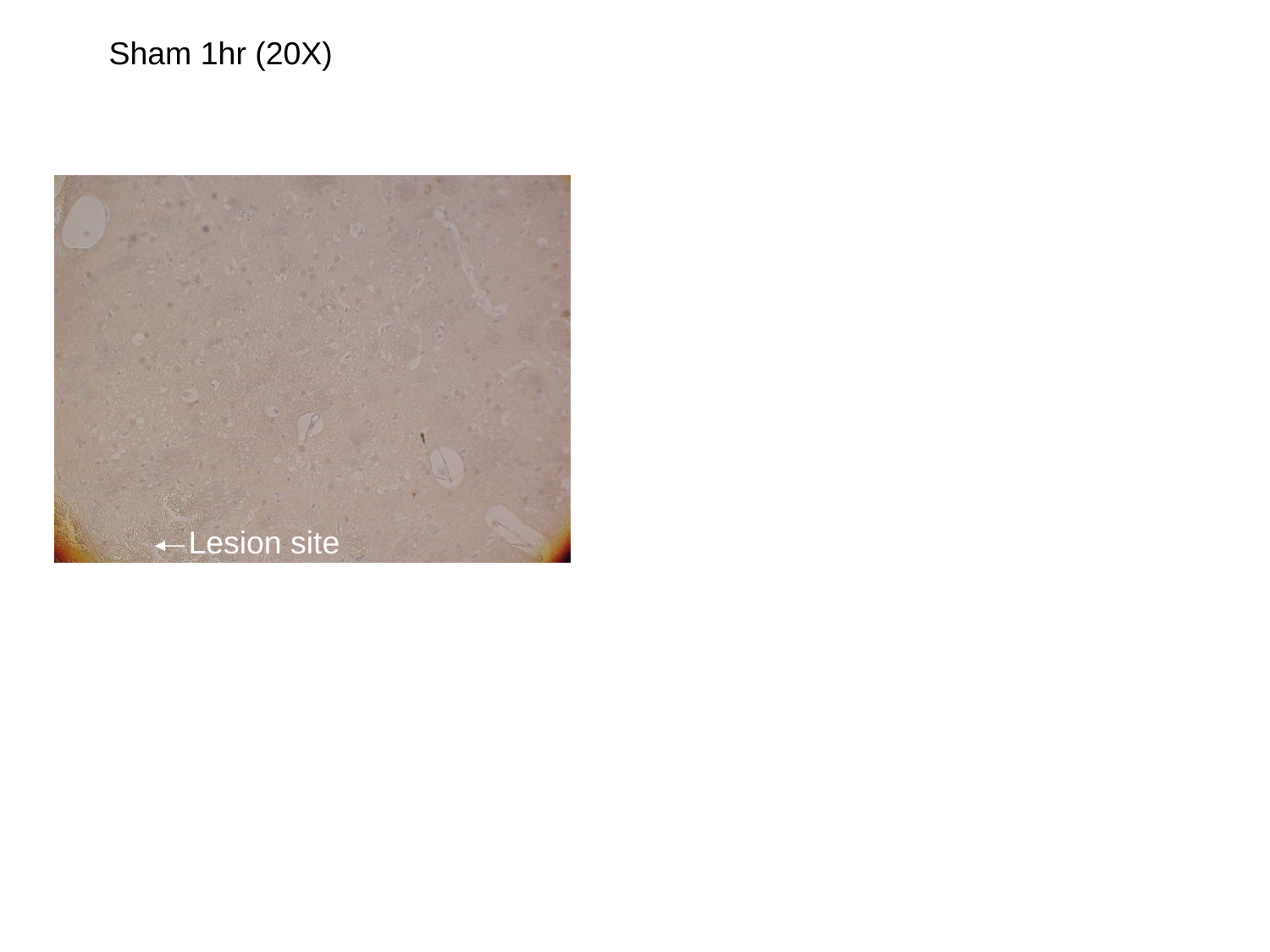

Sham 1hr (20X)
Lesion site

## Slide 12
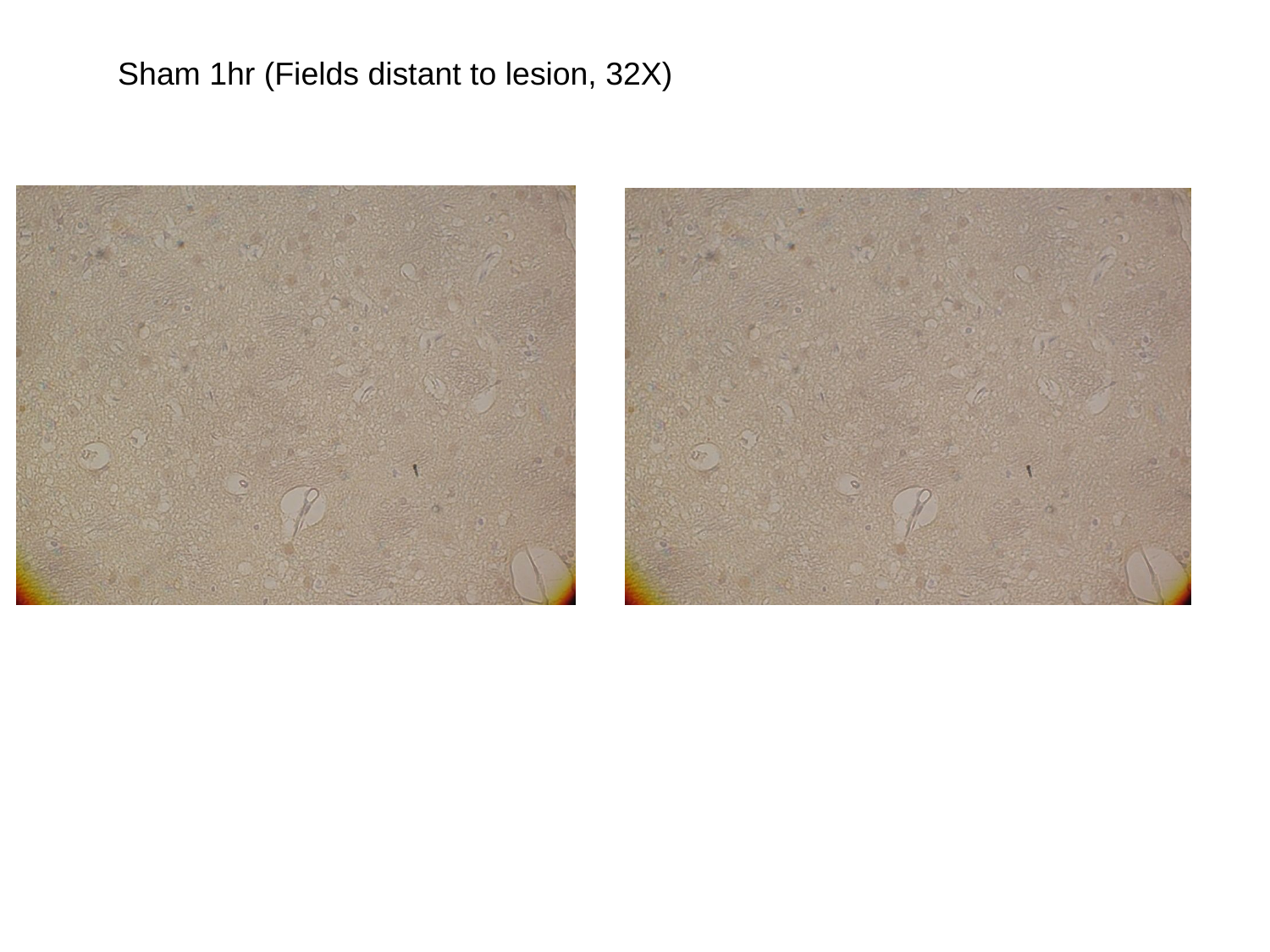

Sham 1hr (Fields distant to lesion, 32X)

## Slide 13
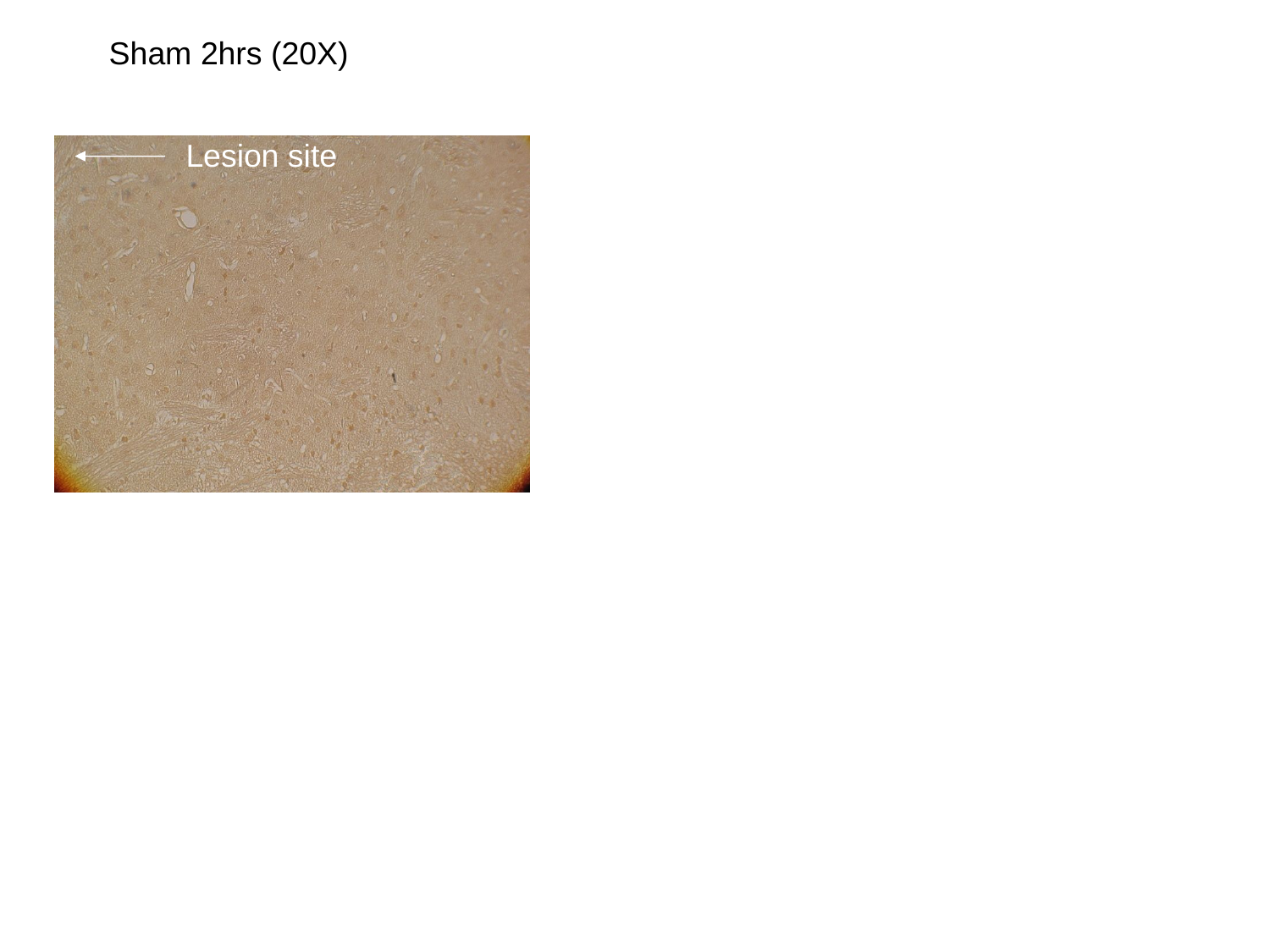

Sham 2hrs (20X)
Lesion site

## Slide 14
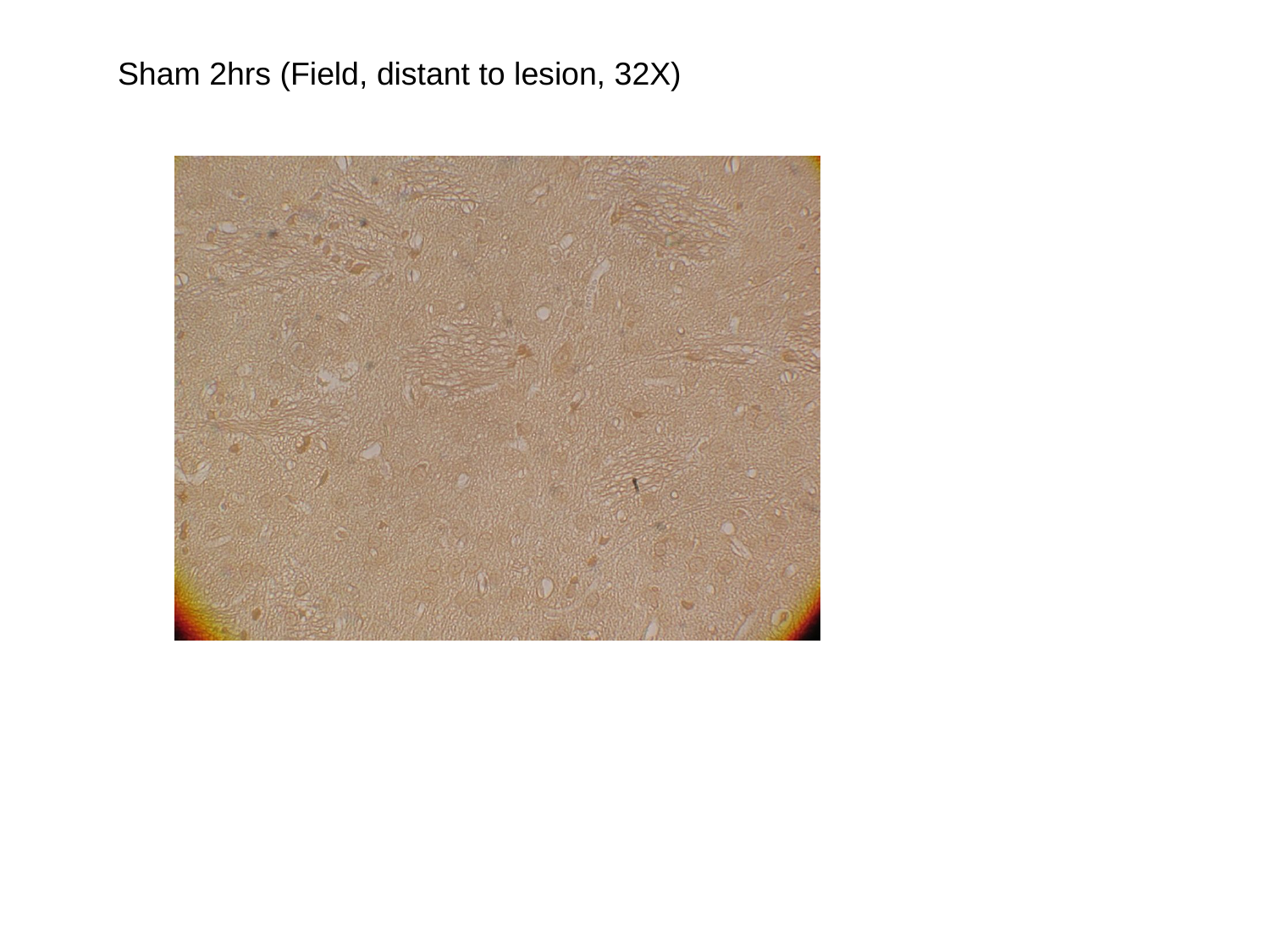

Sham 2hrs (Field, distant to lesion, 32X)

## Slide 15
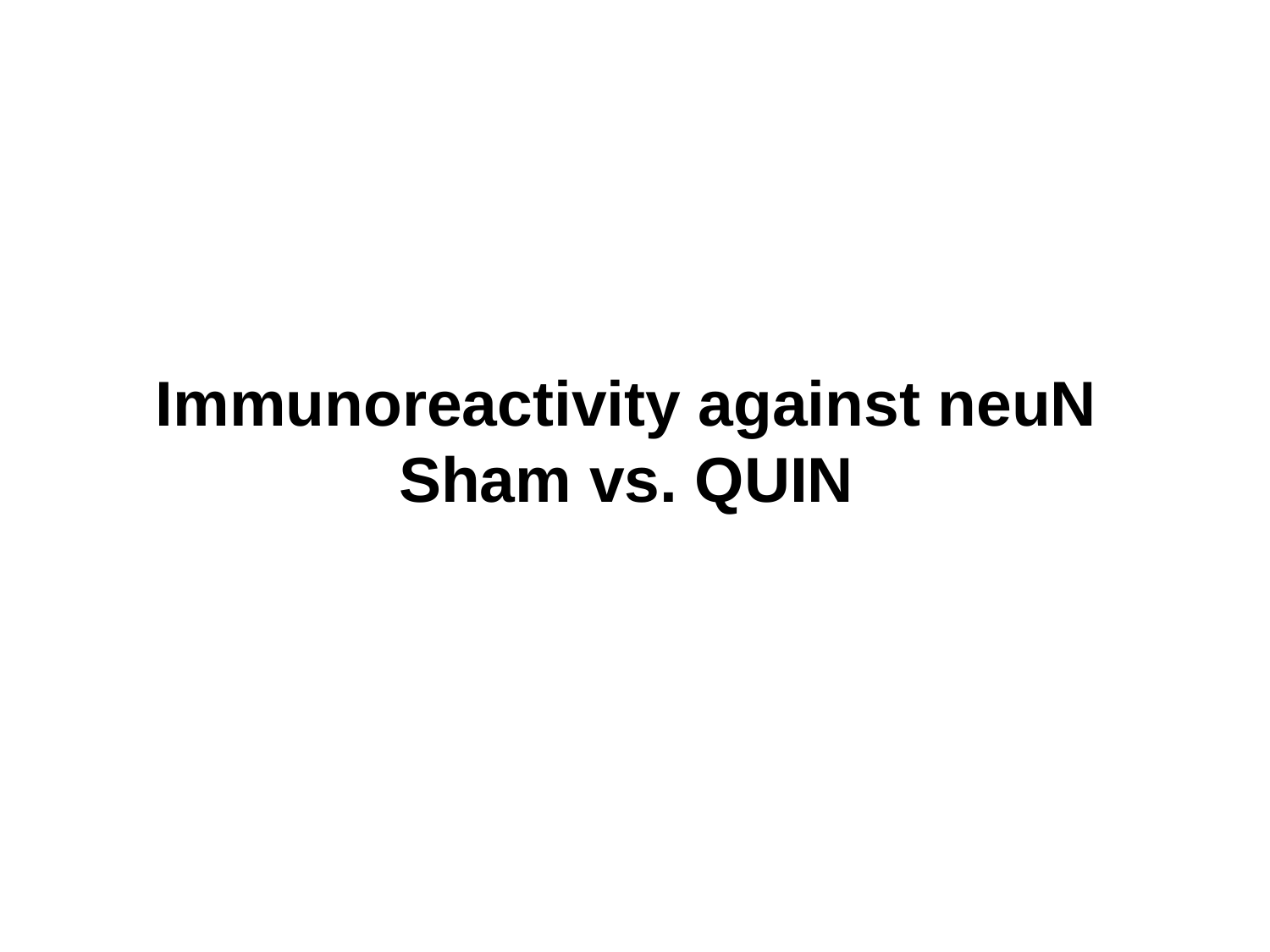

Immunoreactivity against neuN
Sham vs. QUIN

## Slide 16
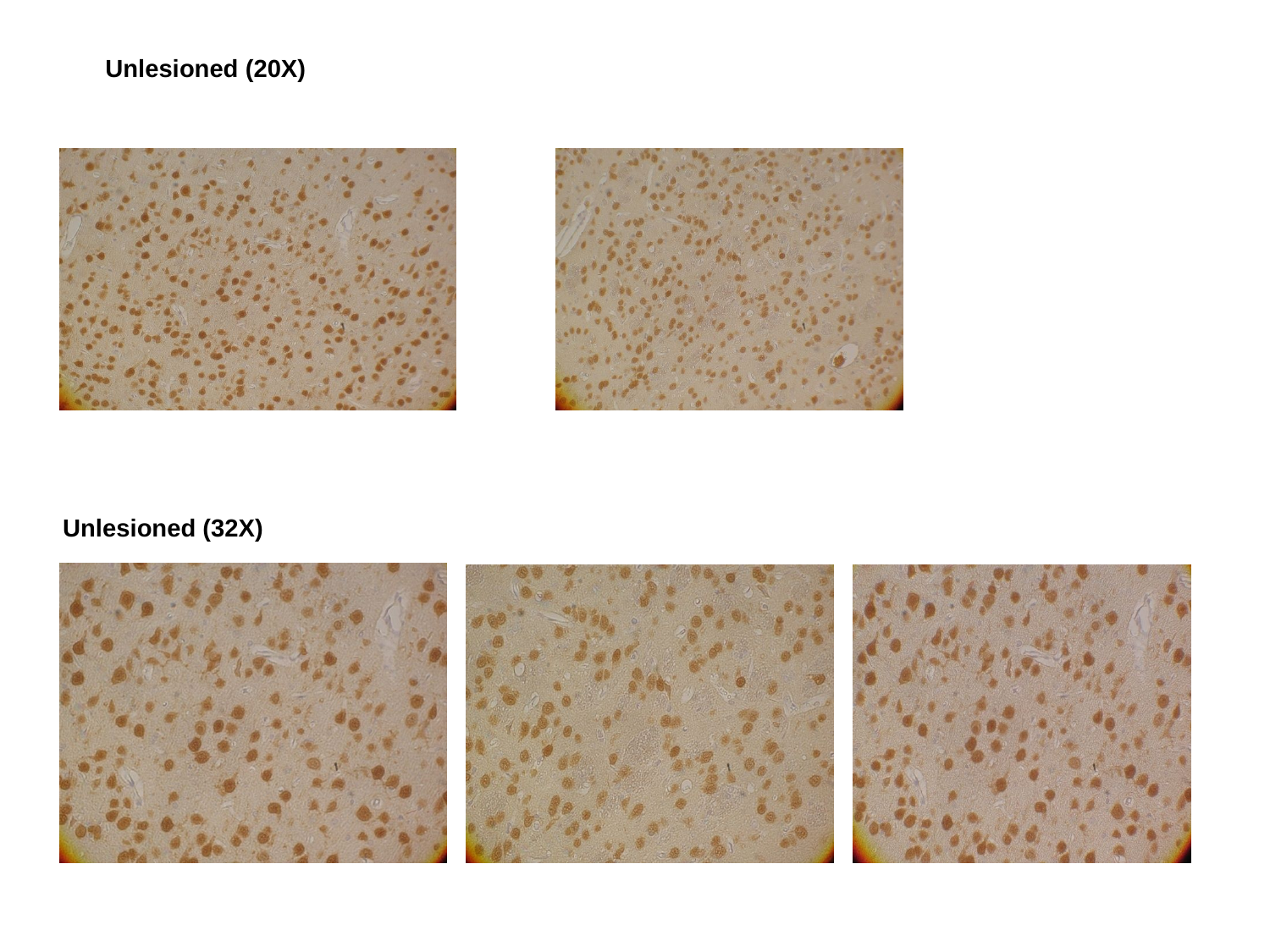

Unlesioned (20X)
Unlesioned (32X)

## Slide 17
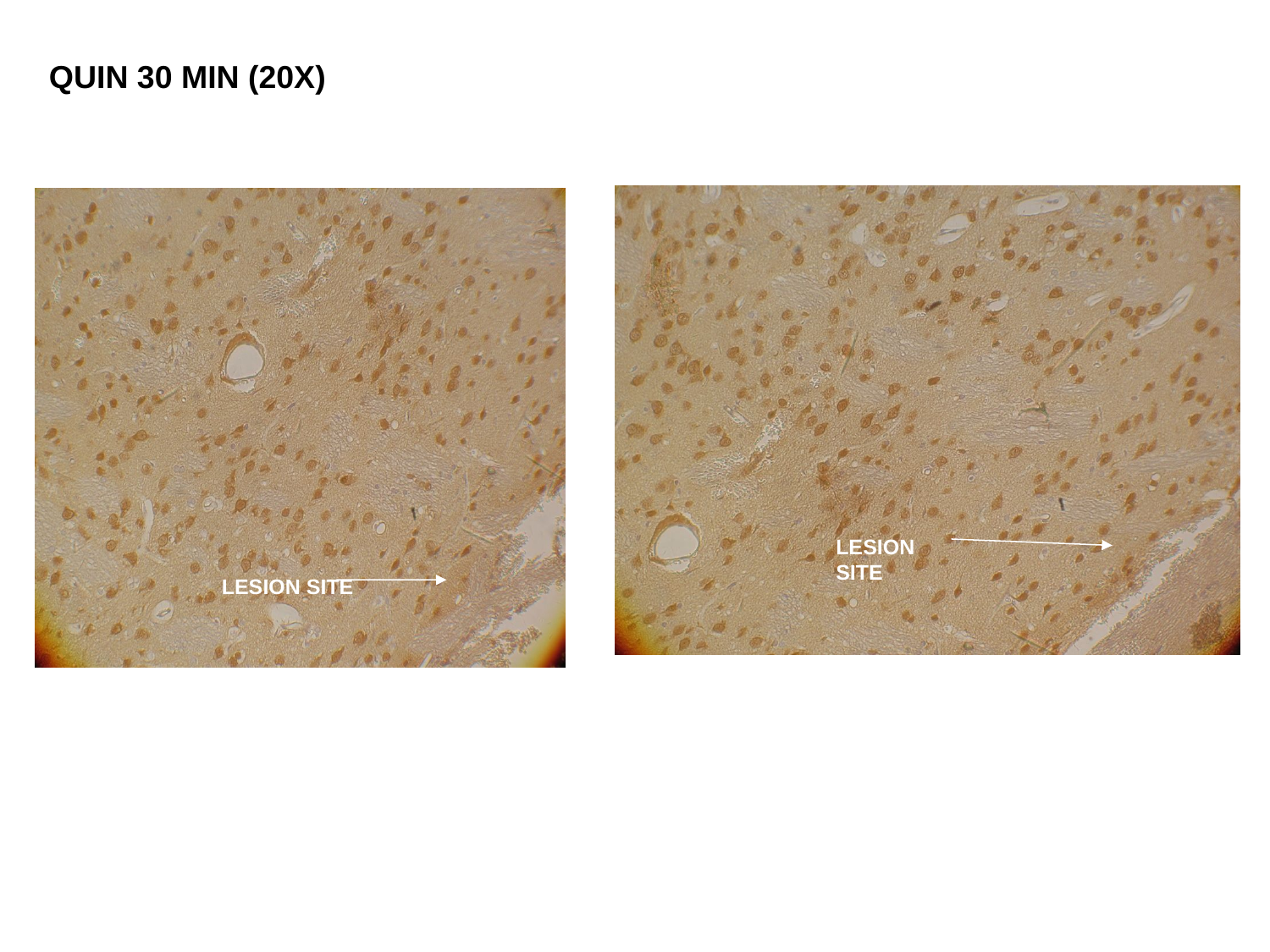

QUIN 30 MIN (20X)
LESION SITE
LESION SITE

## Slide 18
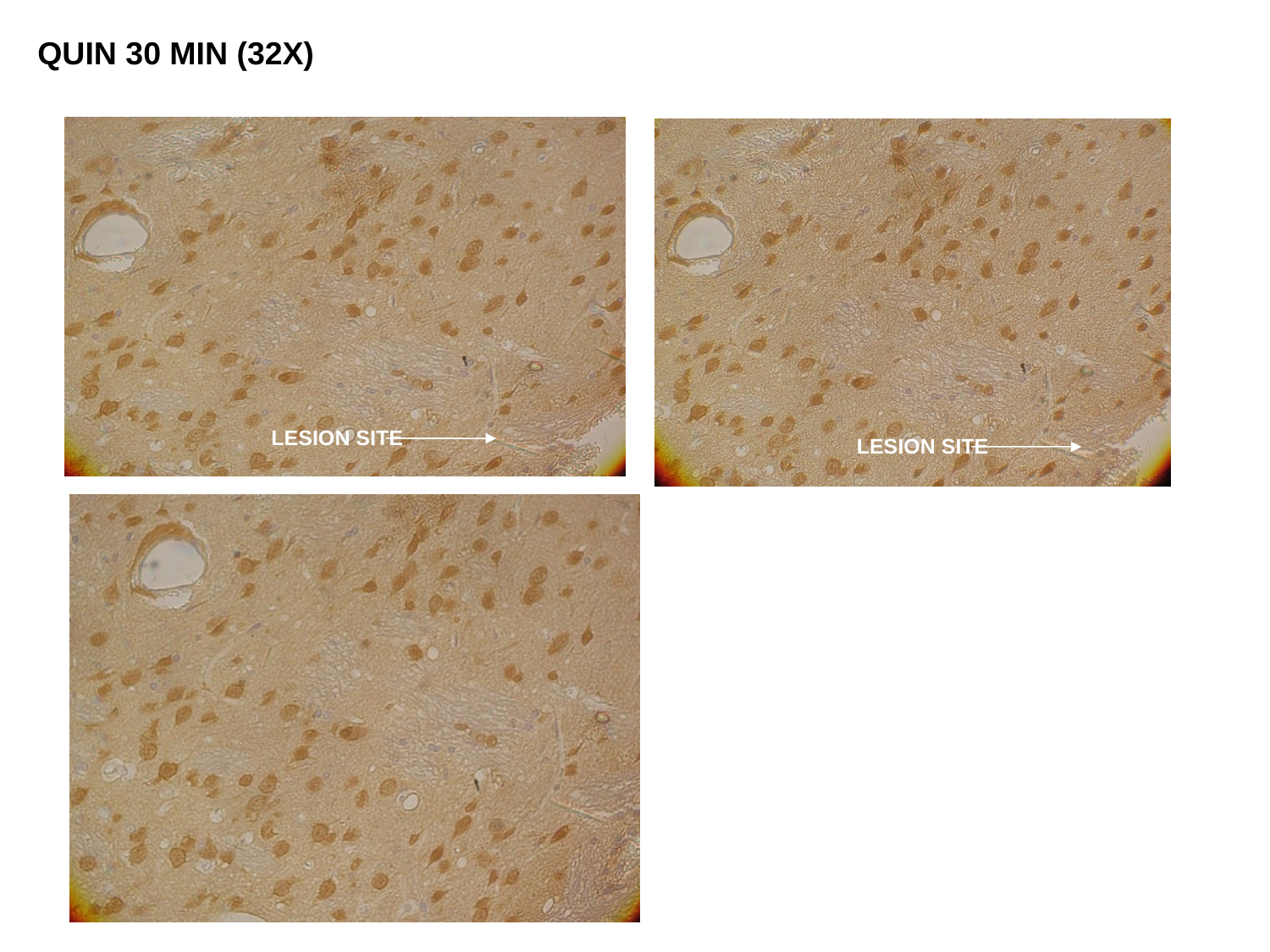

QUIN 30 MIN (32X)
LESION SITE
LESION SITE

## Slide 19
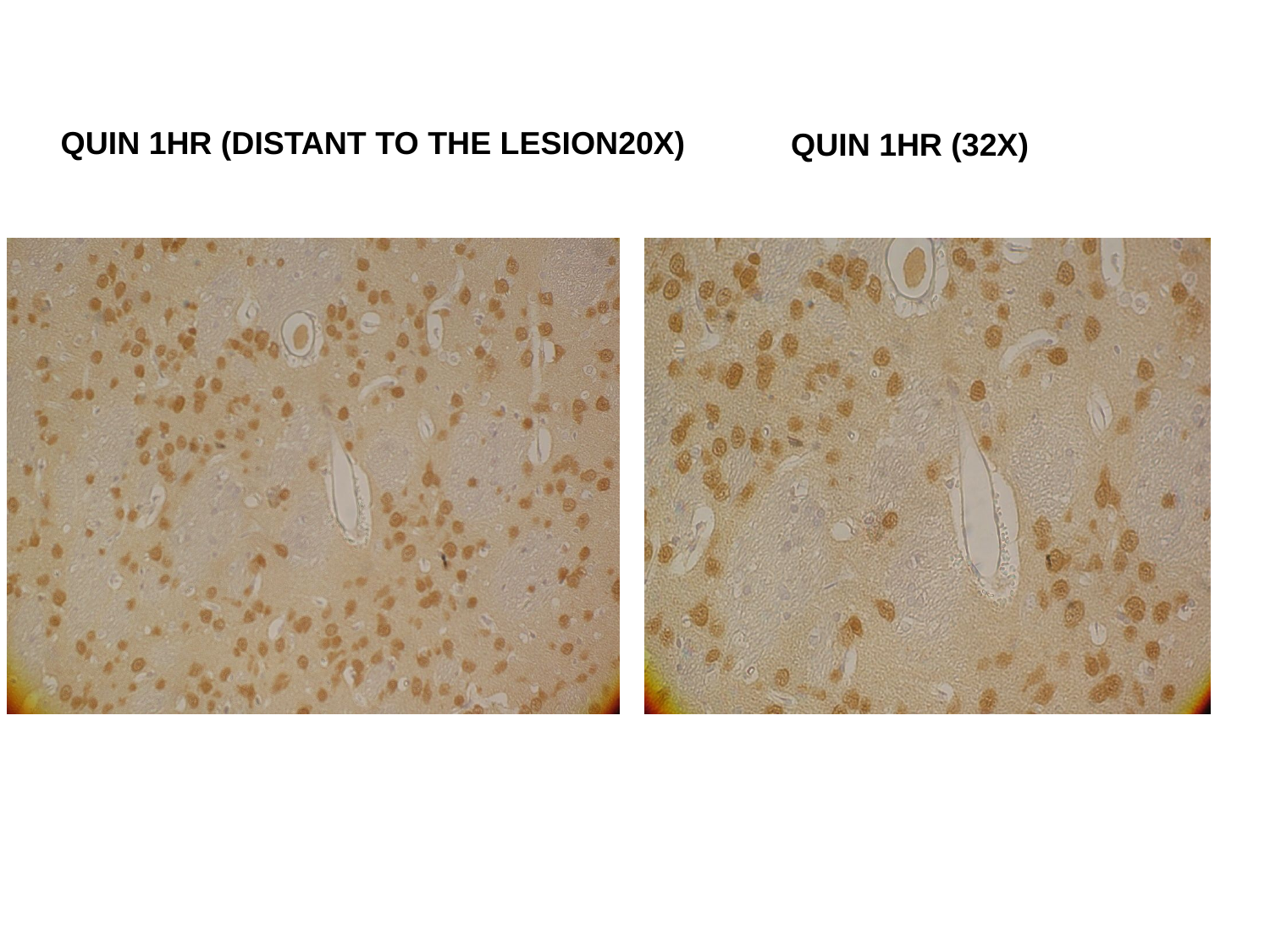

QUIN 1HR (DISTANT TO THE LESION20X)
QUIN 1HR (32X)

## Slide 20
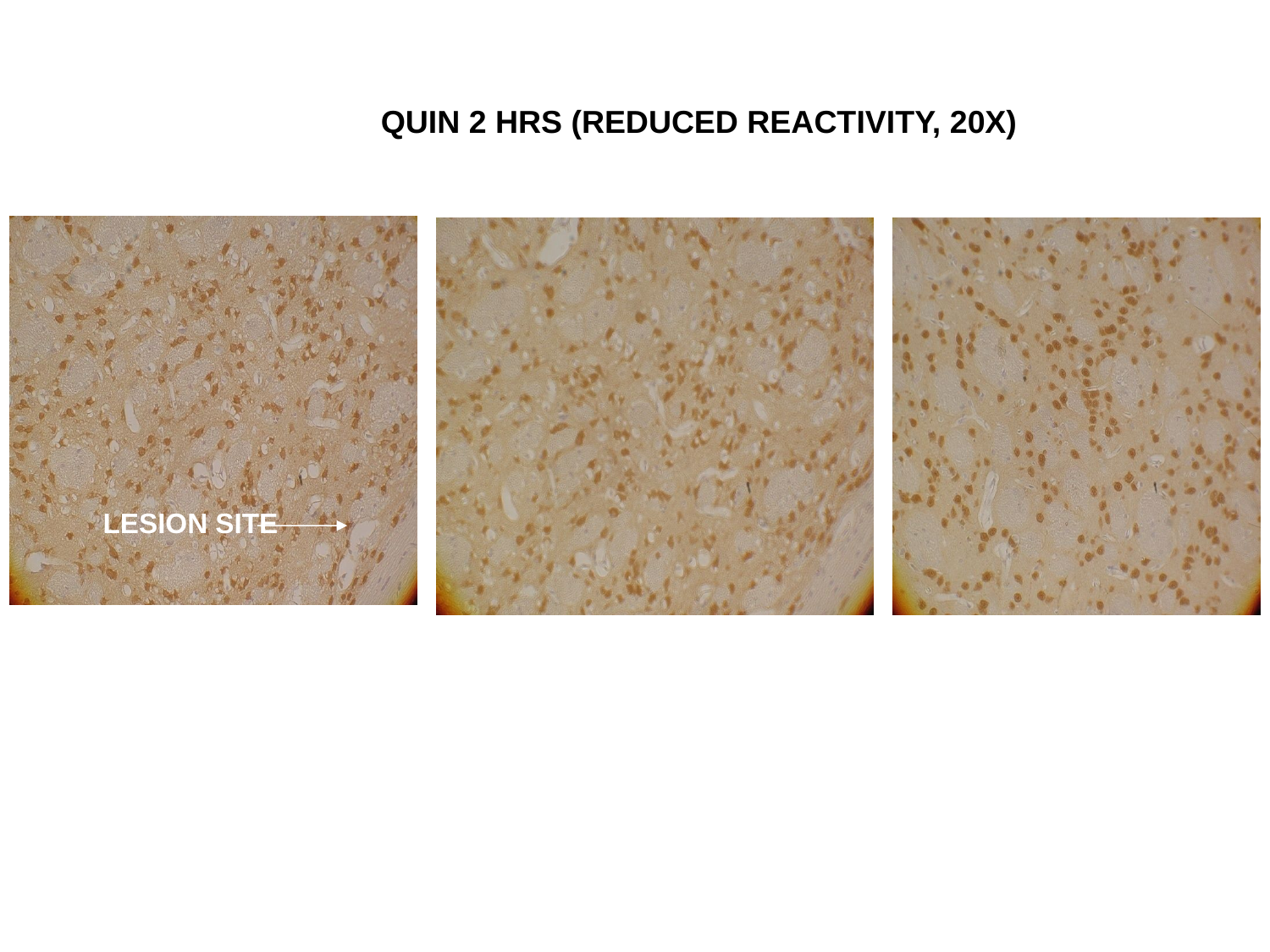

QUIN 2 HRS (REDUCED REACTIVITY, 20X)
LESION SITE

## Slide 21
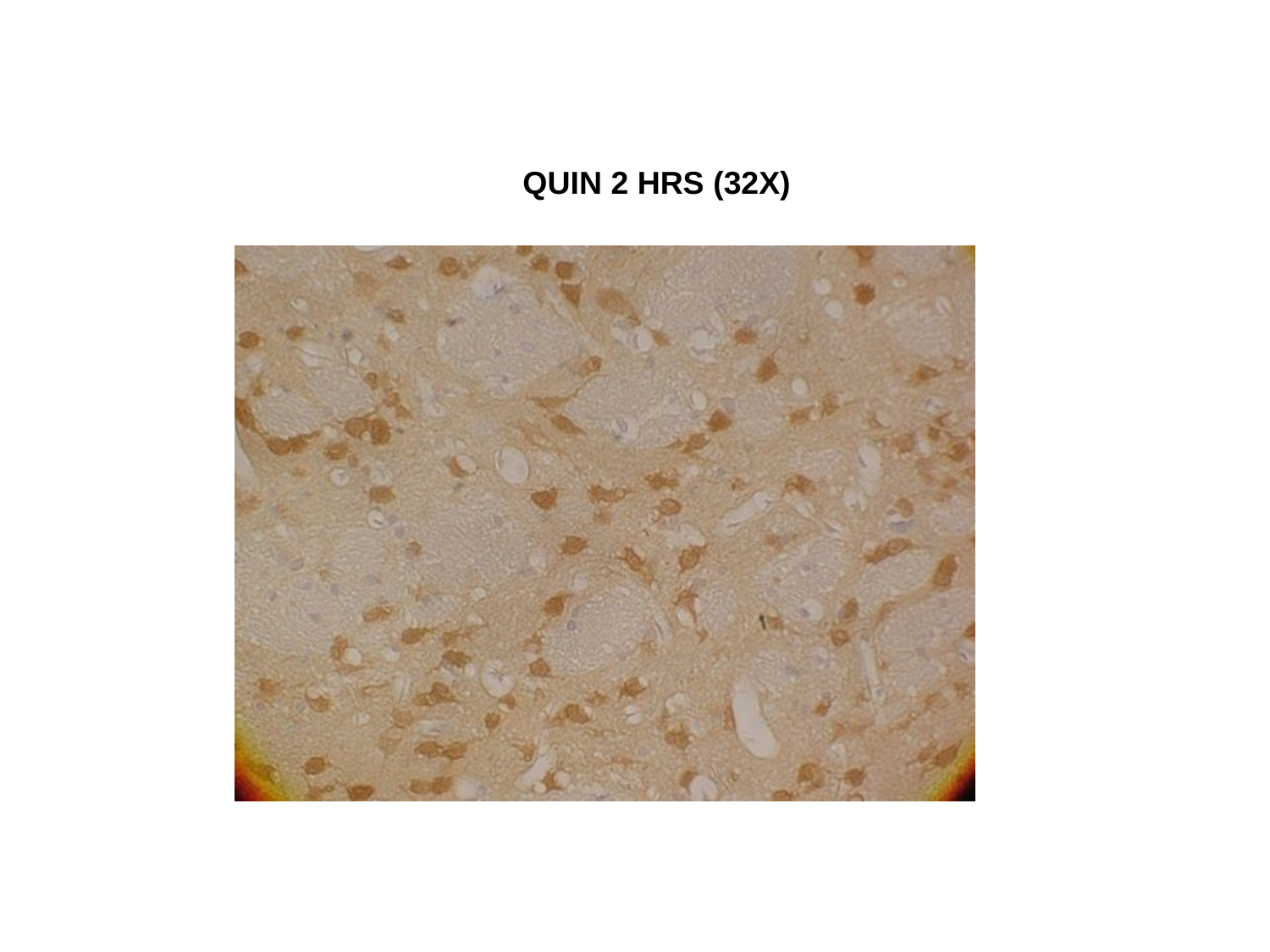

QUIN 2 HRS (32X)

## Slide 22
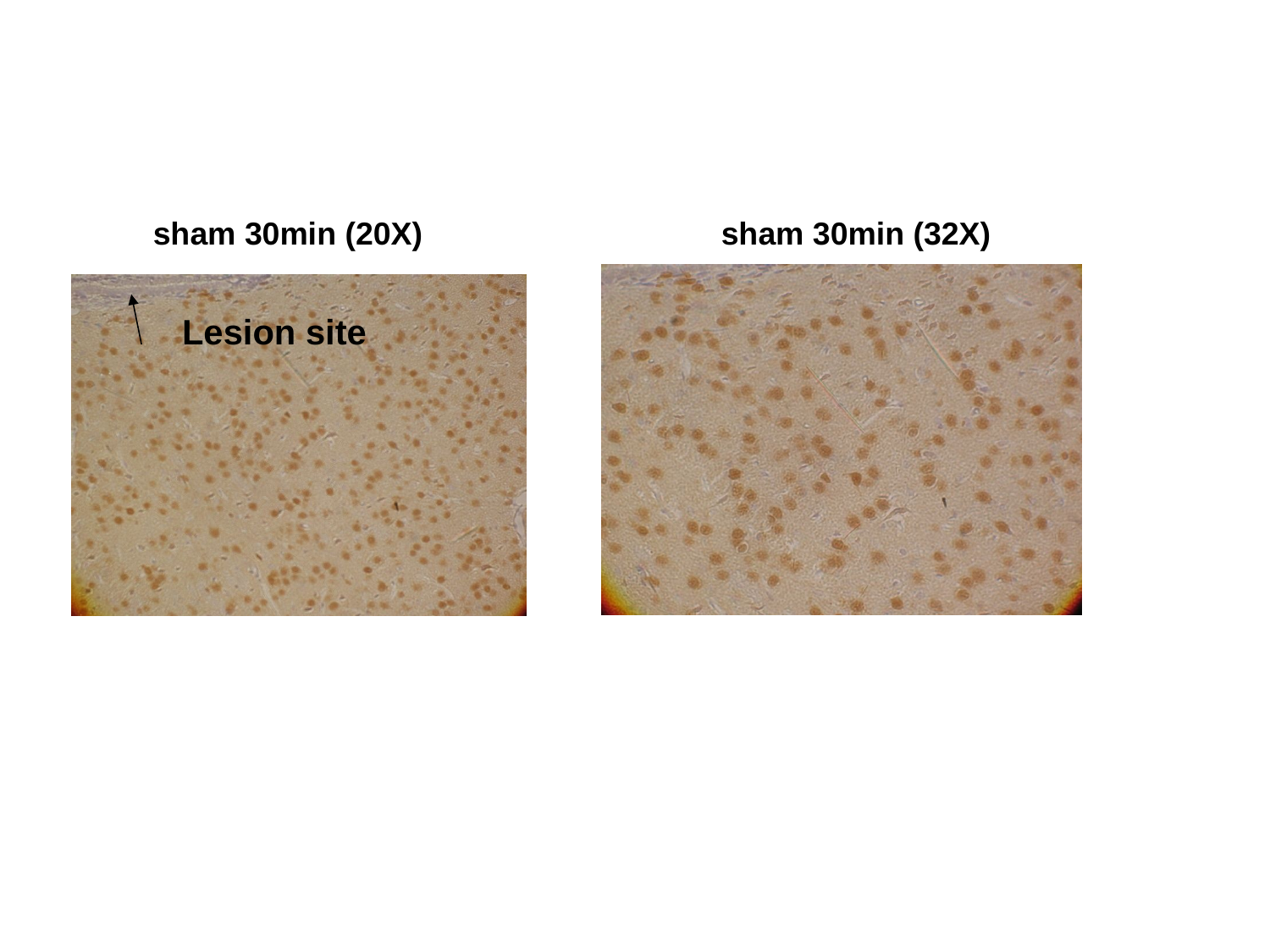

sham 30min (20X)
sham 30min (32X)
Lesion site

## Slide 23
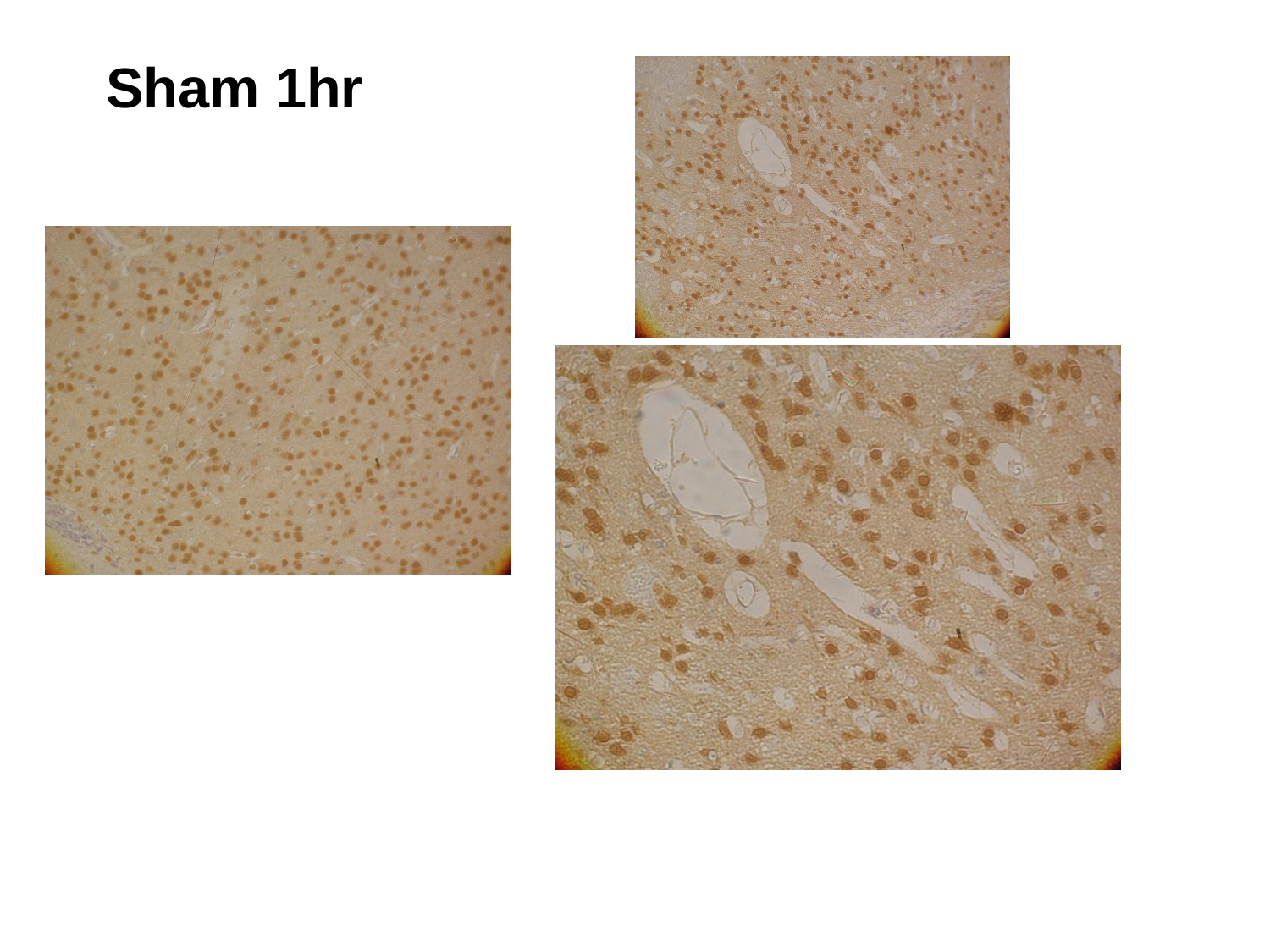

Sham 1hr

## Slide 24
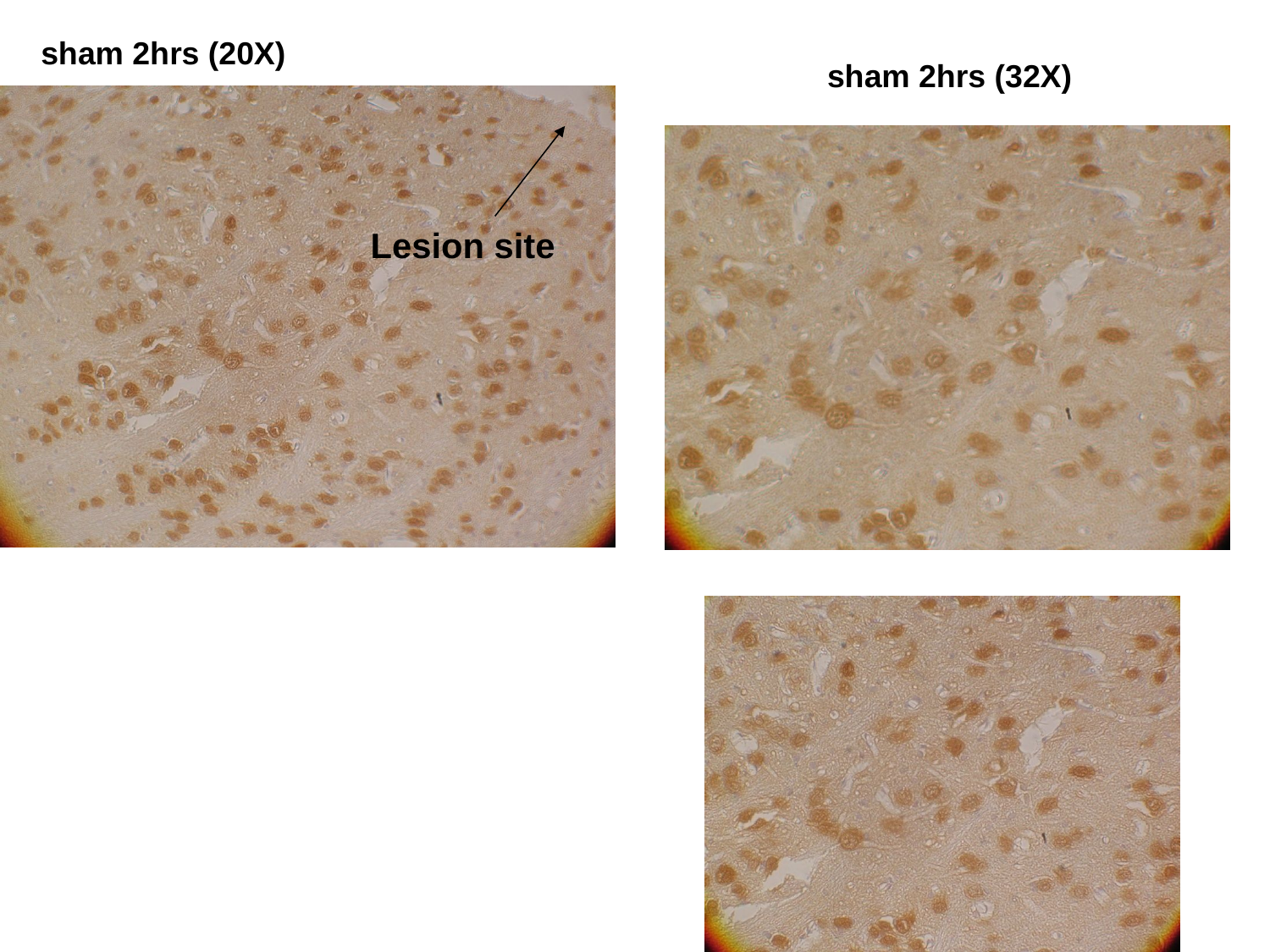

sham 2hrs (20X)
sham 2hrs (32X)
Lesion site
