# Supplementary material for: Expression of Concern: Modeling the Interaction between Quinolinate and the Receptor for Advanced Glycation End Products (RAGE): Relevance for Early Neuropathological Processes
Source: PLoS One. 2023 Feb 14;18(2):e0281905. doi: 10.1371/journal.pone.0281905 (PMC9928092; doi:10.1371/journal.pone.0281905)

**S5 File: Original data underlying results of Figure 5 (original and repeat employed for calculation)**


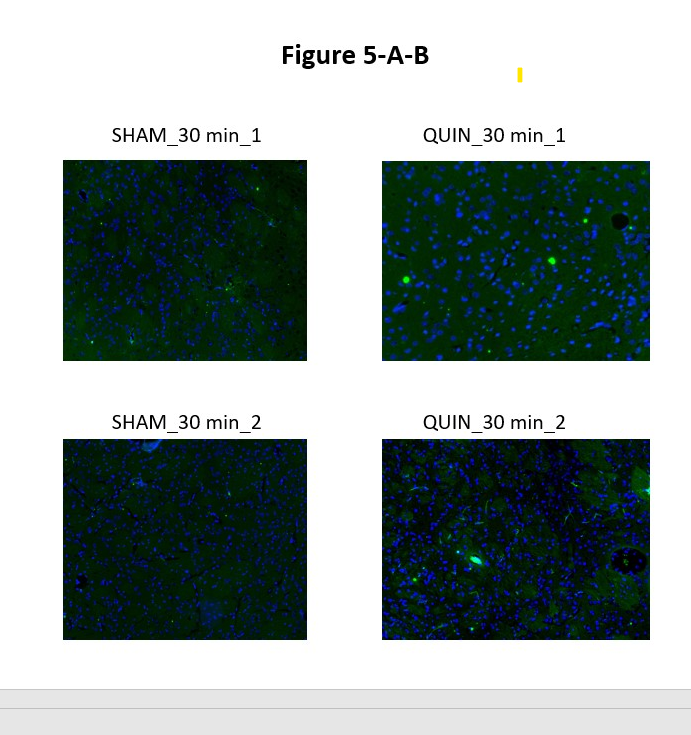


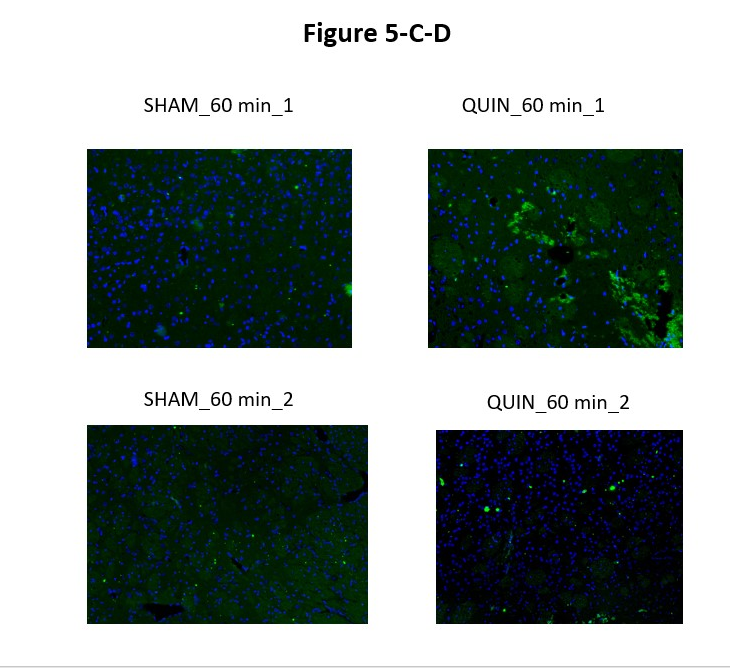


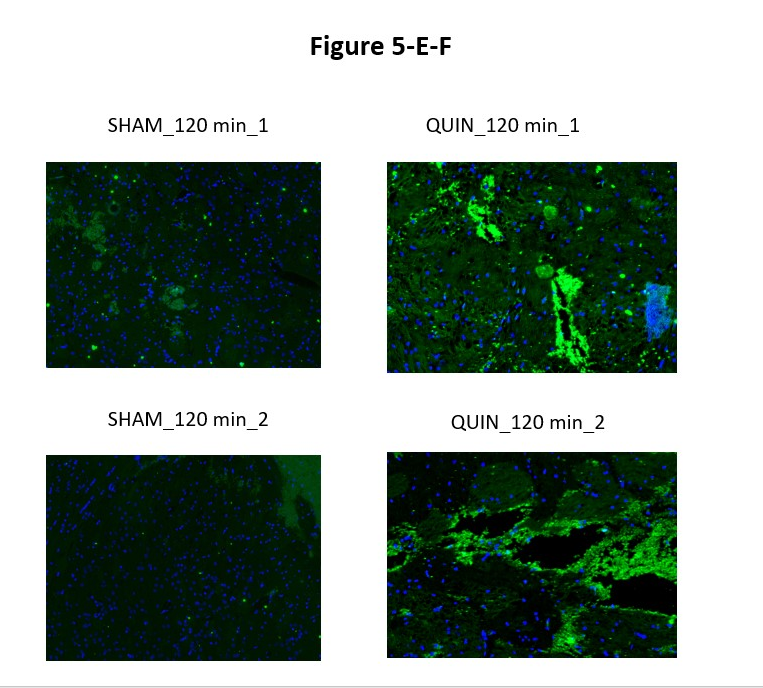


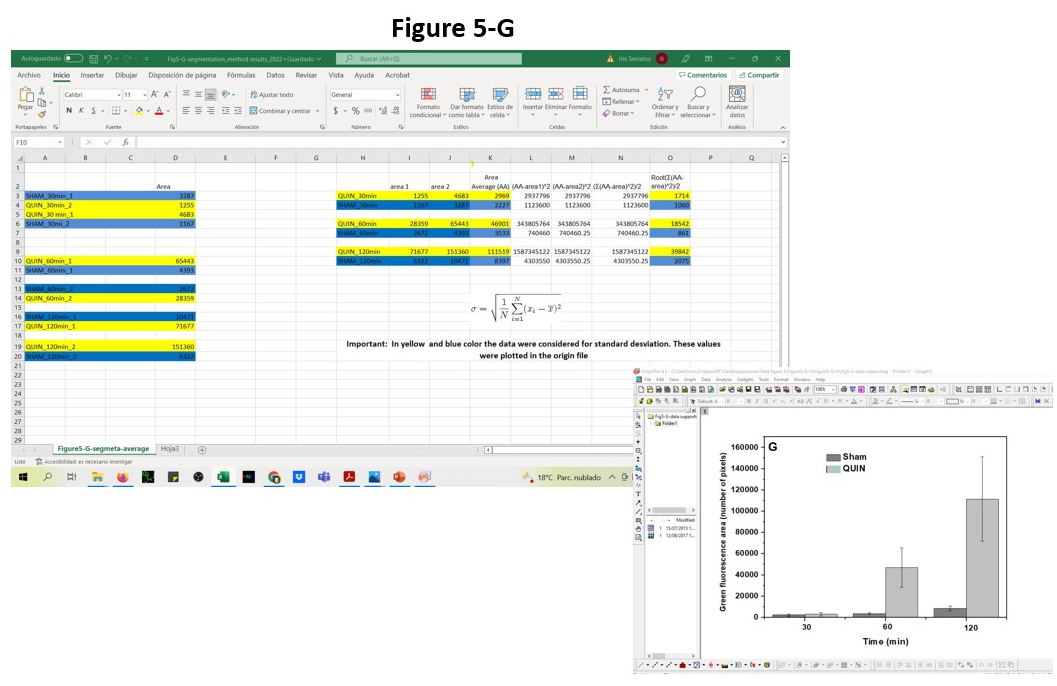


**This figure was constructed using segmentation data**


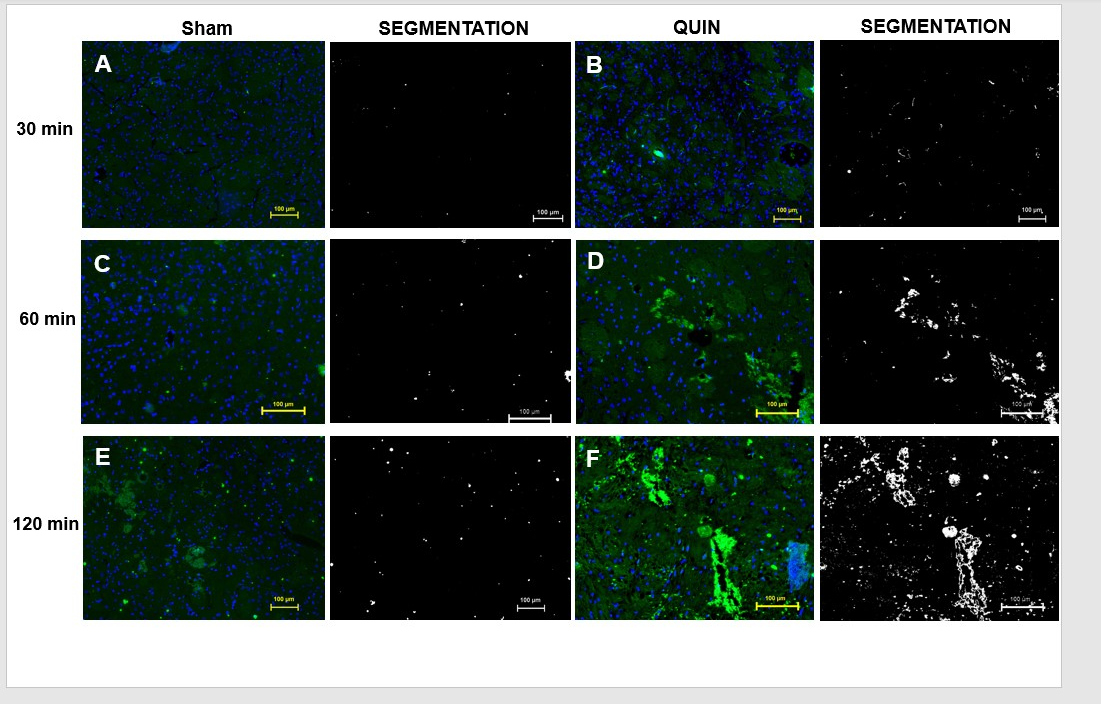


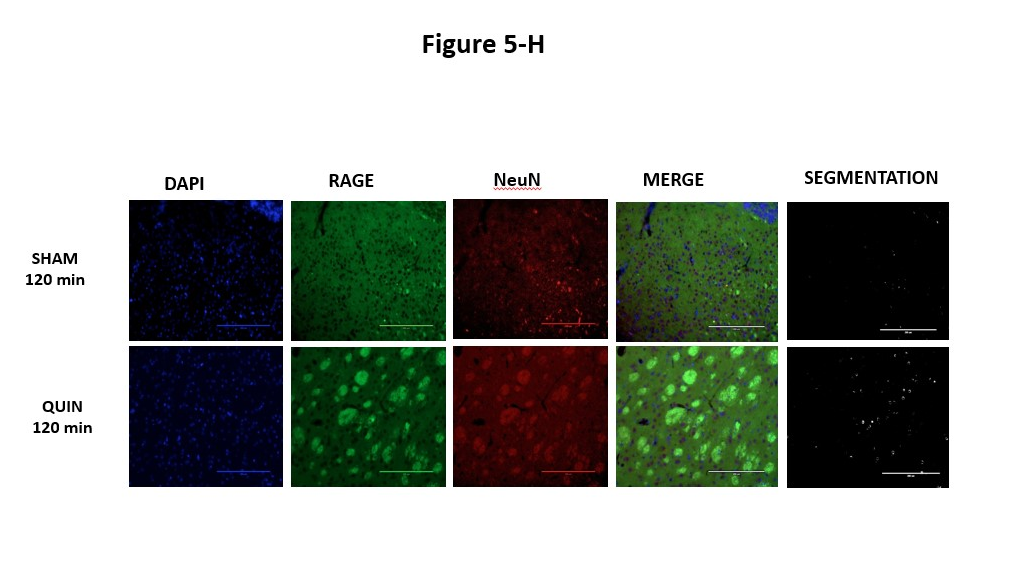

Supplement: S5 File — (DOCX) [file pone.0281905.s005.docx]
