# Supplementary material for: Expression of Concern: Modeling the Interaction between Quinolinate and the Receptor for Advanced Glycation End Products (RAGE): Relevance for Early Neuropathological Processes
Source: PLoS One. 2023 Feb 14;18(2):e0281905. doi: 10.1371/journal.pone.0281905 (PMC9928092; doi:10.1371/journal.pone.0281905)

12-07-2014.

Resumen 2 CORREGIDO

Ajustes de Titulación Fluorimétrica

Archivos: sVC1 / Ac. Quin   Folder: F.Reyes / I.Serratos Febrero 2014

	a Value	a ± Std Error	K_d (µM) Value	k ± (µM) Standard Error	Statistics Red Chi-Sqr	Statistics Adj. R-Square	K_a (M^{-1})	k + (M^{-1})
			**pH 9.0**					
B	0.47206	0.01448	0.01833	0.00578	1.16E-03	0.93797	5.46E+07	1.72E+07
D	0.47289	0.01296	0.02475	0.00596	8.51E-04	0.95603	4.04E+07	9.73E+06
F	0.41057	0.01296	0.04139	0.00888	7.02E-04	0.95134	2.42E+07	5.18E+06
H	0.4093	0.01332	0.04353	0.0094	7.26E-04	0.94869	2.30E+07	4.96E+06
J	0.36223	0.00965	0.04649	0.008	3.74E-04	0.9675	2.15E+07	3.70E+06
L	0.34295	0.00956	0.04745	0.00846	3.64E-04	0.96547	2.11E+07	3.76E+06
			**pH 7.4**					
N	0.25821	0.01065	0.0172	0.00725	5.44E-04	0.90124	5.81E+07	2.45E+07
P	0.25911	0.00783	0.04462	0.00887	2.50E-04	0.9621	2.24E+07	4.46E+06
R	0.16845	0.00682	0.04366	0.01121	1.55E-04	0.93637	2.29E+07	5.88E+06

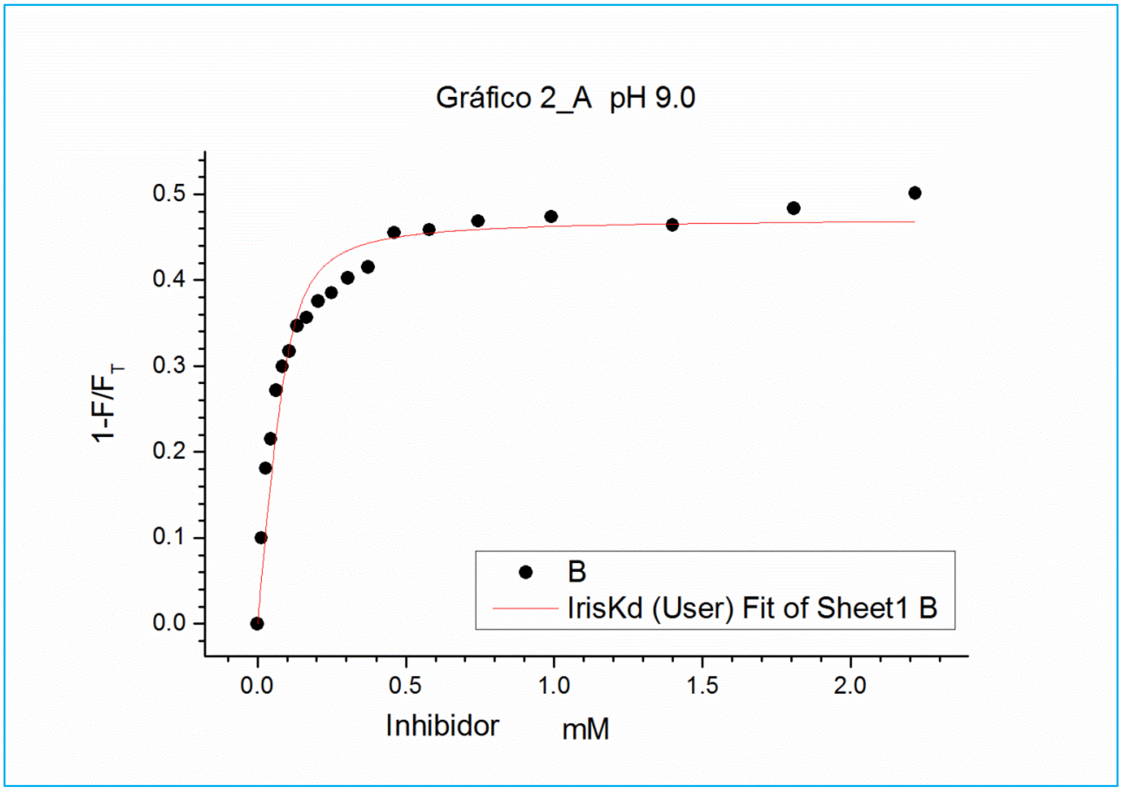

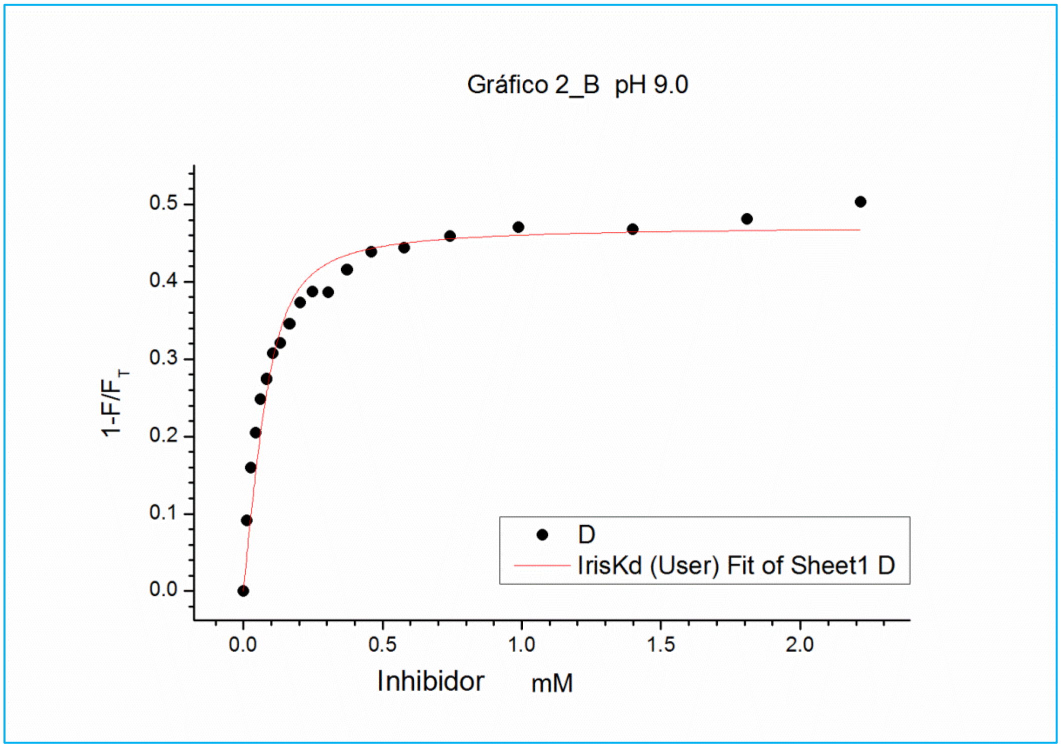
Gráfico 2_B pH 9.0
1-F/F_T
0.5
0.4
0.3
0.2
0.1
0.0
0.0
0.5
1.0
1.5
2.0
Inhibidor mM
D
IrisKd (User) Fit of Sheet1 D

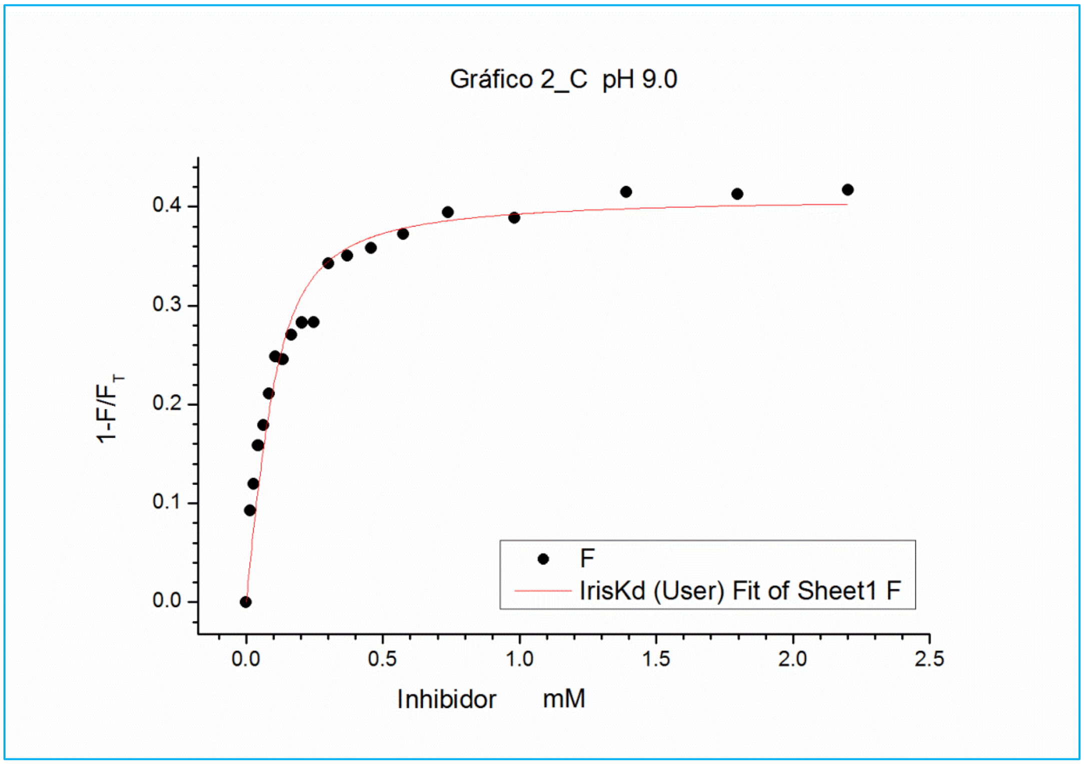
Gráfico 2_C pH 9.0
1-F/F_T
0.4
0.3
0.2
0.1
0.0
0.0
0.5
1.0
1.5
2.0
2.5
Inhibidor mM
F
IrisKd (User) Fit of Sheet1 F

Figure 7b-Titration fluorescence

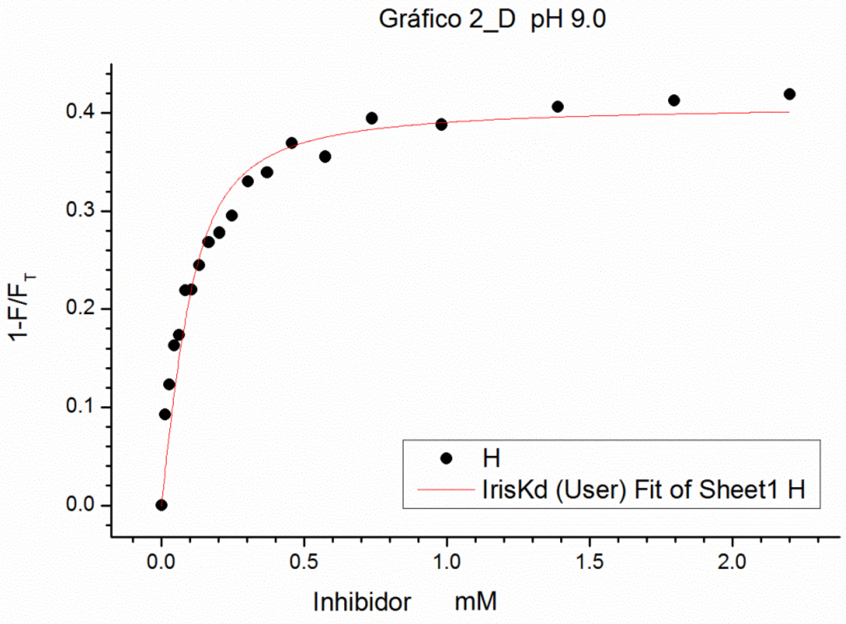

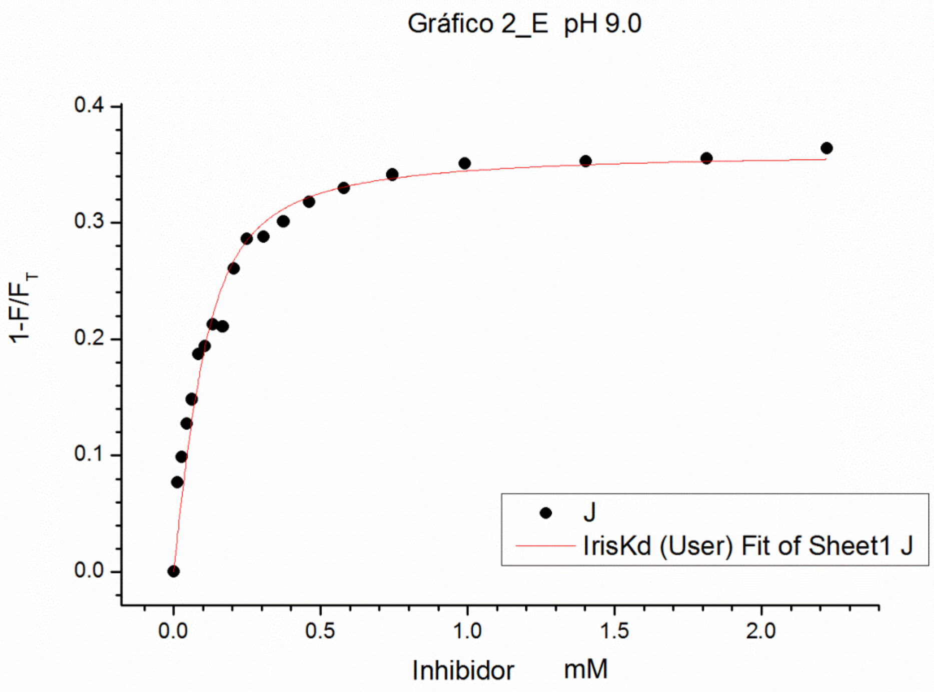

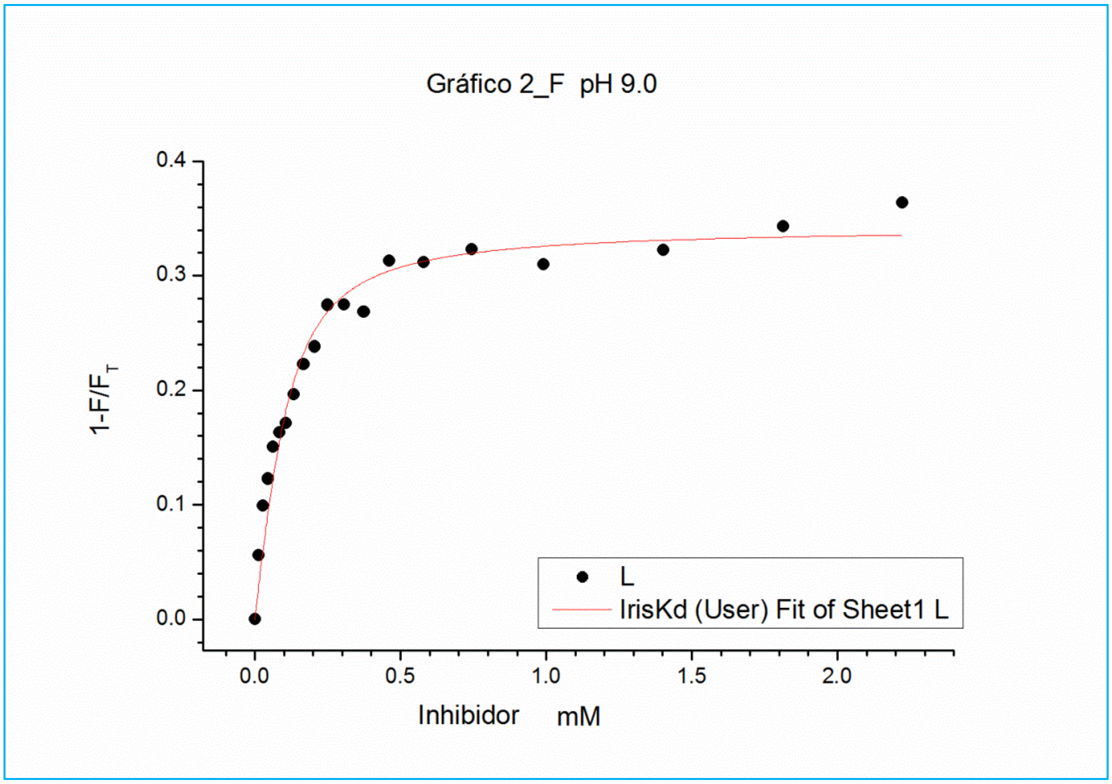
Gráfico 2_F pH 9.0
1-F/F$_T$
Inhibidor mM
L
IrisKd (User) Fit of Sheet1 L
0.0
0.1
0.2
0.3
0.4
0.5
1.0
1.5
2.0

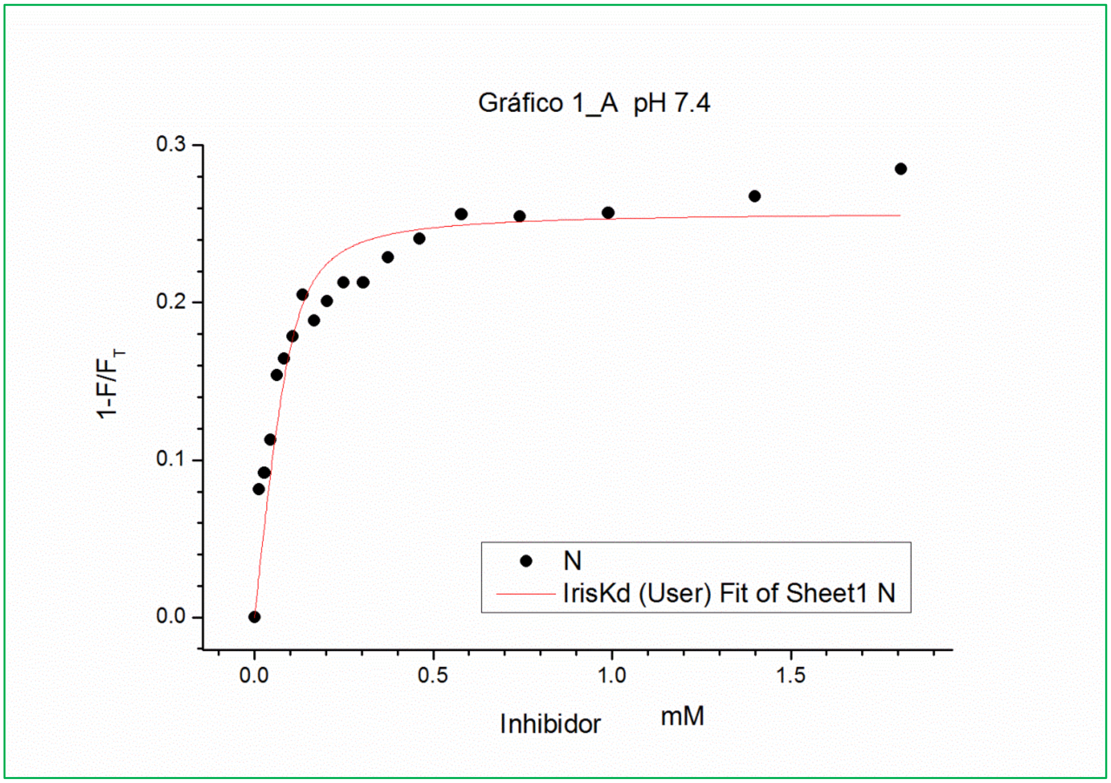
Gráfico 1_A pH 7.4
1-F/F$_T$
Inhibidor mM
N
IrisKd (User) Fit of Sheet1 N
0.0
0.1
0.2
0.3
0.5
1.0
1.5

Figure 7a-Titration fluorescence

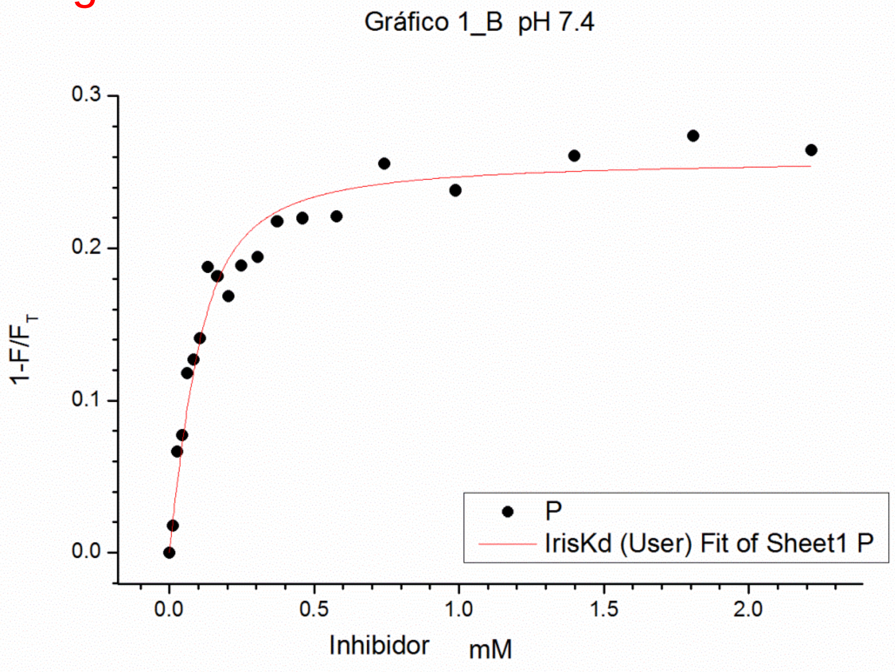

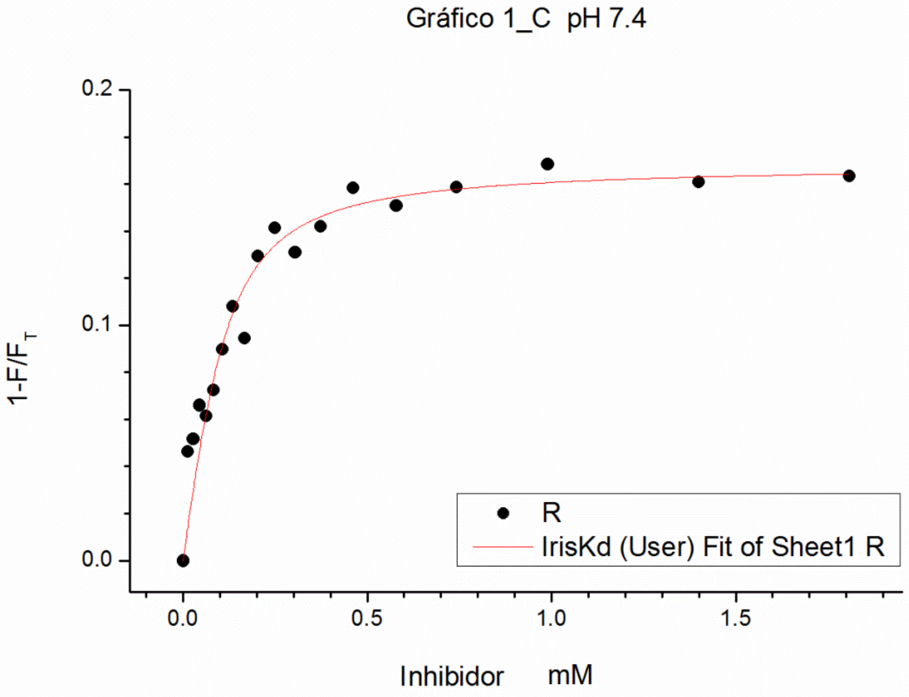

Supplement: S7 File — (PDF) [file pone.0281905.s007.pdf]
